# Synthesis of structural analogues of Reversan by ester aminolysis: an access to pyrazolo[1,5-*a*]pyrimidines from chalcones†

**DOI:** 10.1039/d3ra02553e

**Published:** 2023-05-31

**Authors:** Andres Arias-Gómez, Mario A. Macías, Jaime Portilla

**Affiliations:** a Department of Chemistry, Bioorganic Compounds Research Group, Universidad de Los Andes Carrera 1 No. 18A-10 Bogotá 111711 Colombia jportill@uniandes.edu.co; b Department of Chemistry, Crystallography and Chemistry of Materials, Universidad de Los Andes Carrera 1 No. 18A-10 Bogotá Colombia

## Abstract

Reversan, a multidrug resistance-associated protein (MRP1) inhibitor described more than a decade ago, is a commercial drug (CAS: 313397-13-6) that has a high price and is six to eight times more potent than known drug transporter inhibitors. However, to date, a complete route for synthesizing pyrazolo[1,5-*a*]pyrimidine-based Reversan is yet to be published. Herein, the silica gel-mediated synthesis of Reversan and a novel family of its structural analogues (amides) *via* the microwave-assisted amidation reaction of 3-carboethoxy-5,7-diphenylpyrazolo[1,5-*a*]pyrimidine (ester) with primary amines is reported. Moreover, a set of this ester-type precursor was obtained using the NaF/alumina-mediated reaction of 5-amino-3-carboethoxy-1*H*-pyrazole with chalcones, implying a final removal of H_2_ using Na_2_S_2_O_8_. Both esters and amides were obtained in high yields using heterogeneous catalyst and solvent-free, highly efficient, and scalable synthetic protocols.

## Abstract 2

NaF/Al_2_O_3_ and SiO_2_-mediated solvent-free syntheses of Reversan and similar compounds, first cyclizing chalcones with 5-aminopyrazoles under fusion heating and then by the direct amidation reaction of esters under microwaves conditions, are reported.

## Graphical abstract

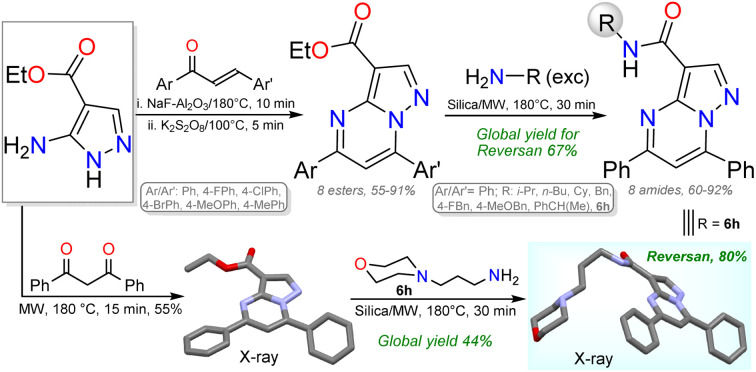

## Introduction

**Fig. 1:**
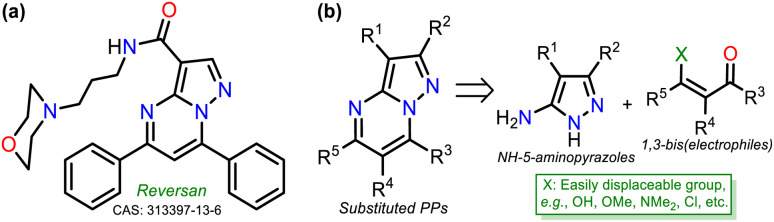
(a) Molecular structure of Reversan and (b) retrosynthetic analysis of PPs.

Intrinsic or acquired multidrug resistance is one of the leading causes of treatment failure in human malignancies; thus, finding new ways to solve this problem has attracted special attention from chemists, biologists, pharmacists, and related professionals. Molecular-level investigations of cancer multidrug resistance have revealed that two ATP-binding cassette transporters cause resistance in tumor cells: *P*-glycoprotein and the multidrug resistance-associated protein (MRP1).^1,2^ The overexpression of MRP1 in almost all tumor types (*e.g.*, lung, melanoma, sarcoma, neuroblastoma, head, and breast) lowers the intracellular drug concentration.^3–5^ About this, Burkhart *et al.*^6^ reported a way to overcome MRP1 activity using Reversan, a pirazolo[1,5-*a*]pyrimidine (PP) derivative having an *N*-(3-morpholinopropyl)carboxamide group at position 3 and two phenyl rings at positions 5 and 7, which is commercially available but has a high price (Fig. 1a).

**Scheme 1:**
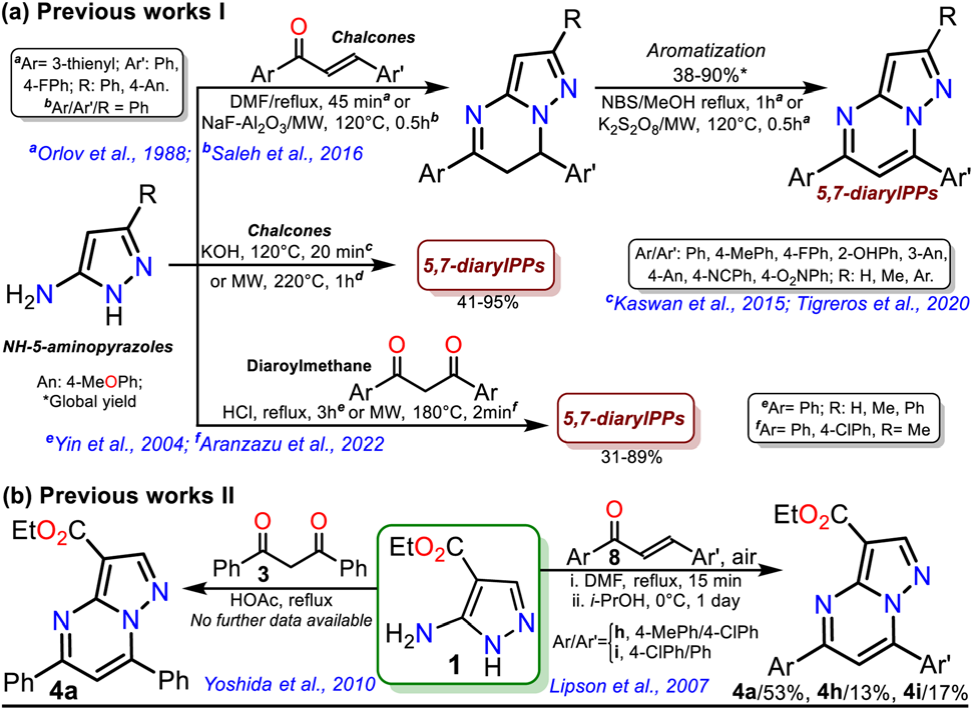
Synthesis of 5,7-diaryl PPs using (a) 5-aminopyrazoles or (b) amimoester 1.

Reversan is six to eight times more potent in inhibiting MRP1 than known drug carrier inhibitors (*i.e.*, verapamil, difloxacin, probenecid, and PAK104P).^6^ Despite the high biological effects described over a decade ago, a synthetic route for Reversan or its structural analogues is yet to be reported. Hence, obtaining this family of pyrazolo[1,5-*a*]pyrimidines (PPs) is challenging. In this line, our group has focused on obtaining diverse functionalized PPs,^7,8^ mostly investigating their photophysical properties as a promising approach,^9,10^ adding to recurring biological applications of these compounds.^10–13^ PP ring access is usually achieved by constructing the pyrimidine moiety *via* the cyclocondensation reaction of *NH*-5-aminopyrazoles with 1,3-bis(electrophiles) such as ynones, β-dicarbonyls, β-enamionones, and β-ketonitriles, which permit the involved unsaturation in products (Fig. 1b).^10–13^ However, although chalcones (enones) yield 5,7-diaryl derivatives with greater molecular diversity than similar substrates (*e.g.*, β-diketones such as diaroylmethane), enones are rarely used because mixtures of products with the dihydro derivative (or perhaps some regioisomer) are obtained; more rigorous conditions or further steps are required to obtain the aromatic ring (Scheme 1a).^8,14–18^

The poor aromatic character of the pyrimidine ring inside the PP core possibly is an insignificant driving force that favors π-conjugation in products.^10–13^ As a result, there are even rarer examples for synthesizing PPs from chalcones 2 and *NH*-5-aminopyrazoles bearing an electron-withdrawing group (EWG), such as ethyl-5-amino-1*H*-pyrazole-4-carboxylate (1), which is a crucial substrate in Reversan synthesis since they are poorly reactive 1,3-bis-(nucleophiles).^19^ To date, only four articles where precursor 1 gives 5,7-diarylpyrazolo[1,5-*a*]pyrimidines 4 have been published; in two of these, an acid-mediated tricomponent reaction with arylaldehydes and arylacetylene was carried out,^20,21^ while in the other two, chalcones 2 (ref. 22) or dibenzoylmethane (3)^23^ were used as substrates (Scheme 1b). However, in these last two works, the results and characterization data are unsatisfactory.

**Scheme 2:**
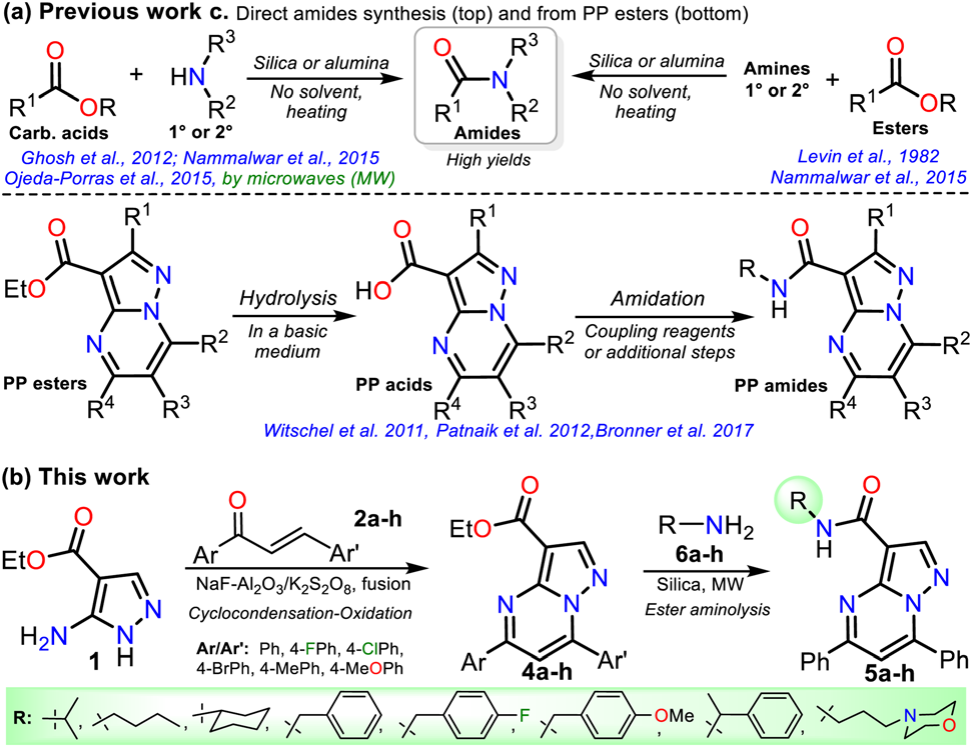
(a) Synthesis of amides by solid-supported catalysts and using PP esters. (b) Proposal for synthesizing reversan (5h) and its structural analogues.

On the other hand, ester aminolysis is possibly the least common method for forming amides, one of the most appreciated functional groups in biologically relevant compounds.^24–28^ The low recurrence of direct ester amidation is possibly because the reactions require high temperatures. Under this condition, substrates are susceptible to decomposition or easy evaporation, especially liquid ones, leading to a loss of mass efficiency or low reaction yields.^24–26^ The usual methods for preparing amides involve reacting carboxylic acids or acylating agents (*i.e.*, acid anhydrides, acid chlorides, and acids with additives) with amines. However, most of these methods require excess amine, catalysts, coupling reagents, solvents excess, or high temperatures with prolonged reaction times, leading to protocols with a poor atom economy, generating toxic wastes, and spending energy.^24–29^ Thus, the solvent-free direct amidation reaction of carboxylic acids or esters by heterogeneous catalysis using silica or alumina is a suitable method, though little-used, to efficiently access amides due to the value and easy disposal of these solids (Scheme 2a).^29–33^

Due to the biological importance of pyrazolo[1,5-*a*]pyrimidines^10–13^ and the amide functional group,^24–28^ there are several examples of synthesis of this *N*-heterocycle substituted at position 3 with the carboxamide group. However, when the respective ester is used as a starting reagent, the synthesis proceeds by hydrolysis and subsequent amidation under conditions that imply coupling agents, further reaction steps, and poor yields (Scheme 2b).^34–36^ Pondering these findings and the relevance of reversan, we envisioned that this amide and a novel family of its structural analogues (PPs 5a–h) could be synthesized through the direct amidation reaction of ethyl-5,7-diphenylpyrazolo[1,5-*a*]pyrimidine-3-carboxylate (4a) with primary alkylamines 6a–h. Likewise, due to the moderate use of chalcones in PPs syntheses and our interest in accessing this type of heterocycle by eco-compatible methods from easily accessible reagents,^7–10,17^ we also proposed obtaining the 5,7-diarylsubstituted esters 4a–h by the reaction of 1 with chalcones 2a–h (Scheme 2c). Ultimately, we look for the synthesis of 4a–h and 5a–h through green approaches.

## Results and discussion

### Synthesis of pyrazolo[1,5-*a*]pyridimidines 4a–h

**Scheme 3:**
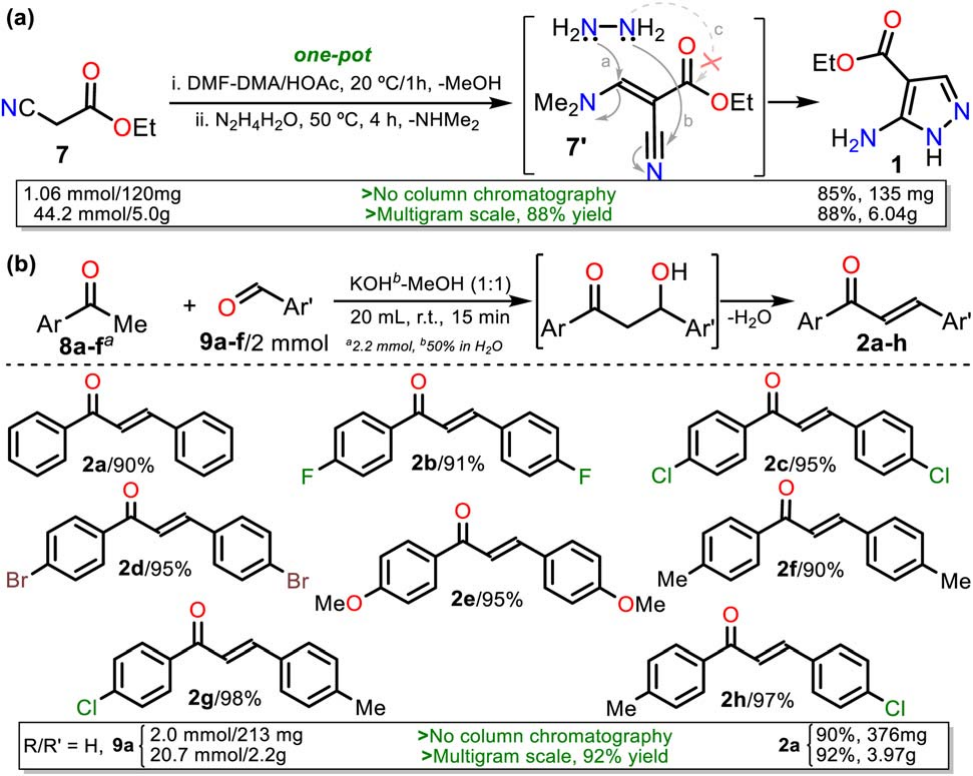
Synthesis of (a) aminoester 1 and (b) chalcones 2a–h.

We started our investigation by preparing precursors such as ethyl-5-amino-1*H*-pyrazole-4-carboxylate (1) and chalcones 2a–h*via* known protocols that resulted in high yields. Aminoester 1 was obtained as white crystals after its recrystallization from ethanol using a one-pot approach starting with the reaction of ethyl cyanoacetate (7) and DMF-DMA to access the 1,3-bis(electrophilic) intermediate 7′; then, upon adding hydrazine monohydrate (HM), the cyclocondensation reaction of this with the β-enaminonitrile moiety of 7′ (route a + b), instead of its less reactive β-enaminoester fragment (a + c), occurs to access the desired product in a regioselective manner (Scheme 3a).^19,37^ Chalcones 2a–h were prepared by the Claisen–Schmidt condensation reaction between acetophenone 8a–f and arylaldehydes 9a–f in methanol/water (1 : 1 v/v), a basic medium, short reaction times, and simple purification by recrystallization (Scheme 3b).^38^

**Scheme 4:**
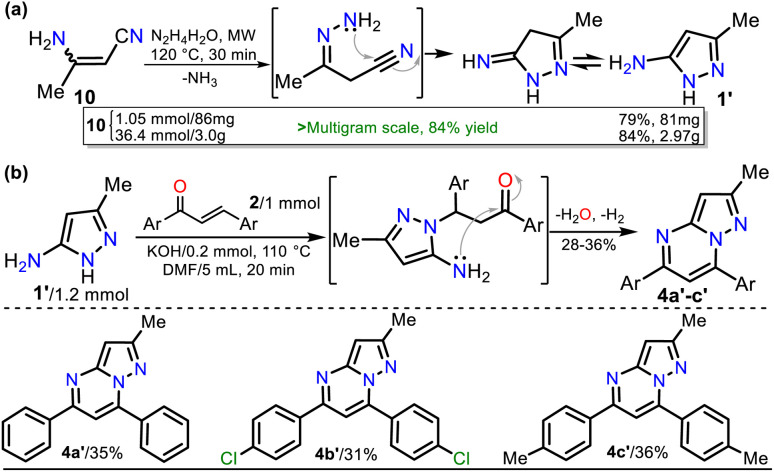
Synthesis of (a) 3-methyl-1*H*-pyrazol-5-amine (1′) and (b) PPs 4a′–b′.

On the other hand, 3-methyl-1*H*-pyrazol-5-amine (1′)^39^ was prepared to examine the best protocol reported^16^ to access PPs starting from chalcones (see Scheme 1a above). Amine 1′ was synthesized from 3-aminocrotononitrile (10) and HM (1.5 equiv.) by a modified method at 120 °C for 30 min under microwave; amine 1′ was purified by flash chromatography (Scheme 4a). Subsequently, we carried out the syntheses of 1, 2a, and 1′ on a scale of about 6, 4, and 3 g, respectively, as they are strategic substrates in our laboratory (Schemes 3 and 4a, data in rectangles). Notably, precursors 1 and 2a were crucial for this work and obtained without chromatographic purification. In addition, increasing the scale also resulted in a slight increase in yields of 1 (from 85 to 88%), 2a (from 90 to 92%), and 1′ (from 79 to 84%).

With the required precursors in hand, we envisaged that the reaction of 1 and 2a–h could give 3-carboethoxypyrazolo[1,5-*a*]pyrimidines 4a–h by the standard route, related to previous works (see Schemes 1 and 2b). In this way, we reproduce the synthesis of 4a′ by Kaswan *et al.*,^16^ where they obtained the product in 82% yield from the amine 1′, chalcone (2a) and KOH as a catalyst in DMF; however, we obtained moderate yield despite the experimental variants used (*i.e.*, time and MW heating). Then, we obtained two other PPs (4b′–c′), but the results did not improve; thus, although products can be obtained as reported, this protocol must be revised (Scheme 4b). These results made us question the reactivity of chalcones toward aminoester 1, which is even less nucleophilic than amine 1′.

In general, obtaining the PP esters 4a–h using chalcones 2a–g and aminoester 1 is a great challenge due to the reactivity of substrates, the easy access to 2a–g from cheap reagents, and even more due to the absence of a standard method for this synthesis. Thus, we started the study by exploring the reaction of 1 with an equimolar amount of 2c (Ar = 4-ClPhl) to optimize this reaction. We selected 2c due to its high electrophilic character, and the chlorophenyl group generally allows us simple purification processes and follow-up by ^1^H NMR. By thin-layer chromatography (TLC), we noted that reactions did not proceed or occur with poor conversion under similar conditions to those used in our laboratory for similar reactions, *i.e.*, without or with polar solvent under microwave, allowing us to carry out several tests quickly.^40^ Similar results were evidenced under heating to reflux and using non-nucleophilic bases. Decomposition products were obtained by heating the reaction above 180 °C (Table 1, entries 1 to 4).

**Table 1:** Optimization of the synthesis of the pyrazolo[1,5-*a*]pyrimidine 4c^a^

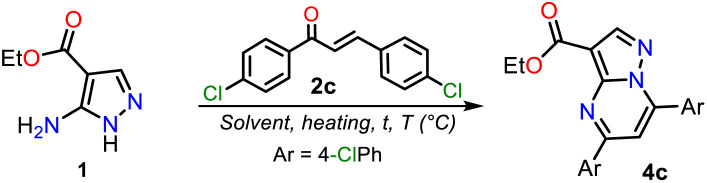				
Entry	Solvent and/or additive	*T* (°C)	Time *t*	Yield (%)
1	S-F, DMF, HOAc, or EtOH	120–200^b^	60 min	Traces
2	DMF, HOAc, or EtOH	Reflux	24 h	Traces
3	S-F, DMF/Cs_2_CO_3_, or Et_3_N (1 equiv.)	180^b^	60 min	NR
4	DMF, Cs_2_CO_3_, or Et_3_N (1 equiv.)	Reflux	24 h	Traces
5	Silica gel (100 mg)	130^b^	15 min × 3	12
6	Al_2_O_3_ (100 mg)	200^b^	15 min × 4	6
7	NaF–Al_2_O_3_ (0.2 : 1, 100 mg)^c^	180^b^	15 min × 4	14
8	NaF–Al_2_O_3_ (0.4 : 1, 100 mg)^c^	180^b^	15 min × 4	20
9	NaF–Al_2_O_3_ (0.6 : 1, 100 mg)^c^	180^b^	15 min × 4	40
10	NaF–Al_2_O_3_ (0.6 : 1, 50 mg)^c^	180^b^	15 min × 2	24
11	NaF–Al_2_O_3_ (0.6 : 1, 10 mg)^c^	180^b^	15 min × 4	62
12	NaF–Al_2_O_3_ (0.6 : 1, 10 mg)^c^	180^d^	10 min	81
13	NaF–Al_2_O_3_ (0.6 : 1, 10 mg)	180^d^	10 min	43

a Reactions conditions: 1 and 2c (0.2 mmol) in 1.0 mL solvent under heating in a mantle: NR = no reaction.

b Run in a 10 mL sealed tube under MW in 0.5 mL solvent or solvent-free (S-F) conditions.

c K_2_S_2_O_8_ (0.2 mmol) is added, and the reaction is heated for another 5 min at 100 °C.

d A tubular reaction vessel was charged with the mixture and heated by a sand bath.

Due to the initial adverse results, we used heterogeneous catalysts such as silica gel, alumina, or NaF/alumina as in the work of Saleh *et al.*^14^ where potassium persulfate (K_2_S_2_O_8_) was added to favor the oxidation step (Scheme 1a above). The catalytic effect of these solids is based on their diverse acidity (silica < Al_2_O_3_ < NaF–Al_2_O_3_)^41,42^ or maybe the NaF–Al_2_O_3_ basicity to *NH*-azoles for the fluoride anion.^43^ Ester 4c was obtained in poor yields using these solid catalysts (Table 1, entries 5 to 7), but with NaF–Al_2_O_3_, the yields can be increased using a higher F^−^ concentration in the solid or varying the mixture amount;^44^ however, the reaction time is crucial for the process (Table 1, entries 7 to 11). These results established that a yield greater than 60% was achieved with catalytic amounts of NaF–Al_2_O_3_ (Table 1, entry 11). The best yield was found when the reaction was heated in fusion using a sand bath at 180 °C for 10 min. In the absence of K_2_S_2_O_8_ under the optimized conditions, the yield was reduced to 43%, confirming the importance of this oxidizing agent (Table 1, entry 12 *vs.* 13).

**Scheme 5:**
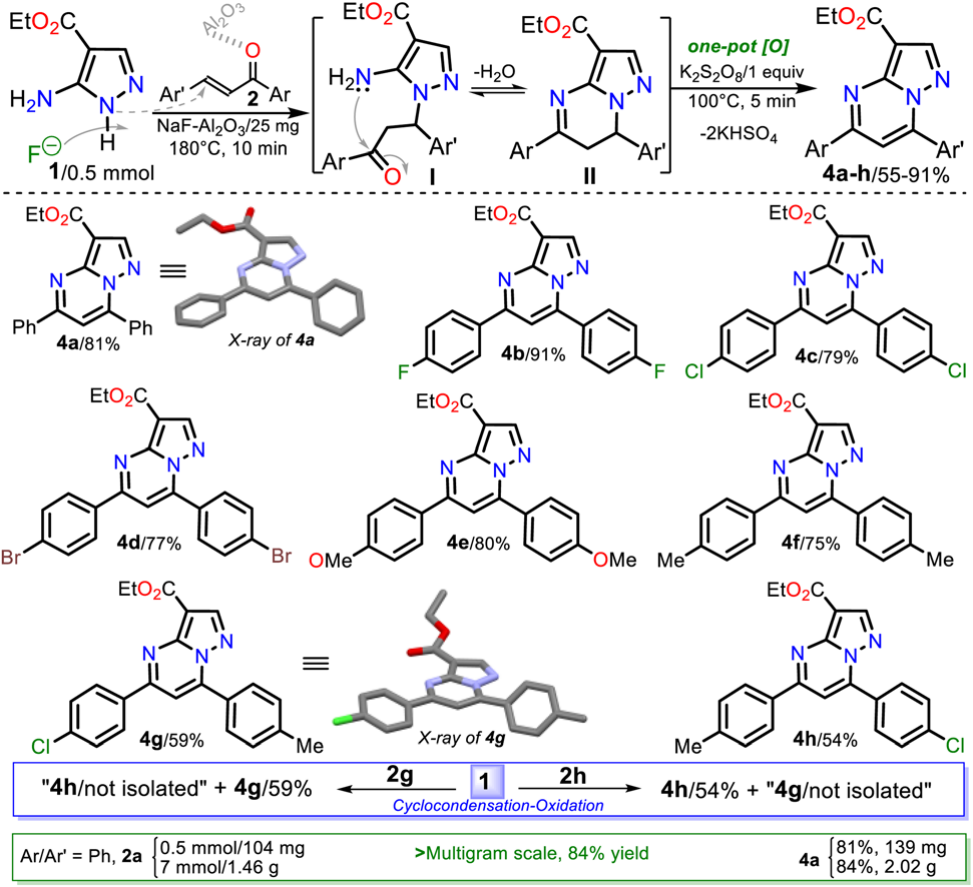
Synthesis of PPs 4a–h. A plausible formation way of 4a–h is shown.

Next, the reaction scope using various chalcones and the optimized conditions was examined. The reaction of an equimolar mixture of 1 with 2a–h (0.5 mmol) in the presence of NaF–Al_2_O_3_ (3 : 5 w/w, 25 mg) under heating at 180 °C for 10 min and then adding K_2_S_2_O_8_ (1 equiv.) to heat for another 5 min at 100 °C afforded the novel family of ethyl-5,7-diarylpyrazolo[1,5-*a*]pyrimidine-3-carboxylates 4a–h in high yields. Notably, substrates 2a–f have the same aryl group, which differs in 2g–h; this feature allows us to better establish the reaction regioselectivity in the initial step to form PPs 4a–h (Scheme 5).

Almost no loss of efficiency was observed in the synthesis of 4a–h with the chalcones tested, evidencing that the electronic demands of the substituents had little influence on the reactivity beyond the possible decomposition or evaporation of reagents under the established reaction conditions. However, the lowest yields were obtained using chalcones 2g–h, possibly due to the formation of 4h (using 2g) and 4g (using 2h), which are regioisomers of esters 4g and 4h, respectively (Scheme 5, blue rectangle); in any case, the high regioselectivity of reactions was demonstrated. Due to ester 4a being the key precursor for synthesizing the final products in this work (*i.e.*, Reversan and analogue amides), we carried out its synthesis on a scale of 2 g (Schemes 5, green rectangle). In this case, increasing the scale also resulted in a slight increase in the yields of 4a (from 81 to 84%).

**Fig. 2:**
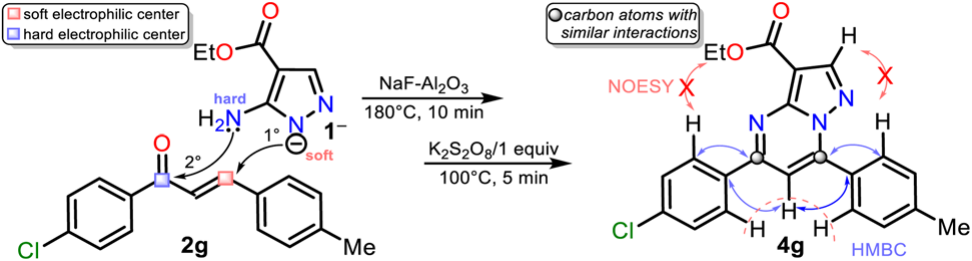
Synthesis of ethyl PPs 4a–h. A plausible formation way of 4a–h is shown.

Although there are reports for preparing PPs *via* the reaction of *NH*-5-aminopyrazoles with chalcones,^14–17^ a reasonable reaction route to yield products 4a–h under the optimized conditions in this work was established (Scheme 5 at the top). The reaction begins with an *aza*-Michael addition of the pyrrolic-like nitrogen atom in 1 (N1) to the Cβ of 2a–f, leading to intermediate I through a typical and dominant soft–soft interaction (Fig. 2).^11,45,46^ This attack is probably favored by fluoride ions (F^−^) in the catalyst, having a verified ability to remove the hydrogen atom from *NH*-azoles;^43^ likewise, the enone increases its electrophilicity when the carbonyl group interacts with alumina. Then, the cyclcondensation of I with the loss of a water molecule occurs to afford the dihydro derivative II (NH_2_/hard → C=O/hard), which is oxidized to the product 4a–h with K_2_S_2_O_8_ (1 equiv.) that favors the removal of hydrogen by converting to potassium bisulfate (KHSO_4_).^14^

Gratifyingly, structures of compounds 4a and 4g were solved by single-crystal X-ray diffraction analysis (see ESI†† for details). These results allowed us to verify the reaction course since substrate 2g, which leads to 4g, has two different aryl groups. It was impossible to establish the regioselectivity of the cyclocondensation reaction only using NMR analysis such as NOESY or HMBC experiments (Fig. 2).

It is important to mention what was cited in the introduction section: only four articles on obtaining 5,7-diarylpyrazolo[1,5-*a*]pyrimidines 4 starting from aminoester 1 have been published.^20–23^ In fact, only one of these works is comparable with synthesizing the eight PPs 4a–h because chalcones were used as substrates,^22^ and only compounds 4a and 4f have been reported in the literature. However, the method developed in this research presented better results than the cited works regarding process efficiency, synthetic versatility, and substrate scope (*e.g.*, chalcones or enones are easily accessible both synthetically and cheaply) and/or yields (see Scheme 1 above).^20–23^

### Synthesis of Reversan and analogues 5a–h

Once PPs 4a–h were obtained, we selected compound 4a to develop a simple and green method to generate Reversan and its structural analogues 5a–h by the direct amidation reaction of this ester with primary alkylamines 6a–h. In the amides synthesis on PPs, the respective carboxylic acid is used as a precursor; however, due to the possibility of the direct amidation of ester 4a, we tried to synthesize the *N*-butycarboxamide 5b with *n*-butylamine (2b) as a model reagent that allows us to optimize the conditions for this transformation, saving the hydrolysis step, although this reaction type is minorly used. Thus, considering our interest in MW-mediated reactions^40^ and the results of the NaF/alumina-catalyzed synthesis of 4a–g, we decided to start the study using similar conditions to those described by Ojeda-Porras *et al.*;^33^ they achieved the amidation of carboxylic acids with amines by irradiating an equimolar mixture (1.5 mmol) of reagents supported on silica gel (1.0 g) with MW for 4 cycles of 20 min at 130 °C (see Scheme 2a above). However, our results were not encouraging even after increasing the time and temperature (Table 2, entries 1 to 3).

**Table 2:** Optimization of the synthesis of *N*-butycarboxamide 4b^a^

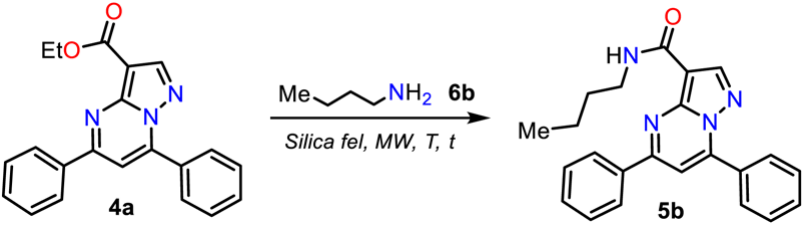				
Entry	6b (mmol)	*T* (°C)	Time *t* (min)	Yield (%)
1	0.15	130	30	NR
2	0.15	130	60	NR
3	0.15	160	30	NR
4	0.30	130	30	NR
5	0.30	160	30	Traces
6	0.30	180	30	Traces
7	0.30	180	60	35
8	0.45	160	30	22
9	0.45	180	30	73
10	0.45	180	60	65

a Reactions conditions: 4a, 6b, and 0.1 g silica gel in a 10 mL sealed tube under MW.

Because 6b is a volatile amine, we decided to double its proportion (from 0.15 to 0.30 mmol), wanting this would favor the reaction; however, it was only possible to observe the forming 5b in moderate yield after 1 hour of reaction at 180 °C (Table 2, entries 4 to 7). Next, we tripled the proportion of 6b (0.45 mmol), allowing us to form 5b in good yields, but the reaction is not better at less than 180 °C or more than 30 min (Table 2, entries 8 to 10).

**Scheme 6:**
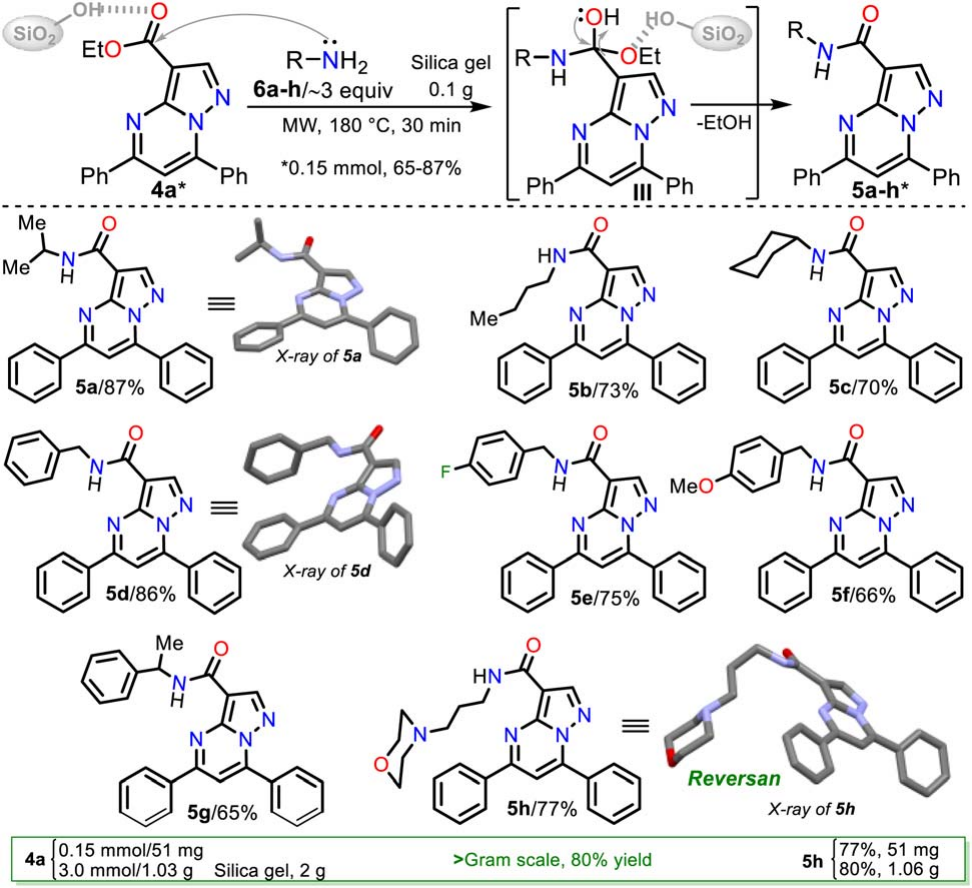
Synthesis of Reversan (5h) and its structural analogues 5a–g.

Consequently, the optimal reaction conditions to obtain 5b use ∼3 equivalents of starting amine 6b and 0.1 g silica gel at 180 °C for 30 min under microwave irradiation (Table 2, entry 9); we employed these conditions to examine the amidation reaction scope with various commercial primary alkylamines 6a–h. Fortunately, the MRP1 inhibitor, Reversan (5h), was obtained by the reaction of 4h with 3-morpholinopropanamine (6h); likewise, using alkylamines 6a–g, the Reversan structural analogues 5a–g were obtained, and all the synthesized amides were obtained in high yields. Moreover, crystals of suitable size and quality for single-crystal X-ray diffraction analysis of products 5a (*N*-iPr), 5d (*N*-Bn), and 5h (Reversan) were obtained by their recrystallization from methanol/ethyl acetate (1 : 3) using the slow evaporation method (Scheme 6, see ESI† for details).

**Scheme 7:**
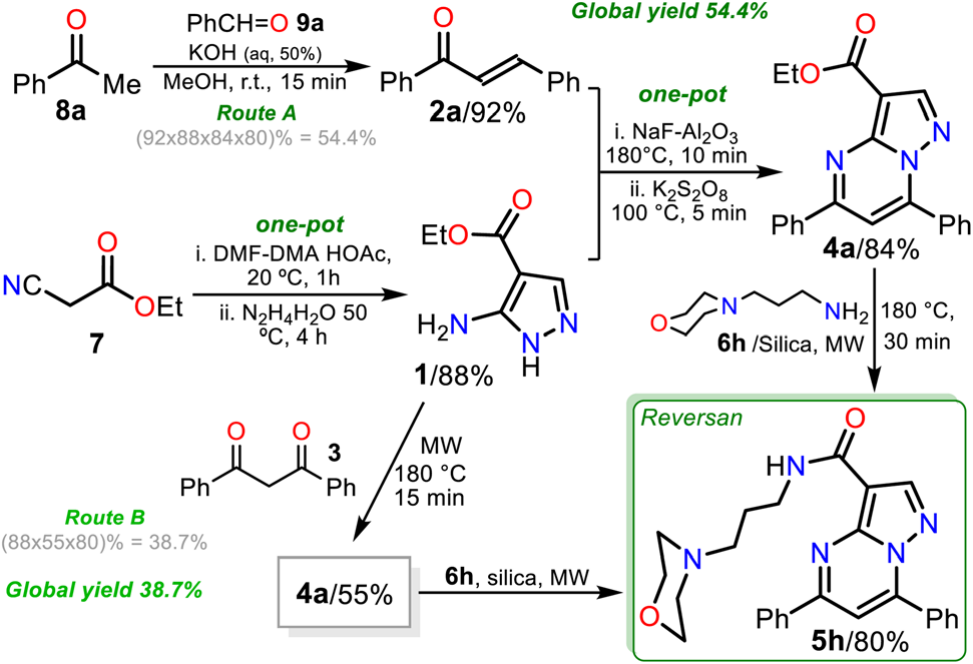
Synthesis of Reversan (5h) by routes A (is gram scale) and B.

Remarkable, silica gel is a heterogeneous catalyst serving challenging reactions that an acid medium could favor, such as the amidation reaction of esters studied in this work (see Scheme 6, at the top). In addition, due to the biological relevance and the high price of Reversan (5h), we obtained it on a one gram scale in high yield (80%) using 3.0 mmol ester 4a and 9 mmol 6h (Scheme 6, data in rectangle). Thus, this synthesis is a valuable input to the scientists investigating drug discovery with biological, pharmacological, or medicinal applications because we used cheap substances (*i.e.*, 3-morpholinopropanamine (6h), ethyl acetoacetate (7), acetophenone (8a), benzaldehyde (9a), DMF-DMA, acetic acid, hydrazine, KOH, NaF, K_2_S_2_O_8_, alumina, and silica gel) through simple, highly efficient, and even scalable synthetic methodologies (Scheme 7).

Finally, pyrazolo[1,5-*a*]pyrimidine ester 4a was also obtained by the reaction of dibenzoylmethane (3)^23^ with pyrazole aminoester 1 in microwaves, allowing us to develop a second synthesis of Reversan (route B) to compare with the first synthesis through enone 2a (route A). In route A, an overall yield of 54.4% was obtained in four reaction steps, while route B proceeded in three reaction steps with an overall yield of 38.7% using substrate 3, which is relatively expensive and more difficult to prepare than 2a (Scheme 7).^23^ Consequently, route A is better than route B because the best global yield is obtained, and with compounds 4b–h, greater structural diversity can be obtained for further studies related to Reversan and its structural analogues.

As a final comment regarding the results of this investigation, it should be noted that only one (Reversan 5h) of the eight amides synthesized 4a–h has been reported in the literature, and as cited in the introduction, a synthetic route for Reversan, which documents all the syntactic and characterization details, is yet to be reported.

## Conclusion

In summary, Reversan (5h) and its structural analogues (amides 5a–g) were synthesized by the silica-mediated direct amidation reaction between ethyl-5,7-diphenylpyrazolo[1,5-*a*]pyrimidine-3-carboxylate (4a) and primary amines 6a–h. In addition, a family of ethyl-5,7-diarylpyrazolo[1,5-*a*]pyrimidine-3-carboxylates 4a–h was obtained when aminoester 1 was cyclocondensed with chalcones 2a–h using the NaF-alumina catalyst and as the final step, a Na_2_S_2_O_8_-mediated oxidation reaction. All products were obtained in high yields *via* simple, efficient, and scalable methodologies using cheap reagents, heterogeneous catalysts such as silica gel or NaF-alumina, and solvent-free reactions in fusion or microwaves. Remarkably, the two relevant reaction types for this work (cyclocondensations of chalcones with 5-aminopyrazoles bearing an EWG and esters amidation) are protocols rarely used. In addition, the obtained compounds were characterized by spectroscopic analysis, and the structures of some intermediates and products (4a, 4g, 5a, 5d, and 5h) were confirmed by single-crystal X-ray diffraction analysis. Therefore, we developed synthetic methods that address some of the key points associated with green chemistry principles employing easily accessible substances.

## Experimental section

### Reagents and materials

The reagents and substances used in this investigation were purchased from commercial sources and used without further purification; these were weighed and handled in the air at room temperature. The reaction was monitored by thin-layer chromatography (TLC), visualized by a UV lamp (254 or 365 nm), and flash chromatography was performed on silica gel (230–400 mesh). Reactions under microwave irradiation were carried out in a sealed reaction vessel (10.0 mL, max pressure = 300 psi) containing a Teflon-coated stir bar (obtained from CEM) and were performed in a CEM Discover SP-focused MW (*ν* = 2.45 GHz) reactor equipped with a built-in pressure measurement sensor and a vertically-focused IR temperature sensor. Controlled temperature, power, and time settings were used. Substrates based on *NH*-5-aminopyrazoles 1 and 1′ and chalcone derivatives 2a–h were prepared by known procedures and developed by us (see ESI† for details of the synthesis of these substrates).

The NMR spectra for this work were recorded at 400 MHz (^1^H) and 101 MHz (^13^C) at 298 K, and the data were recorded in CDCl_3_ (7.26/77.05 ppm) or DMSO (2.50/39.5 ppm) using the residual nondeuterated signal for ^1^H and the deuterated solvent signal for ^13^C NMR as internal standards. Chemical shifts (*δ*) are given in parts per million (and coupling constants (*J*) in Hertz (Hz). The multiplicity abbreviations involve s = singlet, d = doublet, t = triplet, q = quartet, and m = multiplet (see copies of NMR spectra in Fig. S3–S31 of ESI†). Melting points were determined using a capillary melting point apparatus, and the data were uncorrected. High-resolution mass spectra (HRMS) were recorded using a Q-TOF spectrometer by electrospray ionization (ESI) (see the HRMS analysis in Fig. S32–S47 of ESI†). The X-ray intensity data were measured at 25(2) °C using CuKα radiation (*λ* = 1.54184 Å), by ω scans in an Agilent SuperNova, Dual, Cu at Zero, Atlas four-circle diffractometer equipped with a CCD plate detector (see ESI† for more crystallographic details).

### Synthesis and characterization

#### Synthesis of 5,7-diarylpyrazolo[1,5-*a*]pyrimidines 4a′–c′

A mixture of 3-methyl-1*H*-pyrazol-5-amine (1′, 0.6 mmol, 58 mg), the respective chalcone derivative 2 (0.5 mmol), KOH (0.05 mmol 3 mg), and DMF (2.5 mL) was heated at 110 °C for 20 min under constant stirring. The mixture was then allowed to cool to room temperature, water (10 mL) was added, and the aqueous mixture was extracted with ethyl acetate (3 × 10 mL). The organic extract was dried over anhydrous Na_2_SO_4_, and the solvent was removed under reduced pressure. The residue was purified by flash chromatography on silica gel (eluent: *n*-pentane/AcOEt 4 : 1 v/v) to afford products 4a′–d′ (ref. 16).

##### 2-Methyl-5,7-diphenylpyrazolo[1,5-*a*]pyrimidine (4a′)

By the general procedure with chalcone (2a, 104 mg, 0.5 mmol), 4a′ was obtained as a paled-yellow solid (50 mg, 35%, amorphous). Mp: 118–119 °C (Lit.^8^ 117–118 °C). Mp 117–119 °C. ^1^H NMR (400 MHz, CDCl_3_): *δ* = 2.55 (s, 3H), 6.59 (s, 1H), 7.24 (s, 1H), 7.45–7.58 (m, 6H), 8.05–8.14 (m, 4H) ppm. ^13^C NMR (100 MHz, CDCl_3_): *δ* = 14.9 (CH_3_), 96.4 (CH), 104.3 (CH), 127.1 (CH), 128.6 (CH), 128.8 (CH), 129.2 (CH), 130.0 (CH), 130.8 (CH), 131.6 (C), 137.7 (C), 146.1 (C), 150.6 (C), 155.4 (C), 155.7 (C) ppm. These NMR data matched previously reported data.^8^

##### 5,7-Bis(4-chlorophenyl)-2-methylpyrazolo[1,5-*a*]pyrimidine (4b′)

By the general procedure with the dichlorochalcone 2c (139 mg, 0.5 mmol), 4b′ was obtained as a light greenish solid (55 mg, 31%, amorphous). Mp: 180–182 °C (Lit.^8^ 179–181 °C). ^1^H NMR (400 MHz, CDCl_3_): *δ* = 2.53 (s, 3H), 6.57 (s, 1H), 7.18 (s, 1H), 7.48 (d, *J* = 8.7 Hz, 2H), 7.55 (*d*, *J* = 8.7 Hz, 2H), 8.05 (*d*, *J* = 8.7 Hz, 4H) ppm. ^13^C NMR (100 MHz, CDCl_3_): *δ* = 14.9 (CH_3_), 96.8 (CH), 103.8 (CH), 128.5 (CH), 129.1 (CH), 129.2 (CH), 129.9 (C), 130.7 (CH), 136.0 (C), 136.5 (C), 137.2 (C), 145.2 (C), 150.5 (C), 154.5 (C), 155.8 (C) ppm. These NMR data matched previously reported data.^8^

##### 2-Methyl-5,7-di-*p*-tolylpyrazolo[1,5-*a*]pyrimidine (4c′)

By the general procedure with dimethylchalcone 2f (118 mg, 0.5 mmol), 4c was obtained as a yellow solid (56 mg, 36%, amorphous). Mp: 150–151 °C (amorphous) (Lit.^16^ 155–157 °C). ^1^H NMR (400 MHz, CDCl_3_): *δ* = 2.43 (s, 3H), 2.46 (s, 3H), 2.52 (s, 3H), 6.54 (s, 1H), 7.21 (s, 1H), 7.31 (d, *J* = 8.0 Hz, 2H), 7.37 (*d*, *J* = 8.1 Hz, 2H), 7.98–8.01 (dd, *J* = 8.1 Hz, 4H) ppm. ^13^C NMR (100 MHz, CDCl_3_): *δ* = 15.0 (CH_3_), 21.4 (CH_3_), 21.6 (CH_3_), 96.2 (CH), 104.0 (CH), 127.1 (CH), 128.9 (C), 129.2 (CH), 129.4 (CH), 139.6 (CH), 135.0 (C), 140.3 (C), 141.2 (C), 146.2 (C), 150.7 (C), 155.2 (C), 155.8 (C) ppm. These NMR data matched previously reported data.^16^

#### Synthesis of 3-carboethoxypyrazolo[1,5-*a*]pyrimidines 4a–h

Equimolar amounts of ethyl-5-amino-1*H*-pyrazole-4-carboxylate (1, 0.5 mmol, 78 mg) and the respective enone 2a–h were thoroughly mixed at room temperature, together with 25 mg of NaF–Al_2_O_3_ (3 : 5, w/w), into a 10 mL sealable (Teflon screw cap) tubular reaction vessel. The mixture was heated in fusion over the solid support and catalyst at 180 °C for 10 min using a sand bath. Next, 1 equiv. K_2_S_2_O_8_ (135 mg) was added, and the mixture continued heating for another 5 min at 100 °C. The resulting reaction mixture was then cooled to room temperature and extracted with ethyl acetate (3 × 10 mL). The extract was dried over anhydrous Na_2_SO_4_, the solvent was removed, and the crude product was purified by flash chromatography on silica gel (eluent: CH_2_Cl_2_) to afford the desired products 4a–h in good yields. The recrystallization of 4a and 4g from methanol/AcOEt (1 : 3) afforded crystalline colorless prisms suitable for X-ray diffraction analysis.

##### 3-Carboethoxy-5,7-diphenylpyrazolo[1,5-*a*]pyrimidine (4a)

By the general procedure with chalcone (2a, 104 mg, 0.5 mmol), 4a was obtained as a yellow solid (139 mg, 81%, amorphous). For the multigram scale, 7 mmol of reagents (*i.e.*, 1/1.09 g, 2a/1.46 g, NF–Al_2_O_3_/0.35 g, and K_2_S_2_O_8_/1.89 g) and a 35 mL tubular reaction vessel were used. This compound was also obtained (95 mg, 55%) using an equimolar mixture with dibenzoylmethane (3, 0.5 mmol, 112 mg) as the 1,3-bis(electrophilic) substrate, which was irradiated under microwaves conditions at 180 °C for 15 min. Mp: 101–103 °C (Lit.^20^ 79–81 °C). ^1^H NMR (400 MHz, CDCl_3_): *δ* = 1.48 (t, *J* = 7.1 Hz, 3H), 4.46 (q, *J* = 7.1 Hz, 2H) 7.49–7.53 (m, 4H) 7.58–7.62 (m, 3H), 7.99–8.04 (m, 2H), 8.24–8.28 (m, 2H), 8.60 (s, 1H) ppm. ^13^C NMR (100 MHz, CDCl_3_): *δ* = 14.5 (CH_3_), 60.2 (CH_2_), 103.0 (C), 106.4 (CH), 127.6 (CH), 128.8 (CH), 128.9 (CH), 129.4 (CH), 130.7 (CH), 131.1 (CH), 131.4 (C), 136.5 (C), 147.7 (CH), 147.8 (C), 148.7 (C), 159.0 (C), 162.8 (C) ppm. HRMS (ESI) *m*/*z*: [M + H]^+^ calcd for C_21_H_18_N_3_O_2_^+^ 344.1394; found 344.1400. These data matched previously reported data using another method.^20^

##### 5,7-Bis(4-fluorophenyl)-3-carboethoxypyrazolo[1,5-*a*]pyrimidine (4b)

By the general procedure with difluorochalcone 2b (122 mg, 0.5 mmol), 4b was obtained as a yellow solid (173 mg, 91%, amorphous). Mp: 123–124 °C. ^1^H NMR (400 MHz, CDCl_3_): *δ* = 1.47 (t, *J* = 7.1 Hz, 3H), 4.45 (q, *J* = 7.1 Hz, 2H), 7.19 (t, *J* = 8.5 Hz, 2H), 7.29 (t, *J* = 8.3 Hz, 2H), 7.44 (s, 1H), 8.07 (dd, *J* = 8.1 Hz, 2H), 8.26 (dd, *J* = 8.2 Hz, 2H), 8.57 (s, 1H) ppm. ^13^C NMR (100 MHz, CDCl_3_): 14.5 (CH_3_), 60.3 (CH_2_), 103.0 (C), 105.8 (CH), 115.9/116.2 (CH, d, *J* = 21.7 Hz), 115.9/116.1 (CH, d, *J* = 21.7 Hz), 126.6 (C, d, *J* = 3.7 Hz), 129.8 (CH, d, *J* = 8.8 Hz), 131.8 (CH, d, *J* = 9.0 Hz), 132.6 (C, *J* = 3.0 Hz), 146.8 (C), 147.8 (CH), 148.8 (C), 157.8 (C), 162.7 (C), 163.2/165.7 (CF, d, *J* = 253.1 Hz), 163.5/166.0 (CF, d, *J* = 252.4 Hz) ppm. HRMS (ESI) *m*/*z*: [M + H]^+^ calcd for C_21_H_16_F_2_N_3_O_2_^+^ 380.1205; found 380.1206.

##### 5,7-Bis(4-chlorophenyl)-3-carboethoxypyrazolo[1,5-*a*]pyrimidine (4c)

By the general procedure with dichlorochalcone 2c (139 mg, 0.5 mmol), 4c was obtained as a yellow solid (163 mg, 79%, amorphous). Mp: 175–176 °C. ^1^H NMR (400 MHz, CDCl_3_): *δ* = 1.46 (t, *J* = 7.1 Hz, 3H), 4.45 (q, *J* = 7.2 Hz, 2H), 7.45 (s, 1H), 7.48 (d, *J* = 8.6 Hz, 2H), 7.58 (d, *J* = 8.6 Hz, 2H), 8.00 (d, *J* = 8.6 Hz, 2H), 8.19 (d, *J* = 8.7 Hz, 2H), 8.57 (s, 1H) ppm. ^13^C NMR (100 MHz, CDCl_3_): *δ* = 14.5 (CH_3_), 60.4 (CH_2_), 103.3 (C), 105.8 (CH), 128.8 (C), 128.9 (CH), 129.2 (CH), 129.3 (CH), 130.8 (CH), 134.8 (C), 137.6 (C), 137.8 (C), 146.8 (C), 147.9 (CH), 148.8 (C), 157.6 (C), 162.6 (C) ppm. HRMS (ESI) *m*/*z*: [M + H]^+^ calcd for C_21_H_16_^35^Cl_2_N_3_O_2_^+^ 412.0614; found 412.0615.

##### 5,7-Bis(4-bromophenyl)-3-carboethoxypyrazolo[1,5-*a*]pyrimidine (4d)

By the general procedure with dibromochalcone 2d (183 mg, 0.5 mmol), 4d was obtained as a yellow solid (193 mg, 77%, amorphous). Mp: 123–124 °C. ^1^H NMR (400 MHz, CDCl_3_): *δ* = 1.47 (t, *J* = 7.1 Hz, 2H), 4.46 (q, *J* = 7.2 Hz, 2H), 7.47 (s, 1H), 7.67 (d, *J* = 8.5 Hz, 2H), 7.75 (d, *J* = 8.6 Hz, 2H), 7.93 (d, *J* = 8.5 Hz, 2H), 8.14 (d, *J* = 8.6 Hz, 2H), 8.59 (s, 1H) ppm. ^13^C NMR (100 MHz, CDCl_3_): *δ* = 14.6 (CH_3_), 60.4 (CH_2_), 103.4 (C), 105.8 (CH), 126.2 (C), 126.3 (C), 129.2 (CH), 129.4 (C), 131.0 (CH) 132.2 (CH), 132.3 (CH), 135.3 (C), 147.0 (C), 148.0 (CH), 148.8 (C), 157.8 (C), 162.7 (C) ppm. HRMS (ESI) *m*/*z*: [M + H]^+^ calcd for C_21_H_16_^79^Br_2_N_3_O_2_^+^ 499.9604; found 499.9606.

##### 5,7-Bis(4-metoxyphenyl)-3-carboethoxypyrazolo[1,5-*a*]pyrimidine (4e)

By the general procedure with dimethoxychalcone 2e (134 mg, 0.5 mmol), 4e was obtained as a yellow solid (161 mg, 80%, amorphous). Mp: 175–176 °C. ^1^H NMR (400 MHz, CDCl_3_): *δ* = 1.47 (t, *J* = 7.2 Hz, 3H), 3.88 (s, 3H), 3.90 (s, 3H), 4.45 (q, *J* = 7.2 Hz, 2H), 7.01 (d, *J* = 8.7 Hz, 2H), 7.09 (d, *J* = 8.8 Hz, 2H), 7.42 (s, 1H), 8.03 (d, *J* = 8.7 Hz, 2H), 8.23 (d, *J* = 8.7 Hz, 2H), 8.55 (s, 1H) ppm. ^13^C NMR (100 MHz, CDCl_3_): *δ* = 14.6 (CH_3_), 55.4 (CH_3_), 55.5 (CH_3_), 60.1 (CH_2_), 102.3 (C), 105.1 (CH), 114.2 (CH), 114.3 (CH), 122.9 (C), 129.1 (C), 129.3 (CH), 131.2 (CH), 147.4 (C), 147.5 (CH), 149.1 (C), 158.5 (C), 161.9 (C), 162.1 (C), 163.0 (C) ppm. HRMS (ESI): [M + H]^+^ calcd for C_23_H_22_N_3_O_4_^+^ 404.1605; found 404.1611.

##### 3-Carboethoxy-5,7-di-*p*-tolylpyrazolo[1,5-*a*]pyrimidine (4f)

By the general procedure with dimethyl-chalcone 2f (118 mg, 0.5 mmol), 4f was obtained as a yellow solid (139 mg, 75%, amorphous). Mp: 130–132 °C (Lit.^20^ 129–131 °C). ^1^H NMR (400 MHz, CDCl_3_): *δ* = 1.48 (t, *J* = 7.2 Hz, 3H), 2.45 (s, 3H), 2.48 (s, 3H), 4.46 (q, *J* = 7.2 Hz, 2H), 7.34 (d, *J* = 8.2 Hz, 2H), 7.41 (d, *J* = 8.2 Hz, 2H), 7.49 (s, 1H), 7.93 (d, *J* = 8.1 Hz, 2H), 8.18 (d, *J* = 8.2 Hz, 2H), 8.57 (s, 1H) ppm. ^13^C NMR (100 MHz, CDCl_3_): *δ* = 14.6 (CH_3_), 21.5 (CH_3_), 21.6 (CH_3_), 60.2 (CH_2_), 102.7 (C), 105.9 (CH), 127.6 (CH), 127.9 (C), 129.4 (CH), 129.5 (CH), 129.8 (CH), 133.9 (C), 141.7 (C), 142.0 (C), 147.7 (CH), 147.9 (C), 149.0 (C), 159.0 (C), 163.0 (C) ppm. HRMS (ESI): [M + H]^+^ calcd for C_23_H_22_N_3_O_2_^+^ 372.1707; found 372.1708. These data matched previously reported data.^20^

##### 3-Carboethoxy-5-(4-chlorophenyl)-7-(*p*-tolyl)pyrazolo[1,5-*a*]pyrimidine (4g)

This ester was obtained, following the general procedure with (*E*)-1-(4-chlorophenyl)-3-(*p*-tolyl)propen-1-one (2g, 128 mg, 0.5 mmol), as a yellow solid (116 mg, 59%, amorphous). Mp: 163–164 °C. ^1^H NMR (400 MHz, CDCl_3_): *δ* = 1.47 (t, *J* = 7.2 Hz, 3H), 2.48 (s, 3H), 4.46 (q, *J* = 7.0 Hz, 2H), 7.42 (d, *J* = 8.3 Hz, 2H), 7.47 (s, 1H), 7.51 (d, *J* = 8.7 Hz, 2H), 7.94 (d, *J* = 8.2 Hz, 2H), 8.23 (d, *J* = 8.7 Hz, 2H), 8.59 (s, 1H) ppm. ^13^C NMR (100 MHz, CDCl_3_): *δ* = 14.5 (CH_3_), 21.6 (CH_3_), 60.3 (CH_2_), 103.0 (C), 105.7 (CH), 127.6 (C), 128.9 (CH), 129.2 (CH), 129.4 (CH), 129.5 (CH), 135.1 (C), 137.4 (C), 142.2 (C), 147.8 (CH), 148.2 (C), 148.9 (C), 157.6 (C), 162.8 (C) ppm. HRMS (ESI): [M + H]^+^ calcd for C_22_H_18_ClN_3_O^+^ 392.1160; found 371. 392.1179.

##### 3-Carboethoxy-7-(4-chlorophenyl)-5-(*p*-tolyl)pyrazolo[1,5-*a*]pyrimidine (4h)

This ester was obtained, following the general process with (*E*)-3-(4-chlorophenyl)-1-(*p*-tolyl)propen-1-one (2h, 128 mg, 0.5 mmol), as a yellow solid (106 mg, 54%, amorphous). Mp: 150–151 °C. ^1^H NMR (400 MHz, CDCl_3_): *δ* = 1.48 (t, *J* = 7.1 Hz, 3H), 2.45 (s, 3H), 4.46 (q, *J* = 7.1 Hz, 2H), 7.35 (d, *J* = 8.3 Hz, 2H), 7.49 (s, 1H), 7.59 (d, *J* = 7.0 Hz, 2H), 8.00 (d, *J* = 8.6 Hz, 2H), 8.18 (d, *J* = 7.0 Hz, 2H), 8.57 (s, 1H) ppm.^13^C NMR (100 MHz, CDCl_3_): *δ* = 14.6 (CH_3_), 21.6 (CH_3_), 60.3 (CH_2_), 103.0 (C), 106.0 (CH), 127.6 (CH), 129.2 (CH), 129.8 (CH), 130.9 (CH), 133.7 (C), 137.7 (C), 141.9 (C), 146.6 (C), 147.8 (CH), 149.0 (C), 159.1 (C), 162.9 (C) ppm. HRMS (ESI^+^): [M + H]^+^ calcd for C_22_H_18_ClN_3_O^+^ 392.1160; found 371. 392.1163.

#### Synthesis of *N*-alkyl-3-carbamoylpyrazolo[1,5-*a*]pyrimidines 5a–h

A mixture of 3-carboethoxy-5,7-diphenylpyrazolo[1,5-*a*]pyrimidine (4a, 51 mg, 0.15 mmol) and an excess of primary alkylamine 6a–h (∼3 equiv.) was dissolved in ethyl ether (1 mL); then, silica gel (230–400 mesh, 100 mg) was added, thoroughly mixing all components at room temperature. After removing the ether, the solid residue was subjected to microwave irradiation at 180 °C (200 W, monitored by an IR temperature sensor) and maintained at this temperature for 30 min in a sealed MW tube of 10 mL. The resulting mixture was cooled to room temperature by airflow, ethyl acetate was added (3.0 mL), and sonicated for 20 min; this mixture was filtered, the solid residue was washed with ethyl acetate (3.0 mL), and the organic phase was washed with a saturated solution of NaHCO_3_ and HCl 10%. The organic extract was dried over anhydrous Na_2_SO_4_, filtered, and the solvent was removed under reduced pressure. The residue was purified by flash chromatography on silica gel (eluent: CH_2_Cl_2_/MeOH 30 : 1 v/v) to afford products 5a–h in good yields. The recrystallization of amides 5a, 5d, and 5h from methanol/ethyl acetate (1 : 3) afforded crystalline colorless prisms suitable for X-ray diffraction analysis.

##### *N*-Isopropyl-5,7-diphenylpyrazolo[1,5-*a*]pyrimidine-3-carboxamide (5a)

By the general procedure with isopropylamine (6a, 30 mg, 0.50 mmol), 5a was obtained as a pale greenish solid (46.5 mg, 87%, amorphous). Mp: 203–204 °C. ^1^H NMR (400 MHz, CDCl_3_): *δ* = 1.38 (d, *J* = 6.5 Hz, 6H), 4.38 (m, 1H), 7.48 (s, 1H), 7.56–7.64 (m, 6H), 8.04–8.17 (m, 4H), 8.25 (d, *J* = 7.5 Hz, NH), 8.71 (s, 1H) ppm. ^13^C NMR (100 MHz, CDCl_3_): *δ* = 23.2 (CH_3_), 40.9 (CH), 105.7 (CH), 106.1 (C), 127.2 (CH), 128.8 (CH), 129.2 (CH), 129.4 (CH), 130.5 (C), 131.2 (CH), 131.6 (CH), 136.4 (C), 146.8 (C), 146.9 (CH), 148.2 (C), 157.6 (C), 161.6 (C) ppm. HRMS (ESI^+^): [M + H]^+^ calcd for C_23_H_21_N_4_O^+^ 357.1710; found 357.1709.

##### *N*-Butyl-5,7-diphenylpyrazolo[1,5-*a*]pyrimidine-3-carboxamide (5b)

By the general procedure with *n*-butylamine (6b, 33 mg, 0.45 mmol), 5b was obtained as a brown solid (41 mg, 73%, amorphous). Mp: 97–99 °C. ^1^H NMR (400 MHz, CDCl_3_): *δ* = 1.03 (t, *J* = 7.3 Hz, 3H), 1.56 (m, 2H), 1.72 (m, 2H), 3.59 (q, *J* = 6.8 Hz, 2H), 7.49 (s, 1H), 7.56–7.67 (m, 6H), 8.03–8.17 (m, 4H), 8.31 (t, *J* = 5.4 Hz, NH), 8.72 (s, 1H, H2) ppm. ^13^C NMR (100 MHz, CDCl_3_): *δ* = 13.9 (CH_3_), 20.3 (CH), 31.8 (CH_2_), 38.7 (CH_2_), 105.9 (C), 106.1 (CH), 127.4 (CH), 128.9 (CH), 129.2 (CH), 129.4 (CH), 130.5 (C), 131.2 (CH), 131.6 (CH), 136.5 (C), 146.8 (C), 147.0 (CH), 148.2 (C), 157.8 (C), 162.4 (C) ppm. HRMS (ESI^+^): [M + H]^+^ calcd for C_23_H_23_N_4_O^+^ 371.1866; found 371.1896.

##### *N*-Cyclohexyl-5,7-diphenylpyrazolo[1,5-*a*]pyrimidine-3-carboxamide (5c)

By the general procedure (200 °C, 30 min × 4) with cyclohexylamine (6c, 50 mg, 0.50 mmol), 5c was obtained as a yellow solid (42 mg, 70%, amorphous). Mp: 188–190 °C. ^1^H NMR (400 MHz, CDCl_3_): *δ* = 1.26–1.57 (m, 5H), 1.67 (m, 1H), 1.80 (m, 2H), 2.10 (m, 2H), 4.15 (m, 1H), 7.48 (s, 1H), 7.56–7.63 (m, 6H), 8.04–8.16 (m, 4H), 8.35 (d, *J* = 8.1 Hz, NH), 8.71 (s, 1H) ppm. ^13^C NMR (100 MHz, CDCl_3_): *δ* = 24.5 (CH_2_), 25.8 (CH_2_), 33.2 (CH_2_), 47.3 (CH), 105.9 (CH), 106.3 (C), 127.4 (CH), 128.9 (CH), 129.3 (CH), 129.5 (CH), 130.6 (C), 131.2 (CH), 131.6 (CH), 136.6 (C), 146.9 (C), 147.1 (CH), 148.3 (C), 157.7 (C), 161.5 (C). HRMS (ESI^+^): [M + H]^+^ calcd for C_25_H_25_N_4_O^+^ 397.2023; found 397.2031.

##### *N*-Benzyl-5,7-diphenylpyrazolo[1,5-*a*]pyrimidine-3-carboxamide (5d)

By the general procedure with benzylamine (6d, 50 mg, 0.47 mmol), 5d was obtained as a pale greenish solid (52.2 mg, 86%, amorphous). Mp: 208–209 °C. ^1^H NMR (400 MHz, CDCl_3_): *δ* = 4.77 (d, *J* = 5.4 Hz, 2H), 7.32–7.56 (m, 9H), 7.63 (m, 3H), 7.95 (d, *J* = 8.0 Hz, 2H), 8.07 (m, 2H), 8.66 (t, *J* = 5.4 Hz, 1H), 8.75 (s, 1H) ppm. ^13^C NMR (100 MHz, CDCl_3_): *δ* = 43.6 (CH_2_), 105.7 (C), 105.8 (CH), 127.3 (CH), 127.4 (CH), 127.9 (CH), 128.8 (CH), 128.9 (CH), 129.1 (CH), 129.4 (CH), 130.5 (C), 131.2 (CH), 131.6 (CH), 136.1 (C), 138.7 (C), 146.9 (CH), 147.1 (C), 148.3 (C), 157.7 (C), 162.3 (C) ppm. HRMS (ESI^+^): [M + H]^+^ calcd for C_26_H_21_N_4_O^+^ 405.1710; found 405.1710.

##### *N*-(4-Fluorobenzyl)-5,7-diphenylpyrazolo[1,5-*a*]pyrimidine-3-carboxamide (5e)

By the general procedure (30 min × 3) with 4-flurorobenzylamine (6e, 60 mg, 0.48 mmol), 5e was obtained as a yellow solid (47.5 mg, 75%, amorphous). Mp: 77–78 °C. ^1^H NMR (400 MHz, CDCl_3_): *δ* = 4.72 (d, *J* = 5.3 Hz, 2H), 7.09 (t, *J* = 8.7 Hz, 2H), 7.43–7.50 (m, 5H), 7.55 (t, *J* = 7.2 Hz, 1H), 7.62 (m, 3H), 7.94 (d, *J* = 8.0 Hz, 2H), 8.06 (m, 2H), 8.64 (t, *J* = 5.4 Hz, NH), 8.73 (s, 1H) ppm. ^13^C NMR (100 MHz, CDCl_3_): *δ* = 42.9 (CH_3_), 105.6 (C), 105.9 (CH), 115.5/115.7 (CH, d, *J* = 21.6 Hz), 127.3 (CH), 128.9 (CH), 129.2 (CH), 129.5 (CH), 129.6/129.7 (CH, *J* = 8.1 Hz), 130.4 (C), 131.4 (CH), 131.7 (CH), 134.6 (C, d, *J* = 3.3 Hz), 136.1 (C), 146.9 (CH), 147.1 (C), 148.4 (C), 157.8 (C), 163.4/161.0 (CF, d, *J* = 245.4 Hz), 162.3 (C) ppm. HRMS (ESI^+^): [M + H]^+^ calcd for C_26_H_20_N_4_O^+^ 423.1616; found 419.1615.

##### *N*-(4-Methoxybenzyl)-5,7-diphenylpyrazolo[1,5-*a*]pyrimidine-3-carboxamide (5f)

By the general procedure (30 min × 2) with 4-methoxybenzylamine (6f, 65 mg, 0.47 mmol), 5f was obtained as an orange solid (43 mg, 66%, amorphous). Mp: 171–172 °C. ^1^H NMR (400 MHz, CDCl_3_): *δ* = 3.84 (s, 3H), 4.68 (d, *J* = 5.1 Hz, 2H), 6.94 (d, *J* = 8.8 Hz, 2H), 7.38–7.48 (m, 4H), 7.52 (t, *J* = 7.2 Hz, 1H), 7.59–7.65 (m, 3H), 7.92 (d, *J* = 8.3 Hz, 2H), 8.03–8.07 (m, 2H), 8.60 (t, *J* = 5.0 Hz, 1H), 8.73 (s, 1H). ^13^C NMR (100 MHz, CDCl_3_): *δ* = 43.1 (CH_3_), 55.3 (CH_2_), 105.7 (CH), 105.8 (C), 114.1 (CH), 127.3 (CH), 128.9 (CH), 129.1 (CH), 129.3 (CH), 129.4 (CH), 130.4 (C), 130.8 (C), 131.3 (CH), 131.6 (CH), 136.0 (C), 146.9 (CH), 147.0 (C), 148.3 (C), 157.6 (C), 159.0 (C), 162.2 (C). HRMS (ESI^+^): [M + H]^+^ calcd for C_27_H_23_N_4_O^+^ 435.1816; found 435.1831.

##### 5,7-Diphenyl-*N*-(1-phenylethyl)pyrazolo[1,5-*a*]pyrimidine-3-carboxamide (5g)

By the general procedure with 1-phenyl-ethanamine (6g, 60 mg, 0.49 mmol), 5g was obtained as a yellow solid (40.8 mg, 65%, amorphous). Mp: 75–76 °C. ^1^H NMR (400 MHz, CDCl_3_): *δ* = 1.71 (d, *J* = 6.8 Hz, 3H), 5.40 (m, 1H), 7.33 (t, *J* = 7.2 Hz, 1H), 7.41 (t, *J* = 7.6 Hz, 2H) 7.48 (s, 1H), 7.50–763 (m, 8H), 7.99–8.08 (m, 4H), 8.71 (s, 1H), 8.75 (d, *J* = 7.5 Hz, NH) ppm. ^13^C NMR (100 MHz, CDCl_3_): *δ* = 22.7 (CH_3_), 49.0 (CH), 105.8 (CH), 105.9 (C), 126.45 (CH), 127.3 (CH), 127.4 (CH), 128.8 (CH), 128.9 (CH), 129.2 (CH), 129.4 (CH), 130.5 (C), 131.3 (CH), 131.7 (CH), 136.2 (C), 143.9 (C), 146.9 (C), 147.0 (C), 148.3 (C), 157.7 (C), 161.6 (C) ppm. HRMS (ESI^+^): [M + H]^+^ calcd for C_27_H_23_N_4_O^+^ 419.1866; found 419.1864.

##### *N*-(3-morpholinopropyl)-5,7-diphenylpyrazolo[1,5-*a*]pyrimidine-3-carboxamide (5h)

By the general procedure with 3-morpholino-propanamine (6h, 70 mg, 0.48 mmol), Reversan (5h) was obtained as a yellow solid (51 mg, 77%, amorphous). For the one gram scale, 3 mmol of 1a (1.03 g), 9 mmol of 2h (1.3 g), and a 35 mL tubular reaction vessel were used (1.06 g, 80%). Mp: 94–95 °C. ^1^H NMR (400 MHz, CDCl_3_): *δ* = 1.92 (m, *J* = 6.9 Hz, 2H), 2.46 (m, 4H), 2.53 (t, *J* = 7.3 Hz, 2H), 3.61–3.70 (m, 6H), 7.49 (s, 1H), 7.54–7.64 (m, 6H), 8.04–8.16 (m, 4H), 8.34 (t, *J* = 6.2 Hz, NH), 8.72 (s, 1H) ppm. ^13^C NMR (100 MHz, CDCl_3_): *δ* = 27.0 (CH_2_), 37.3 (CH_2_), 53.8 (CH_2_), 56.6 (CH_2_), 67.0 (CH_2_), 106.0 (C), 106.1 (CH), 127.5 (CH), 129.0 (CH), 129.3 (CH), 129.5 (CH), 130.5 (C), 131.4 (CH), 131.8 (CH), 136.6 (C), 147.0 (C), 147.1 (C2), 148.4 (C), 158.0 (C), 162.5 (C) ppm. HRMS (ESI^+^): [M + H]^+^ calcd for C_26_H_27_N_5_O_2_^+^ 442.2238; found 442.2253.

## Conflicts of interest

The authors declare no competing financial interest.

## Author contributions

Individuals listed as authors have contributed to developing this manuscript, and no other person was involved with its progress. The authors' contributions included: A. A.-G. carried out all the experiments and literature review, M.-A. M the X-ray diffraction studies and their respective analysis, and J. P. the composition of original draft, supervision, and sources. All authors have read and agreed to the published version of this paper.

## Supplementary Material

RA-013-D3RA02553E-s001

RA-013-D3RA02553E-s002
