# Supplementary material for: Synthesis of structural analogues of Reversan by ester aminolysis: an access to pyrazolo[1,5-a]pyrimidines from chalcones
Source: RSC Adv. 2023 May 31;13(24):16377–86. doi: 10.1039/d3ra02553e (PMC10230348; doi:10.1039/d3ra02553e)
Supplement: RA-013-D3RA02553E-s001 [file RA-013-D3RA02553E-s001.pdf]

Electronic Supplementary Material (ESI) for RSC Advances.
This journal is © The Royal Society of Chemistry 2023

Electronic supplementary information (ESI)

Synthesis of structural analogues of Reversan by ester aminolysis: An access to pyrazolo[1,5-*a*]pyrimidines from chalcones

Andres Arias-Gómez,[a] Mario A. Macías,[b] and Jaime Portilla*[a]

[a] Bioorganic Compounds Research Group & [b] Crystallography and Chemistry of Materials, Department of Chemistry, Universidad de Los Andes, Carrera 1 No. 18A-10, Bogotá 111711, Colombia

*E-mail: jportill@uniandes.edu.co; Tel: +57 1 3394949. Ext. 2080

Contents

1. **Overview of substrates and products numbering……S2**
2. **Synthesis and characterisation of precursors……S3**
3. **Copies of NMR spectra……S7**
4. **HRMS analysis……S36**
5. **Crystallographic details ……S52**
6. **References……S54**

1. Overview of substrates and products numbering

(a) Substrates/reagents: dibenzoylmethane (**3**), ethyl cyanoacetate (**7**), acetophenones **8a-f**, arylaldehydes **9a-f**, 3-aminocrotononitrile (**10**), hydrazine monohydrate (HM), *N,N*-dimethylformamide dimethyl acetal (DMF-DMA), ethyl 5-amino-1*H*-pyrazole-4-carboxylate (**1**), 3-methyl-1*H*-pyrazol-5-amine (**1'**), and chalcones **2a-h**.

Compounds purchased in our laboratory/price*

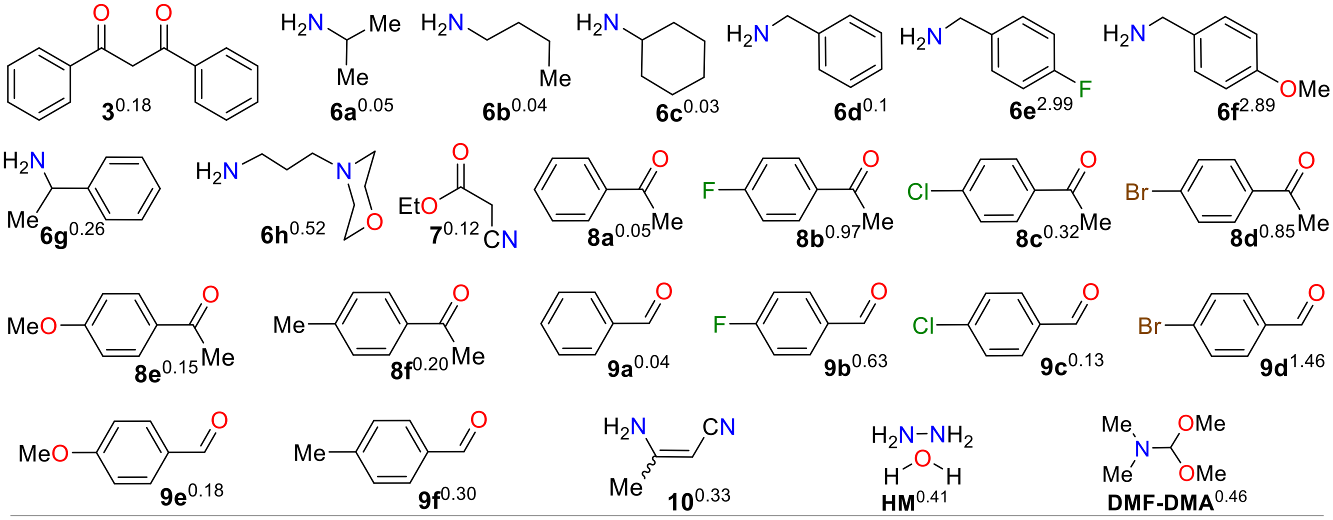

*Approximate prices in USD per gram or mililitre were consulted via MERCK (or Thermo Fisher Scientific) on March 29, 2023.

Compound synthesized in our laboratory*

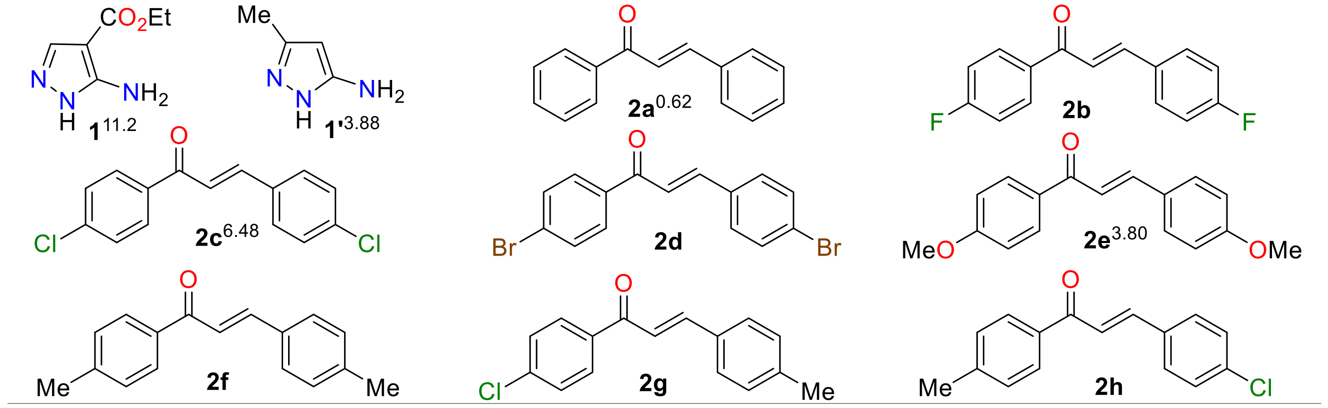

*These compounds were synthesised in our lab using simple protocols; however, some of the available prices are shown as in purchased reagents.

(b) 5,7-di-Aryl-2-methyl substituted pyrazolo[1,5-*a*]pyrimidines **4a'-c'**

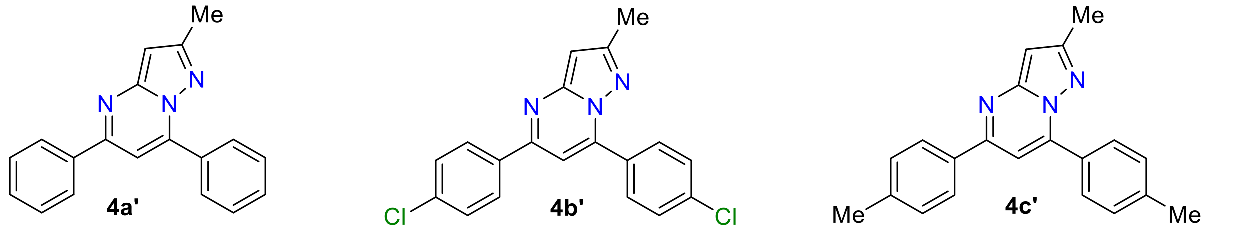

Fig. S1 Structures of (a) commercial (top) and synthetics (bottom) substrate/reagents. (b) Structures of PPs **4a'-c'**

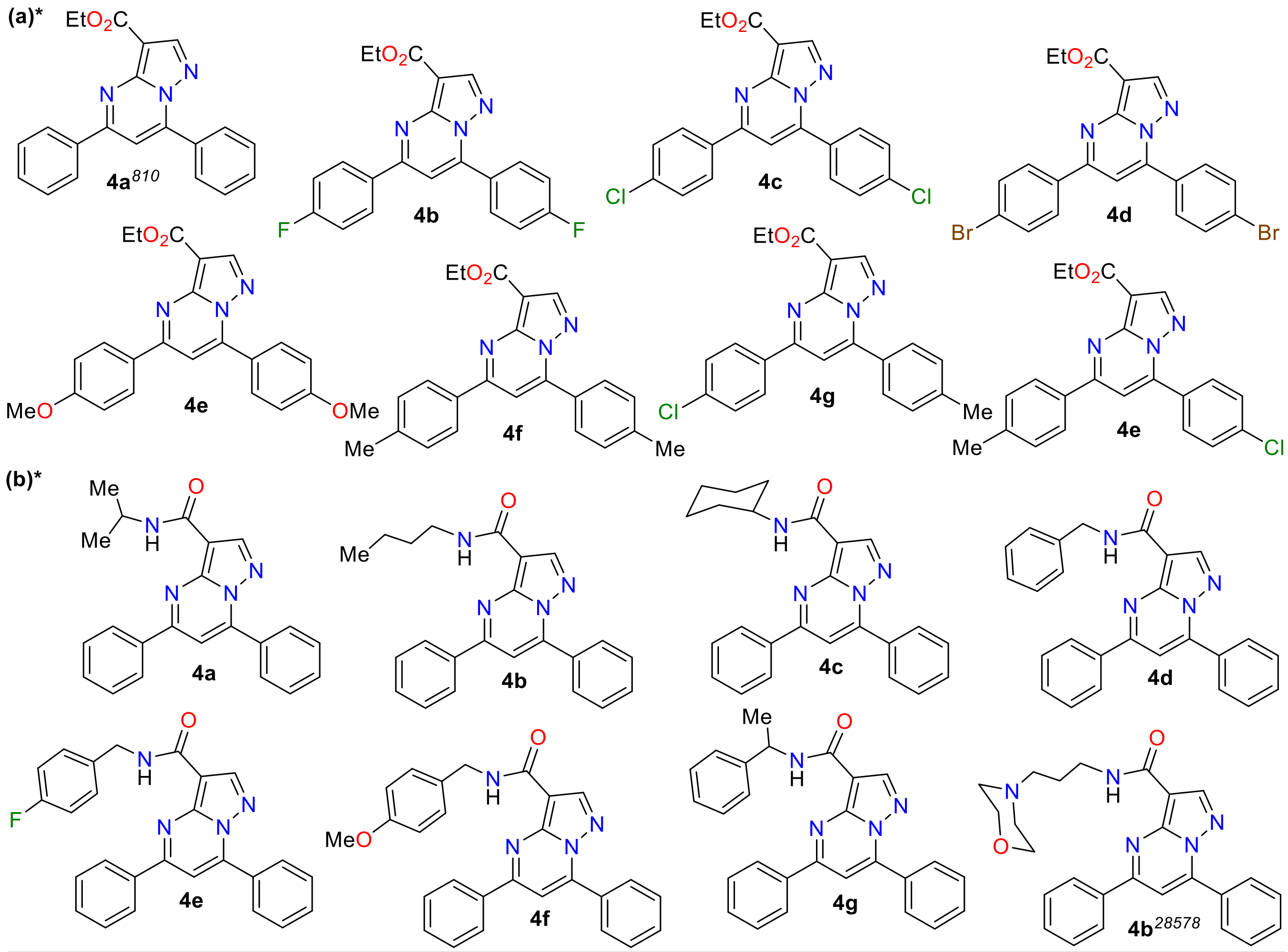

*Approximate prices in USD per gram of Reversan (MERCK) and its precursor (BIOSYNTH) were consulted on March 29, 2023.

Fig. S2 Structure of (a) esters **4a-h** and (b) amides **5a-h** containing the pyrazolo[1,5-*a*]pyrimidine ring.

2. Synthesis and characterisation of precursors

Ethyl 5-amino-1H-pyrazole-4-carboxylate (1). A mixture of ethyl cyanoacetate (**7**, 120 mg, 1.06 mmol),

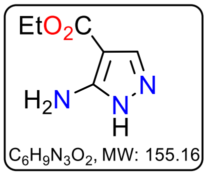

DMF (0.2 mL), acetic acid (0.3 mL), and DMF-DMA (220 mg dropwise was added, 1.85 mmol) was stirred for 1h at room temperature. To the resulting pale-yellow mixture, hydrazine monohydrate (110 mg, 2.20 mmol) dropwise was added at 0 °C, and the reaction mixture was stirred at 50 °C for 2h. After completion of the reaction, water was added (0.5 mL), and the mixture was extracted with ethyl acetate (3 × 2 mL). The combined organic layer was dried over anhydrous Na_2SO_4, filtered, and concentrated under reduced pressure giving a pale brown solid, which recrystallised from ethanol to afford the aminoester **1** as a white solid (135 mg, 85 %). For the multigram scale, 5.0 g of **7** (44.2 mmol), 5 mL of DMF, 7.5 mL of acetic acid, 8.7 g of DMF-DMA (73 mmol), and 4.5 g of HM (89.9 mmol) were used (6.04 g, 88%). Mp: 107-109 °C (Lit.[1] 102-103 °C). ^{1}H NMR (400 MHz,

$CDCl_3$): δ = 1.33 (t, *J* = 7.2 Hz, 3H), 4.27 (q, *J* = 7.2 Hz, 2H), 5.08 (s, 2H, NH_2, *br*), 7.71 (br, 1H, H5) ppm. ^{13}C NMR (100 MHz, $CDCl_3$) δ 14.5 (CH_3), 59.9 (CH_2), 97.8 (C), 136.2 (CH), 154.0 (C), 164.7 (C=O) ppm. These NMR data matched previously reported data.[1,2]

3-Methyl-1H-pyrazol-5-amine (1'). A mixture of 3-aminocrotononitrile (**10**, 86 mg, 1.05 mmol) and hydrazine monohydrate (HM, 76 mg, 1.52 mmol) was subjected to microwave irradiation at 120 °C (130 W, monitored by an IR temperature sensor) and maintained at this temperature for 30 min in a sealed MW tube of 10 mL. The resulting reaction mixture was cooled to room temperature by airflow, the excess of HM was removed under vacuum, and *n*-pentane was added (3 × 2 mL) to wash the mixture removing as much of the hydrazine as possible. The solid residue was purified by flash chromatography on silica gel (eluent: i. CH_2Cl_2 and ii. CH_2Cl_2/MeOH 20:1 v/v) to afford **1'** as a brown solid in high yield (81 mg, 79%). For the multigram scale, 3.0 g of **10** (36.4 mmol), 2.8 g of HM (58.9 mmol), and a sealed MW tube of 35 mL were used (2.97 g, 84%). Mp: 43-45 °C (Lit.[3] 48-49 °C). ^1H NMR (400 MHz, DMSO-d_6): δ= 2.04 (s, 3H), 4.45 (s-br, 8H, NH, NH_2, 2.5H_2O), 5.15 (s, 1H) ppm. ^{13}C NMR (100 MHz, DMSO-d_6) δ 11.5 (CH_3), 90.4 (CH), 140.3 (C), 154.0 (C) ppm. These NMR data matched previously reported data.[4]

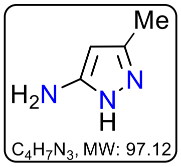

General procedure for the synthesis of chalcones 2a-h. A mixture of aryl methyl ketone **8a-f** (2.2 mmol), the appropriate arylaldehyde **9a-f** (2.0 mmol), and 50% aqueous NaOH (7 mL) in MeOH (7 mL) was stirred at room temperature for 15 min. After the reaction was completed (monitored by TLC), the precipitated product was filtered, washed with cool ethanol/water (~1:2, 3 mL), and recrystallised from ethanol to give the substituted chalcones 2a-g in high yields.

(E)-Chalcone (2a). By the general procedure with methyl phenyl ketone or acetophenone (**8a**, 266 mg, 2.2 mmol) and benzaldehyde (**9a**, 214 mg, 2.0 mmol), product **2a** was obtained as yellow crystals (376 mg, 90%). For the multigram scale, 2.74 g of **8a** (22.8 mmol), 2.20 g of **9a** (20.7 mmol), and 70 mL of aqueous NaOH/MeOH (1:1 v/v) were used (3.97 g, 92%). Mp: 57-58 °C (Lit.[5] 56-58 °C). ^1H NMR (400 MHz, $CDCl_3$): δ= 7.40-7.45 (m, 3H), 7.47-7.70 (m, 6H), 7.82 (d, *J* = 15.8 Hz, 1H), 8.03 (d, *J* = 8.0 Hz, 2H) ppm. ^{13}C NMR (100 MHz, $CDCl_3$): δ= 122.1 (CH), 128.5 (CH), 128.6 (CH), 128.7 (CH), 129.0 (CH), 130.6 (CH), 132.8 (CH), 134.9 (C), 138.2 (C), 144.9 (CH), 190.6 (C=O) ppm. These NMR data matched previously reported data in the literature.[5]

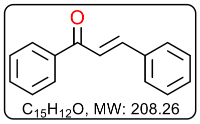

(E)-1,3-bis(4-fluorophenyl)prop-2-en-1-one (2b). By the general procedure with 4'-fluoroacetophenone (**8b**, 305 mg, 2.2 mmol) and 4-fluorobenzaldehyde (**9b**, 250 mg, 2.0 mmol), product **2b** was obtained as white crystals (2.22 g, 91%). Mp: 116-118 °C (Lit.[5] 98-99 °C). ^1H NMR (400 MHz, $CDCl_3$): δ= 7.11 (t, *J* = 8.6 Hz, 2H), 7.18 (t, *J* = 8.5 Hz, 2H), 7.43 (d, *J* = 15.7 Hz, 1H), 7.64 (dd, *J* = 8.5 Hz, 2H), 7.78 (d, *J* = 15.7 Hz, 1H), 8.06 (dd, *J* = 8.7 Hz, 2H) ppm. ^{13}C NMR (100 MHz, $CDCl_3$): δ= 115.7/116.1 (CH, d, *J* = 22.1 Hz), 115.9/116.3 (CH, d, *J* = 22.0 Hz), 121.2 (CH), 130.4/130.5 (CH, d, *J* = 8.8 Hz), 131.0 (C, d, *J* = 2.2 Hz), 131.0/131.1 (CH, d, *J* = 8.9 Hz), 130.4 (Co, d, *J* = 8.8 Hz), 134.4 (C, d, *J* =

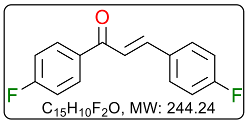

2.9 Hz), 143.7 (CH), 162.9/165.4 (CF, d, *J* = 251.6 Hz), 164.4/166.9 (CF, d, *J* = 254.6 Hz), 188.6 (C=O) ppm. These NMR data matched previously reported data in the literature.[5]

(E)-1,3-bis(4-Chlorophenyl)prop-2-en-1-one (2c). By the general procedure with 4'-chloroacetophenone

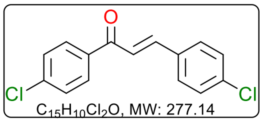

(**8c**, 340 mg, 2.2 mmol) and 4-chlorobenzaldehyde (**9c**, 282 mg, 2.0 mmol), product **2c** was obtained as yellow crystals (2.64 g, 95%). Mp: 155-156 °C (Lit.[6] 154-155 °C). ^{1}H NMR (400 MHz, $CDCl_3$): δ= 7.36-7.51 (m, 5H), 7.57 (d, *J* = 8.6 Hz, 2H), 7.76 (d, *J* = 15.6 Hz, 1H), 7.96 (d, *J* = 8.7 Hz, 2H) ppm. ^{13}C NMR (100 MHz, $CDCl_3$): δ= 121.9 (CH), 129.0 (CH), 129.3 (CH), 129.7 (CH), 129.9 (CH), 133.2 (C), 136.4 (C), 136.7 (C), 139.4 (C), 143.8 (CH), 188.9 (C=O). These NMR data matched previously reported data in the literature.[6]

(E)-1,3-bis(4-Bromophenyl)prop-2-en-1-one (2d). By the general procedure with 4'-bromoacetophenone

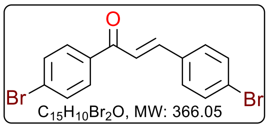

(**8d**, 458 mg, 2.2 mmol) and 4-bromobenzaldehyde (**9d**, 370 mg, 2.0 mmol), product **2d** was obtained as yellow crystals (3.47 g, 95%). Mp: 186-187 °C (Lit.[6] 183-185 °C). ^{1}H NMR (400 MHz, $CDCl_3$): δ= 7.44-7.58 (m, 5H), 7.65 (d, *J* = 7.6 Hz, 2H), 7.75 (d, *J* = 15.8 Hz, 1H), 7.88 (d, *J* = 7.7 Hz, 2H) ppm. ^{13}C NMR (100 MHz, $CDCl_3$): δ= 121.9 (CH), 125.1 (CH), 128.2 (CH), 129.9 (CH), 130.0 (CH), 132.0 (C), 132.3 (C), 133.6 (C), 136.7 (C), 144.0 (CH), 189.1 (C=O) ppm. These NMR data matched previously reported data in the literature.[6]

(E)-1,3-bis(4-Methoxyphenyl)prop-2-en-1-one (2e). By the reaction of 4'-methoxyacetophenone (**8e**, 330

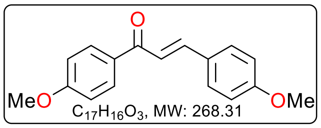

mg, 2.2 mmol) with 4-chlorobenzaldehyde (**9e**, 272 mg, 2.0 mmol), product **2e** was obtained as yellow crystals (2.55 g, 95%). Mp: 101-102 °C (Lit.[6] 101-103 °C). ^{1}H NMR (400 MHz, $CDCl_3$): δ= 3.84 (s, 3H), 3.88 (s, 3H), 6.92-6.98 (m, 4H), 7.43 (d, *J* = 15.5 Hz, 1H), 7.60 (d, *J* = 8.8 Hz, 2H), 7.78 (d, *J* = 15.5 Hz, 1H), 8.03 (d, *J* = 8.9 Hz, 2H) ppm. ^{13}C NMR (100 MHz, $CDCl_3$): δ= 55.4 (CH_3), 55.4 (CH_3), 113.8 (CH), 114.4 (CH), 119.6 (CH), 127.8 (C), 130.1 (CH), 130.7 (CH), 131.4 (C), 143.8 (CH), 161.5 (C), 163.3 (C), 188.8 (C=O). These NMR data matched previously reported data in the literature.[6]

(E)-1,3-di-p-Tolylprop-2-en-1-one (2f). By the general procedure with 4'-methylacetophenone (**8f**, 295 mg,

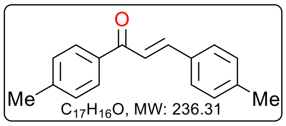

2.2 mmol) and 4-methylbenzaldehyde (**9f**, 240 mg, 2.0 mmol), product **2f** was obtained as yellow/green crystals (2.00 g, 85%). Mp: 129-130 °C (Lit.[6] 128-129 °C). ^{1}H NMR (400 MHz, $CDCl_3$): δ= 2.16 (s, 3H), 2.20 (s, 3H), 7.22 (d, *J* = 8.1 Hz, 2H), 7.29 (d, *J* = 8.1 Hz, 2H), 7.47-7.57 (m, 3H), 7.80 (d, *J* = 15.7 Hz, 1H), 7.94 (d, *J* = 8.2 Hz, 2H) ppm. ^{13}C NMR (100 MHz, $CDCl_3$): δ= 21.5 (CH_3), 21.7 (CH_3), 121.0 (CH), 128.4 (CH), 128.6 (CH), 129.3 (CH), 129.7 (CH), 132.2 (C), 135.7 (C), 140.9 (C), 143.5 (C), 144.5 (CH), 190.1 (C=O) ppm. These NMR data matched previously reported data in the literature.[6]

(E)-1-(4-Chlorophenyl)-3-(p-tolyl)prop-2-en-1-one (2g). By the reaction of 4'-chloroacetophenone (**8c**, 340 mg, 2.2 mmol) and 4-methylbenzaldehyde (**9f**, 240 mg, 2.0 mmol), product **2g** was obtained as yellow crystals (2.54 g, 99%). Mp: 149-150 °C (Lit.[7] 147-149 °C). ^{1}H NMR (400 MHz, $CDCl_3$): δ= 2.44 (s, 3H), 7.31 (d, *J* = 8.2 Hz, 2H), 7.39 (d, *J* = 8.4 Hz, 2H), 7.51 (d, *J* = 15.7 Hz, 1H), 7.58 (d, *J* = 8.6 Hz, 2H), 7.75 (d, *J* = 15.7 Hz, 1H), 7.93 (d, *J* = 8.2 Hz, 2H) ppm. ^{13}C NMR (100 MHz, $CDCl_3$): δ= 21.7 (CH_3), 122.5 (CH), 128.7 (CH), 129.3 (CH), 129.4 (CH), 129.6 (CH), 133.5 (C), 135.5 (C), 136.3 (C), 142.9 (C), 143.9 (C), 189.7 (C=O) ppm. These NMR data matched previously reported data in the literature.[7]

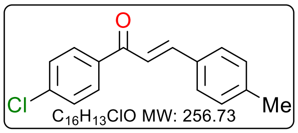

(E)-3-(4-chlorophenyl)-1-(p-tolyl)prop-2-en-1-one (2h). By the reaction of 4'-methylacetophenone (**8f**, 295 mg, 2.2 mmol) and 4-chlorobenzaldehyde (**9c**, 282 mg, 2.0 mmol), product **2h** was obtained as yellow crystals (2.52 g, 98%). Mp: 161-162 °C (Lit.[7] 158-160 °C). ^{1}H NMR (400 MHz, $CDCl_3$): δ= 2.40 (s, 3H), 7.24 (d, *J* = 8.1 Hz, 2H), 7.42-7.50 (m, 3H), 7.55 (d, *J* = 8.1 Hz, 2H), 7.80 (d, *J* = 15.7 Hz, 1H), 7.97 (d, *J* = 8.7 Hz, 2H) ppm. ^{13}C NMR (100 MHz, $CDCl_3$): δ= 21.6 (CH_3), 120.5 (CH), 128.6 (CH), 128.6 (CH), 129.8 (CH), 129.9 (CH), 132.0 (C), 136.7 (C), 139.1 (C), 141.4 (C), 145.5 (CH), 189.3 (C=O) ppm. These NMR data matched previously reported data in the literature.[7]

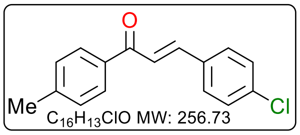

3. Copies of NMR spectra

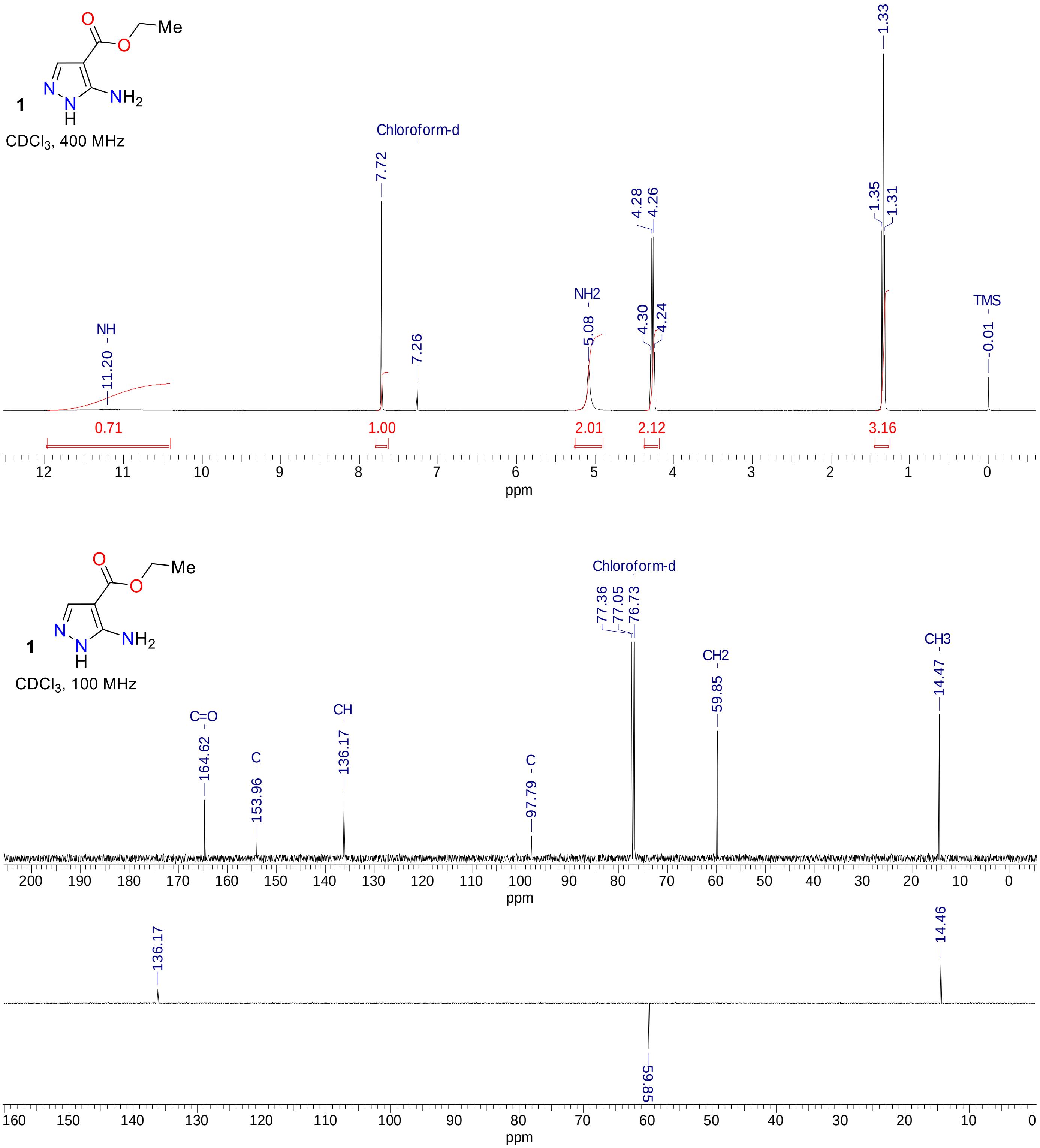

Fig. S3 ^{1}H, $^{13}C\{^{1}H\}$, and DEPT-135 NMR spectra of ethyl 5-amino-1*H*-pyrazole-4-carboxylate (**1**)

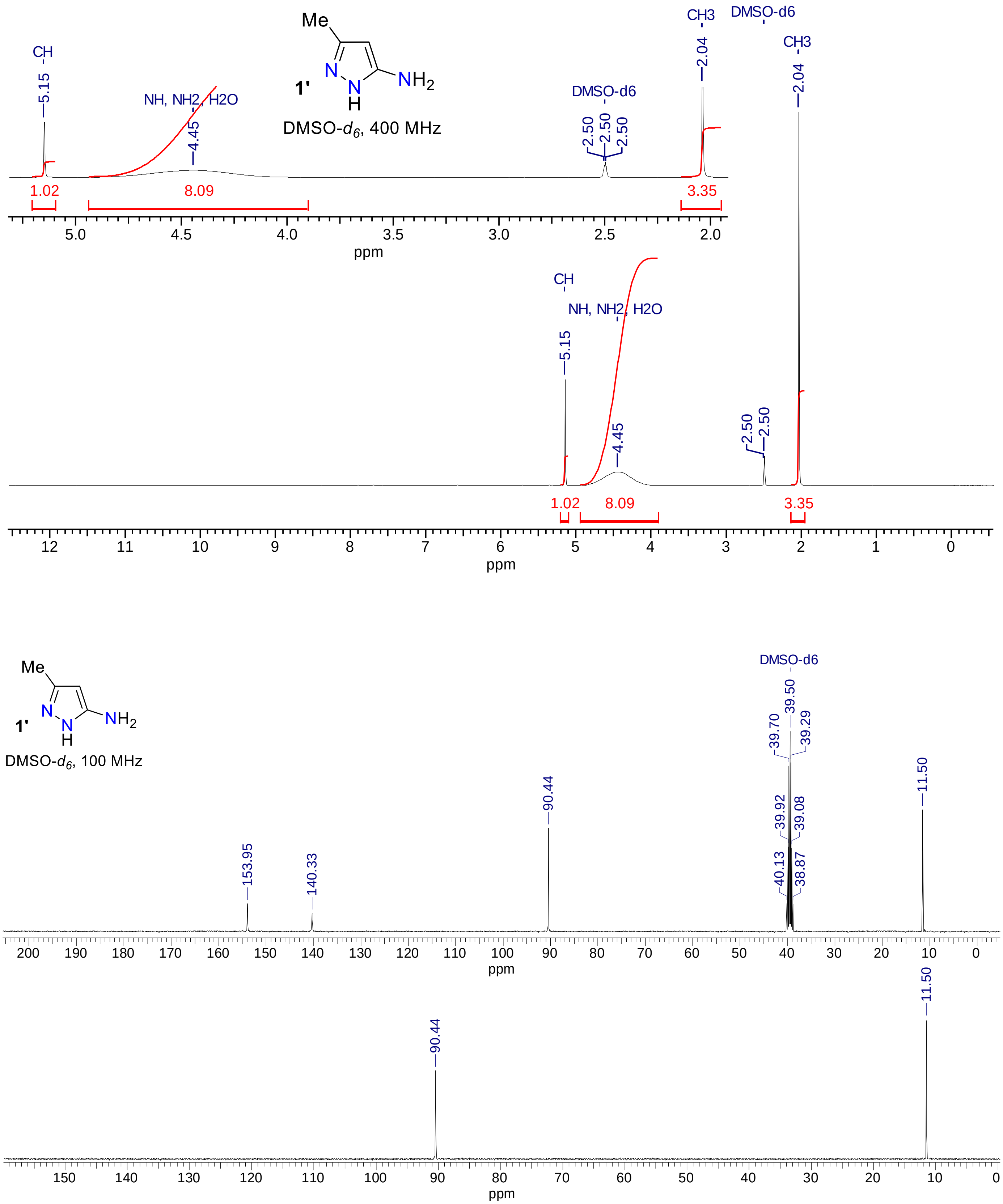

Fig. S4 ^{1}H, $^{13}C\{^{1}H\}$, and DEPT-135 NMR spectra of NMR spectrum for 3-methyl-1*H*-pyrazol-5-amine (**1'**)

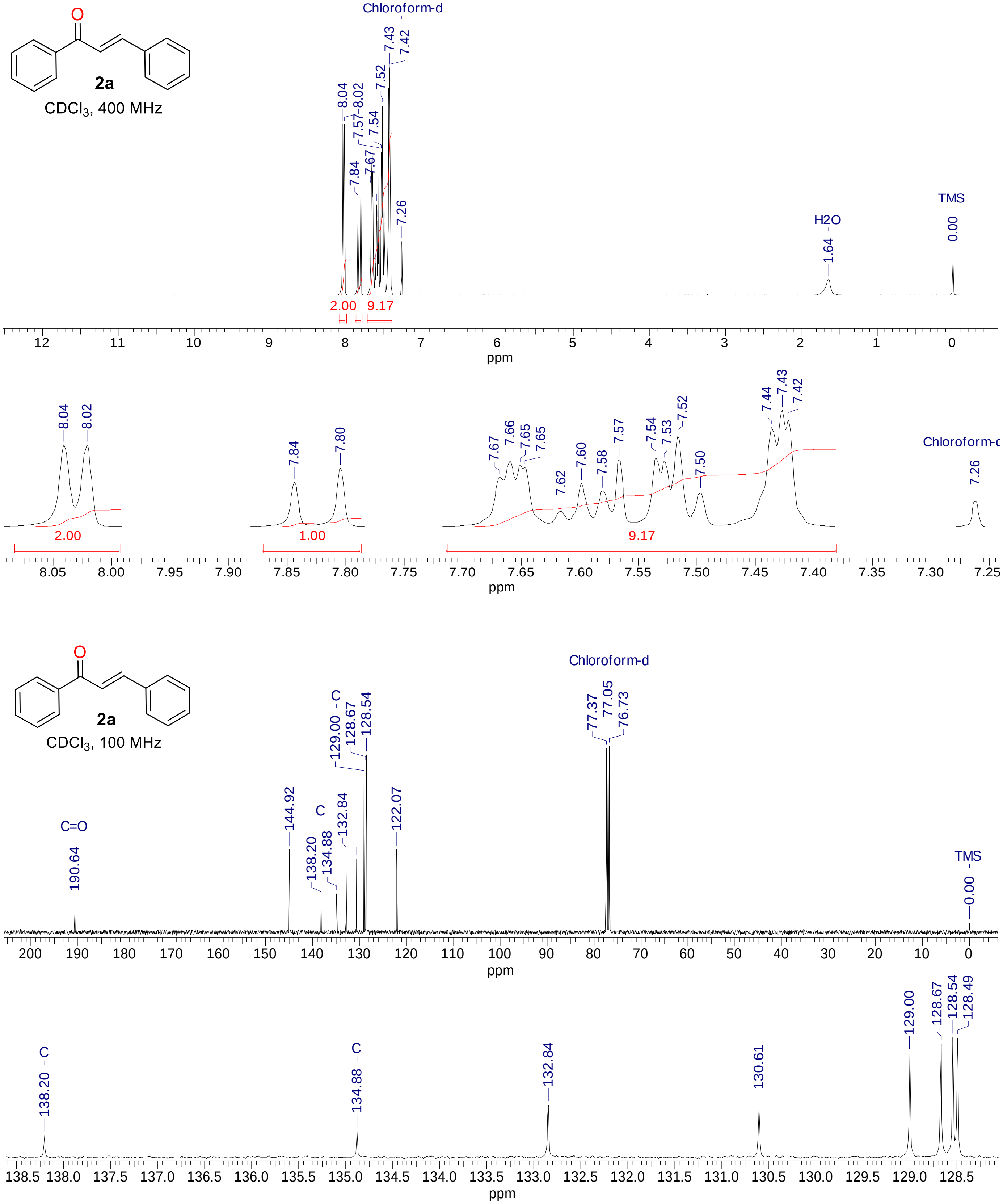

Fig. S5 ^{1}H and ^{13}C{^{1}H} of (*E*) chalcone (**2a**)

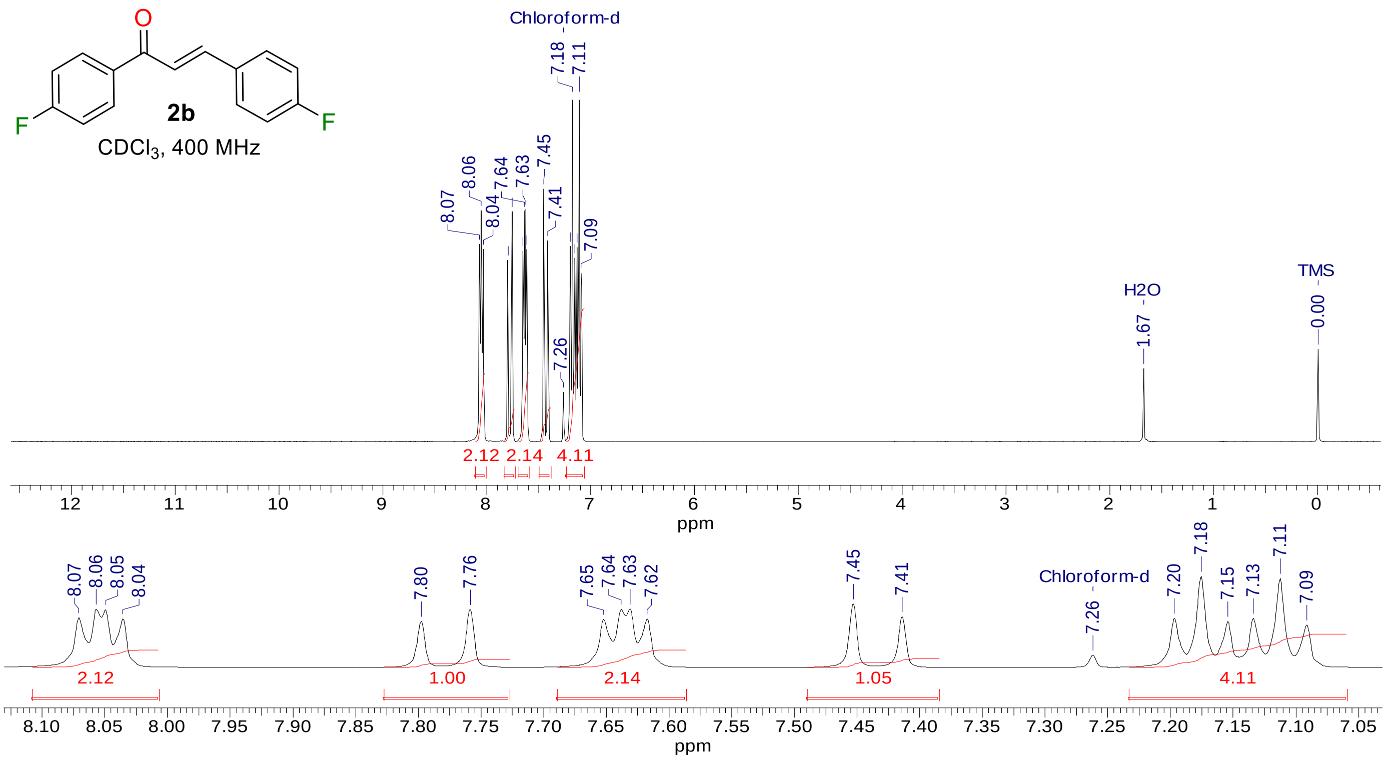

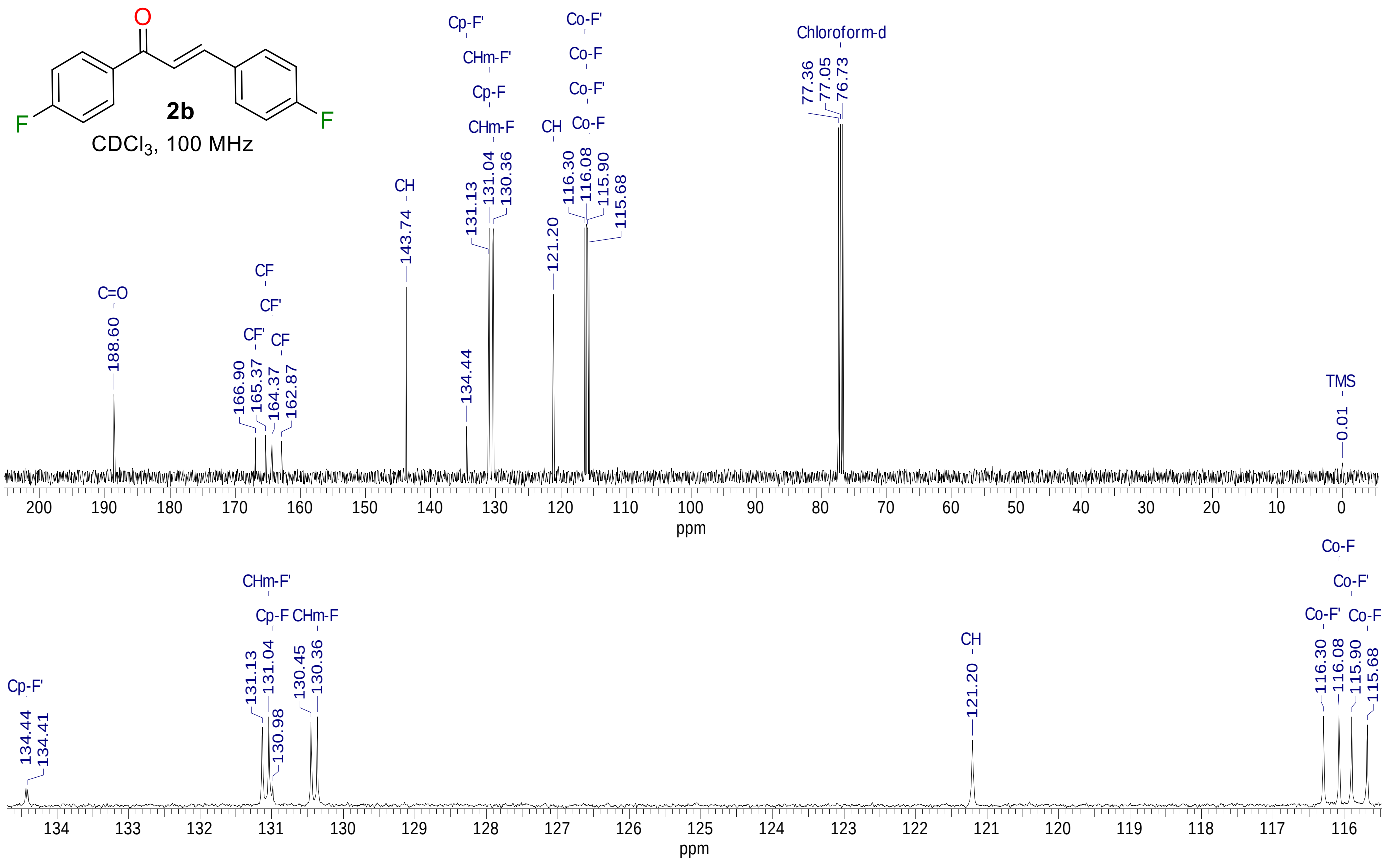

Fig. S6 ^{1}H and $^{13}C\{^{1}H\}$ of (*E*)-1,3-bis(4-fluorophenyl)prop-2-en-1-one (**2b**)

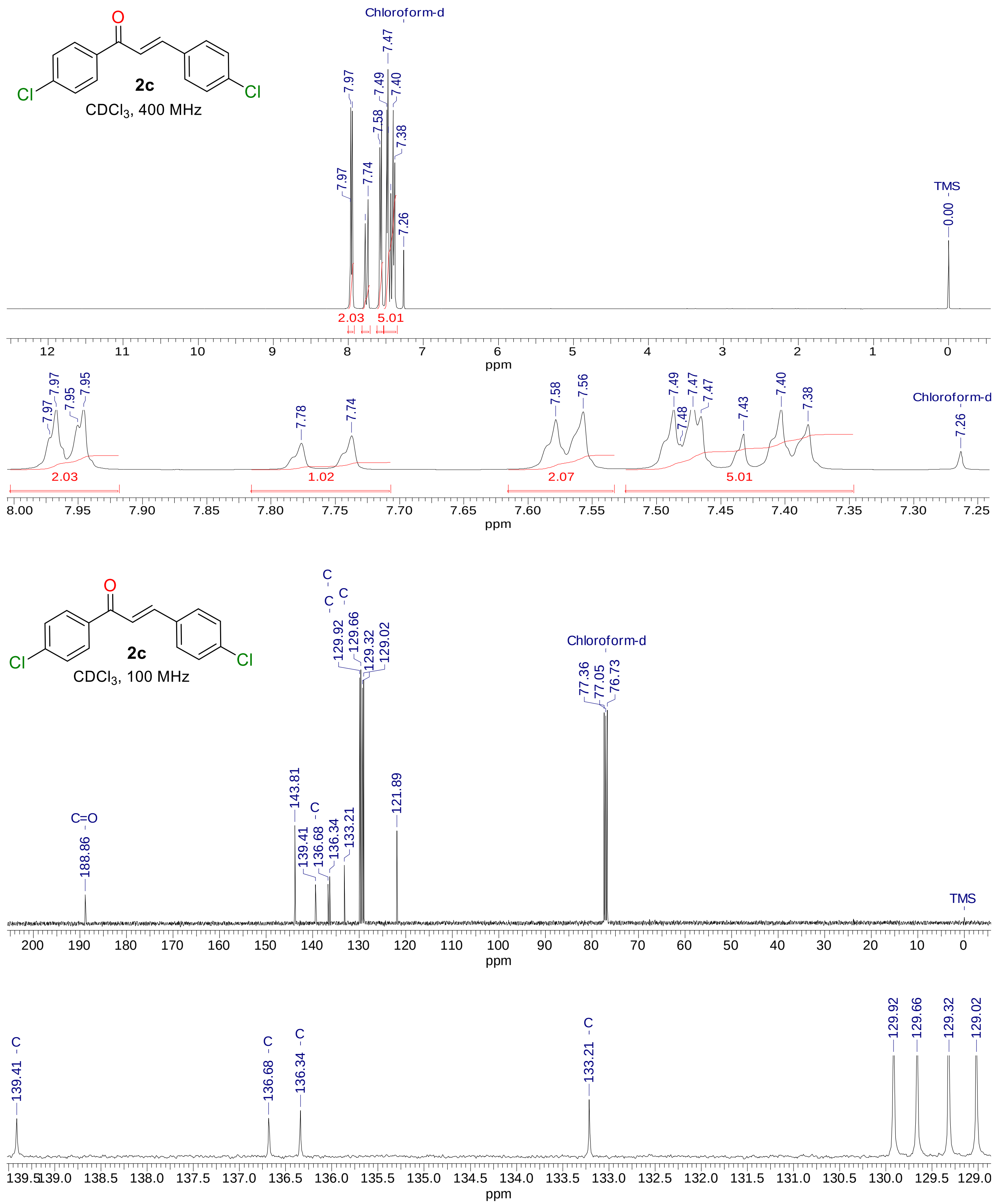

Fig. S7 ^{1}H and $^{13}C\{^{1}H\}$ of (*E*)-1,3-bis(4-chlorophenyl)prop-2-en-1-one (**2c**)

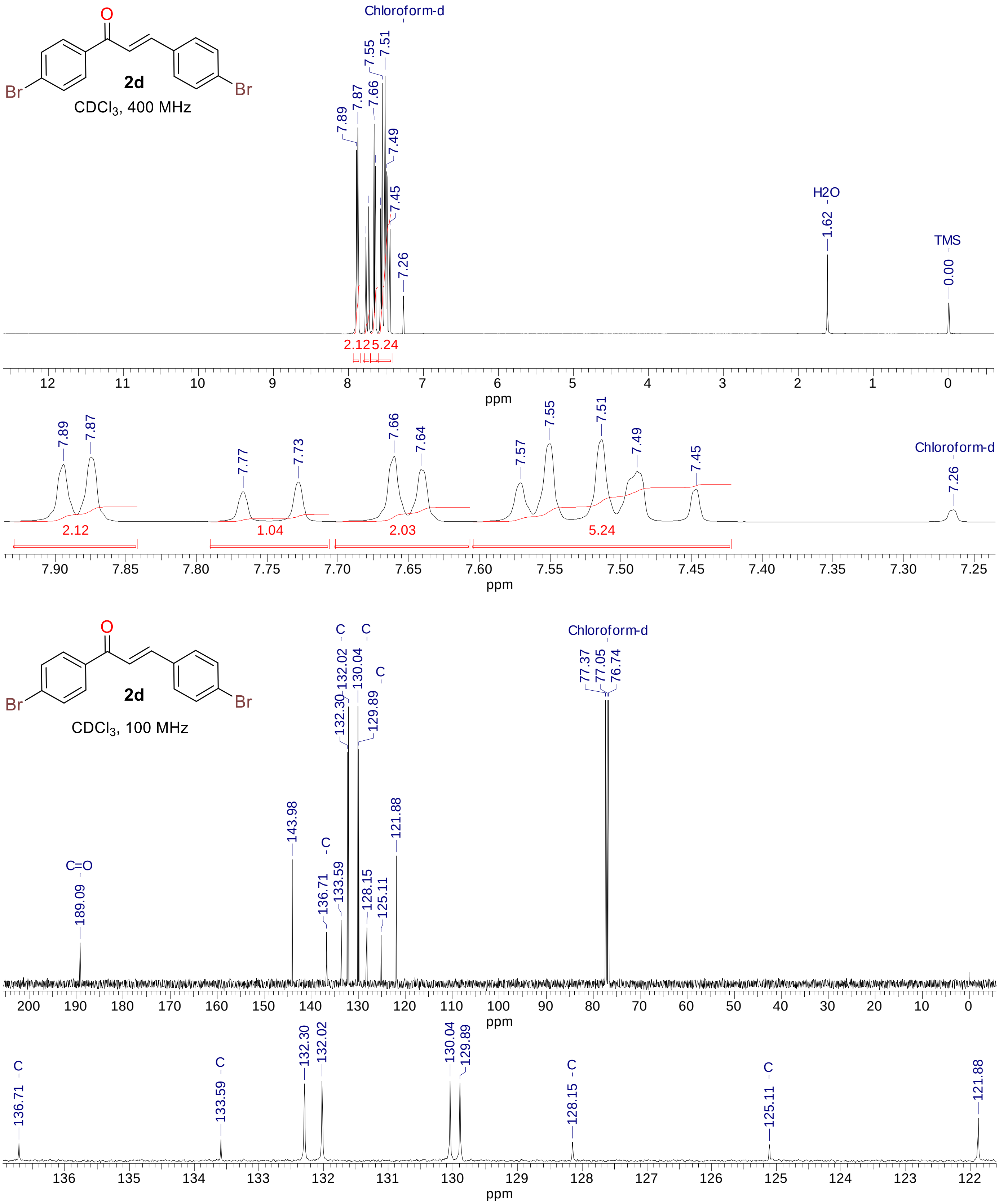

Fig. S8 ^{1}H and ^{13}C{^{1}H} of (*E*)-1,3-bis(4-bromophenyl)prop-2-en-1-one (**2d**)

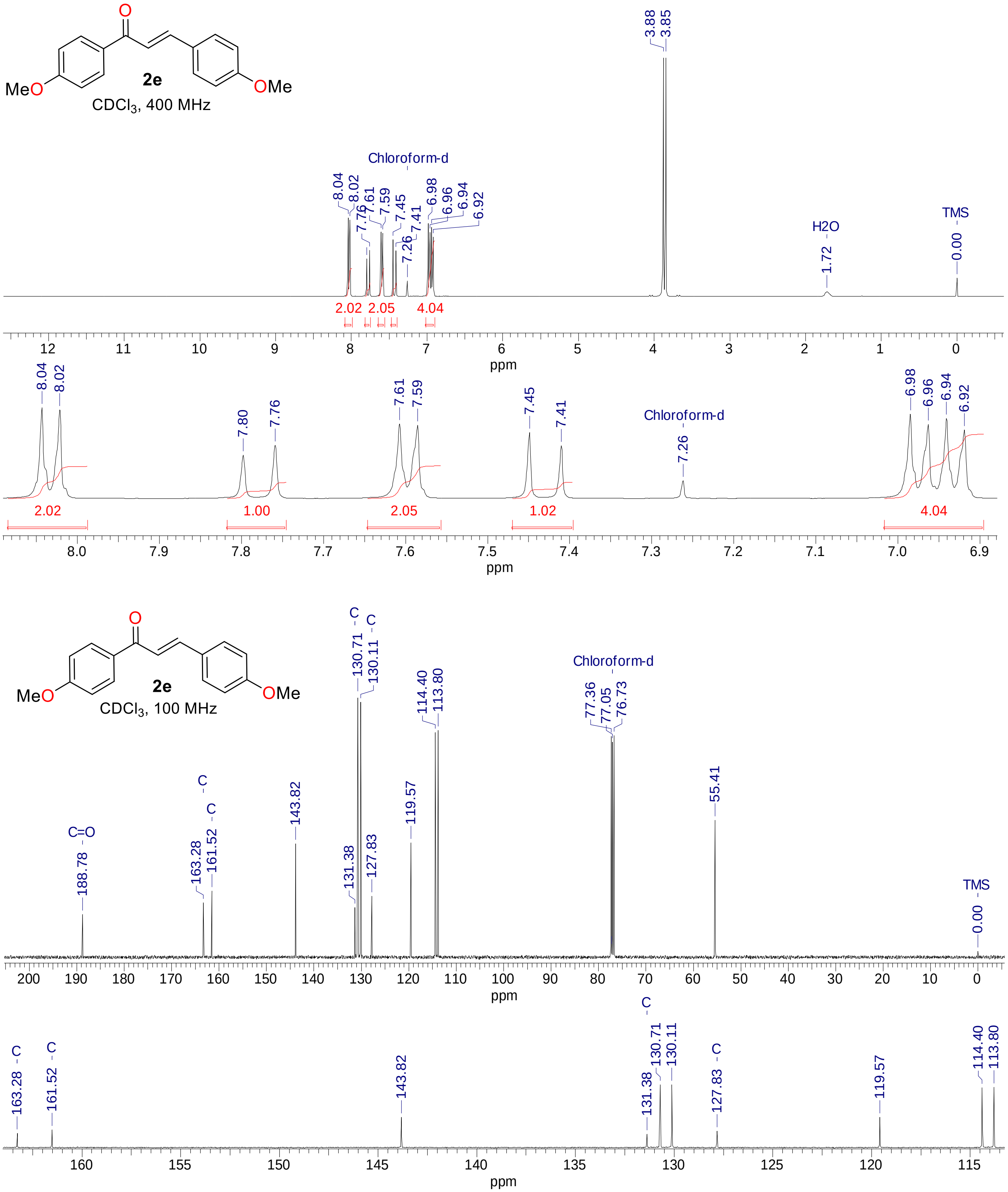

Fig. S9 ^{1}H and $^{13}C\{^{1}H\}$ of (*E*)-1,3-bis(4-methoxyphenyl)prop-2-en-1-one (**2e**)

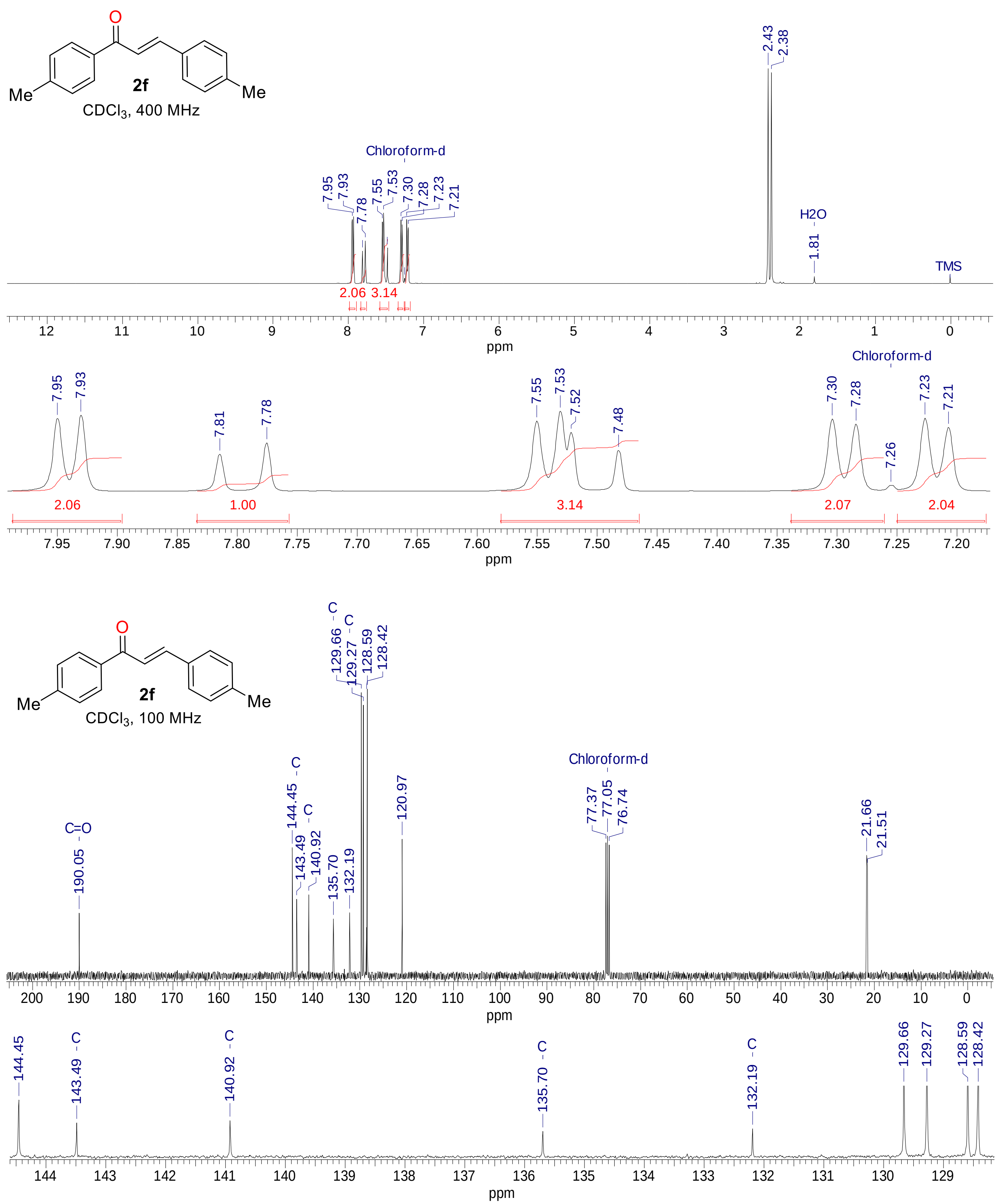

Fig. S10 ^{1}H and $^{13}C\{^{1}H\}$ of (*E*)-1,3-di-*p*-tolylprop-2-en-1-one (**2f**)

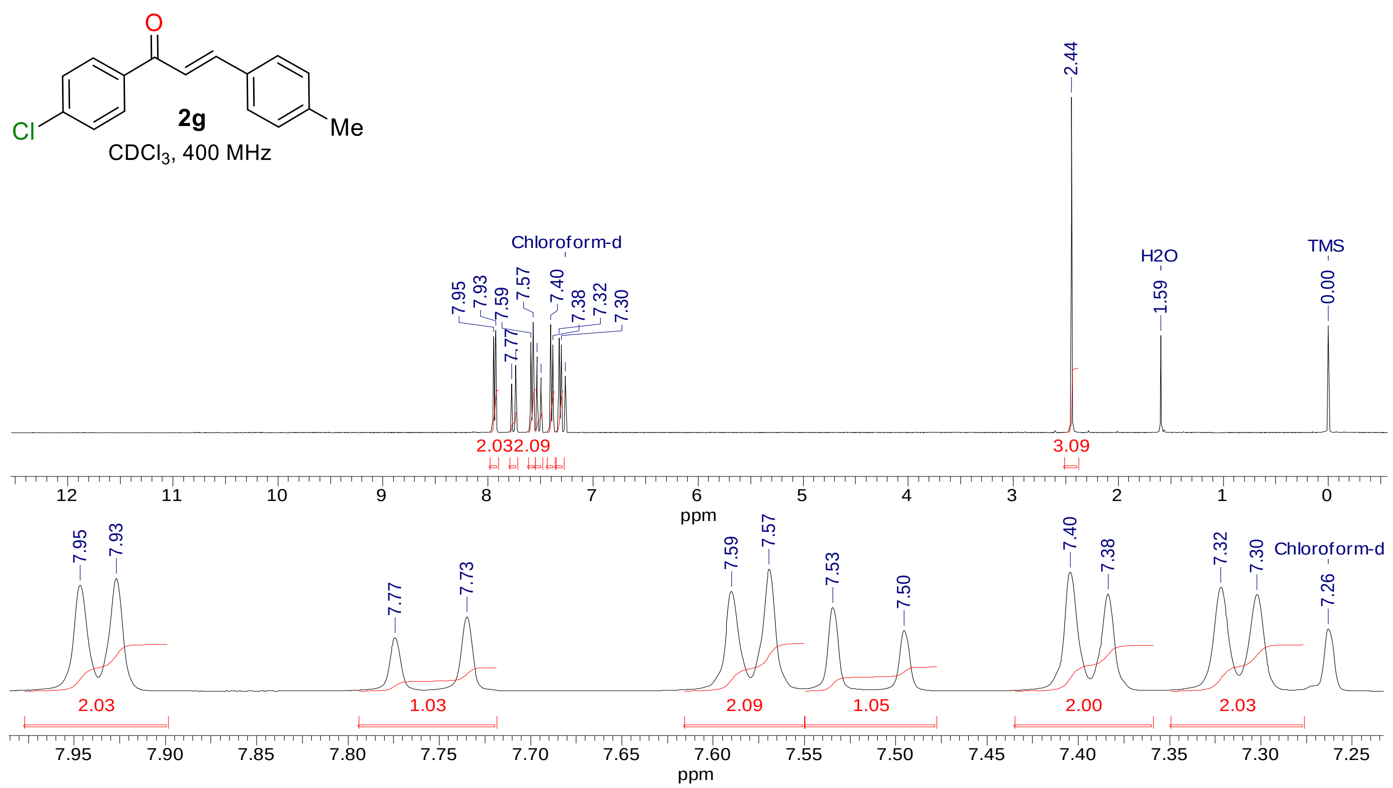

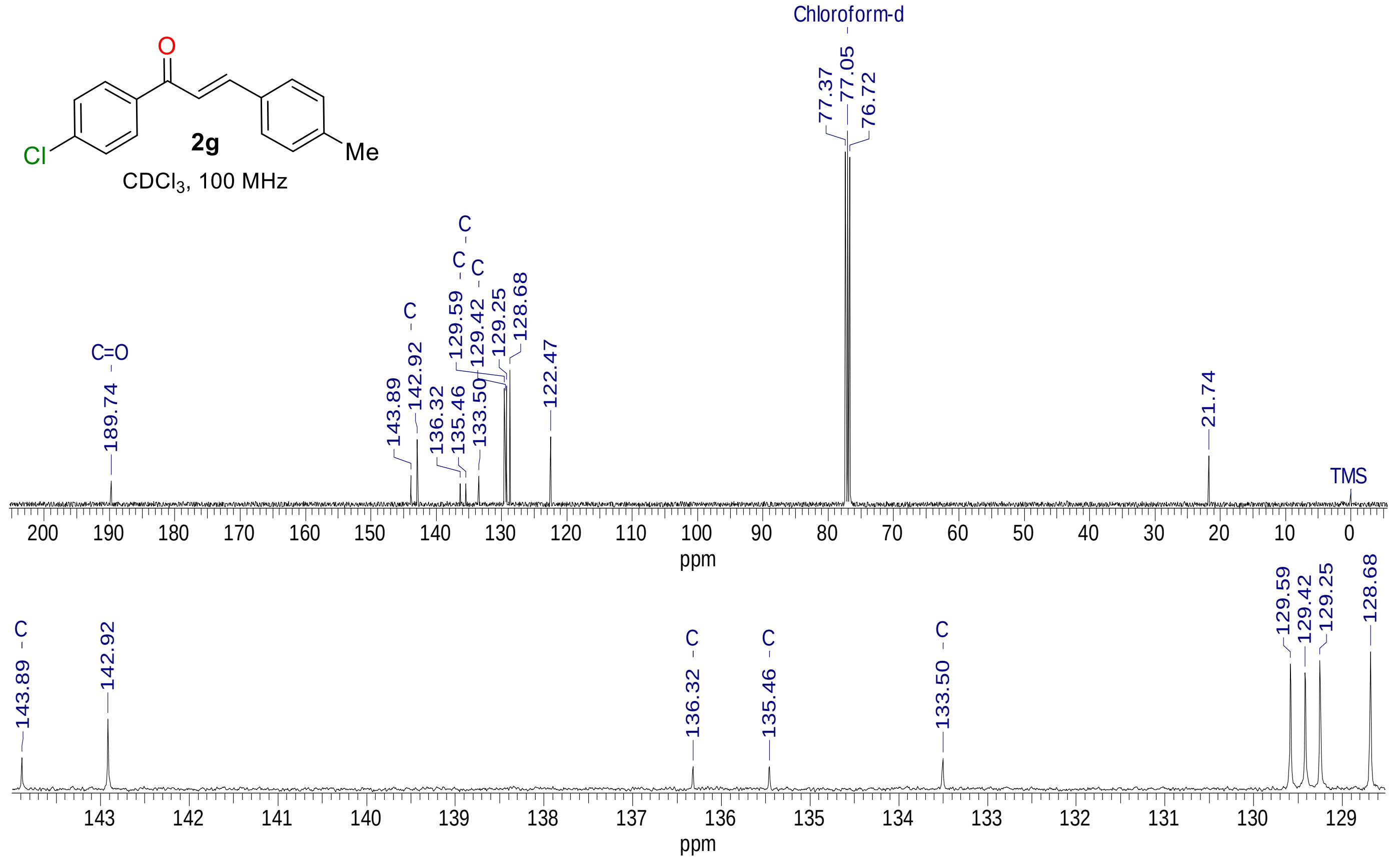

Fig. S11 ^{1}H and ^{13}C{^{1}H} of (*E*)-1-(4-chlorophenyl)-3-(*p*-tolyl)prop-2-en-1-one (**2g**)

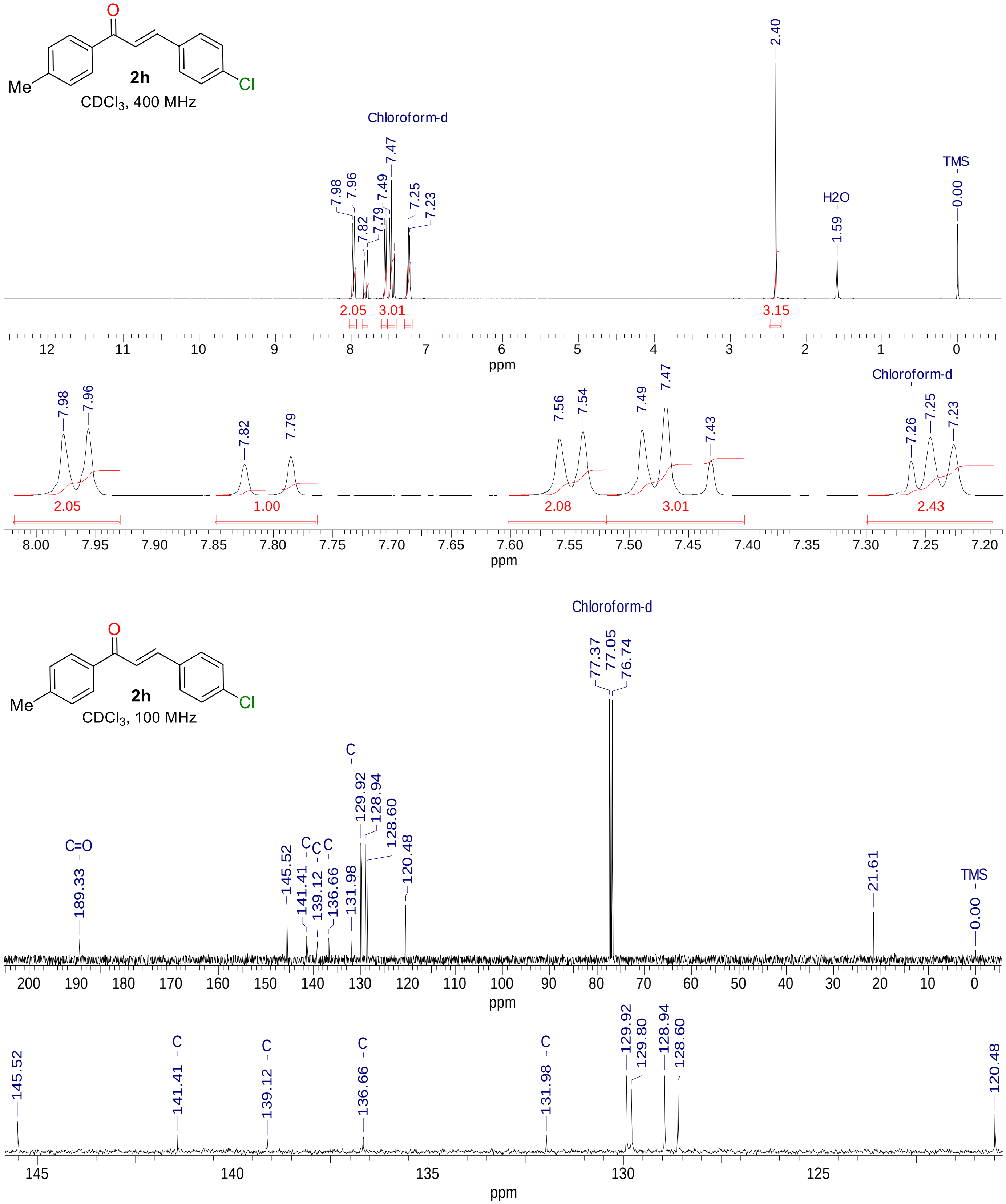

Fig. S12 ^{1}H and ^{13}C{^{1}H} of (*E*)-3-(4-chlorophenyl)-1-(*p*-tolyl)prop-2-en-1-one (**2h**)

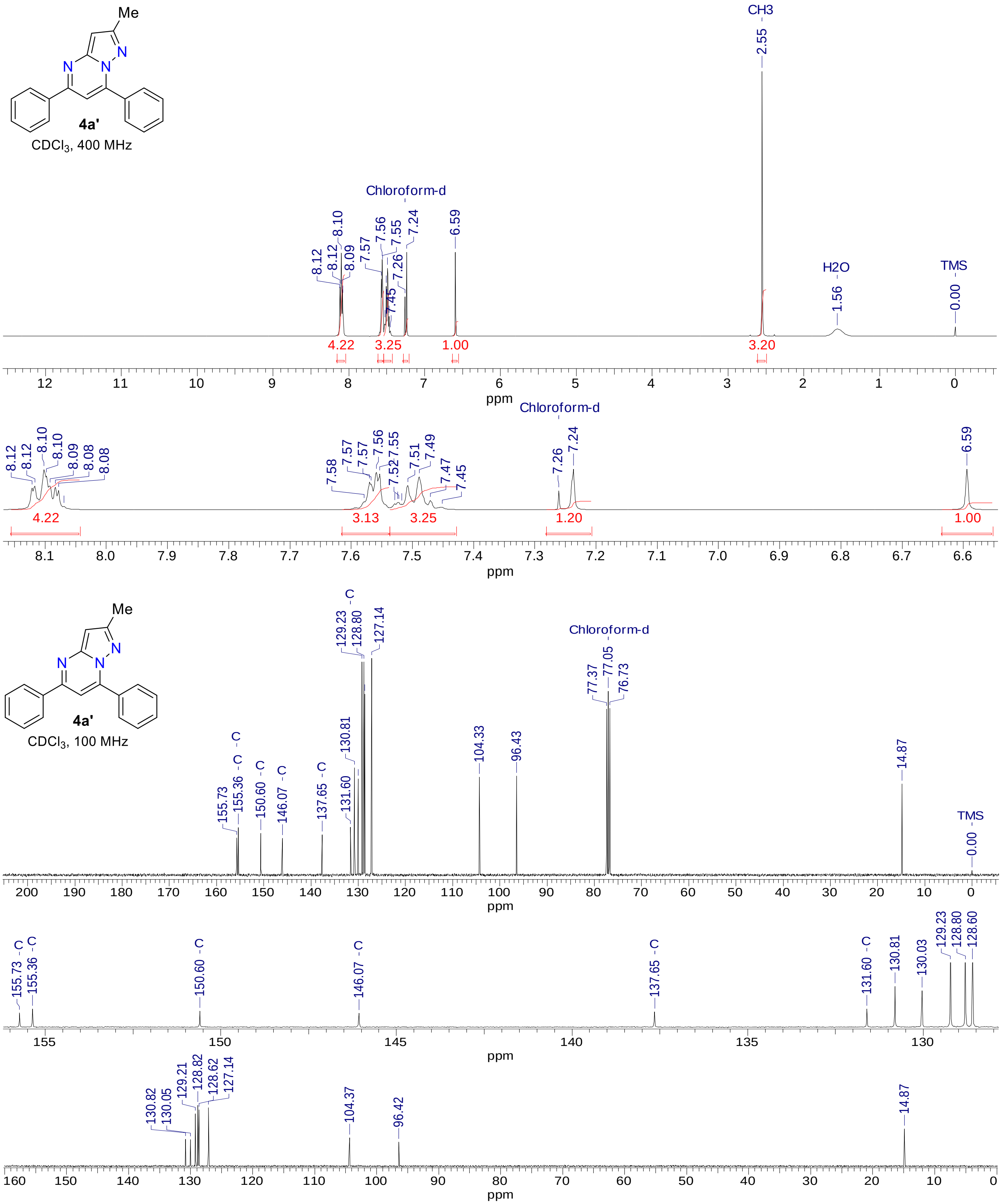

Fig. S13 ^{1}H, $^{13}C\{^{1}H\}$, and DEPT-135 NMR spectra of 2-methyl-5,7-diphenylpyrazolo[1,5-*a*]pyrimidine (**4a'**)

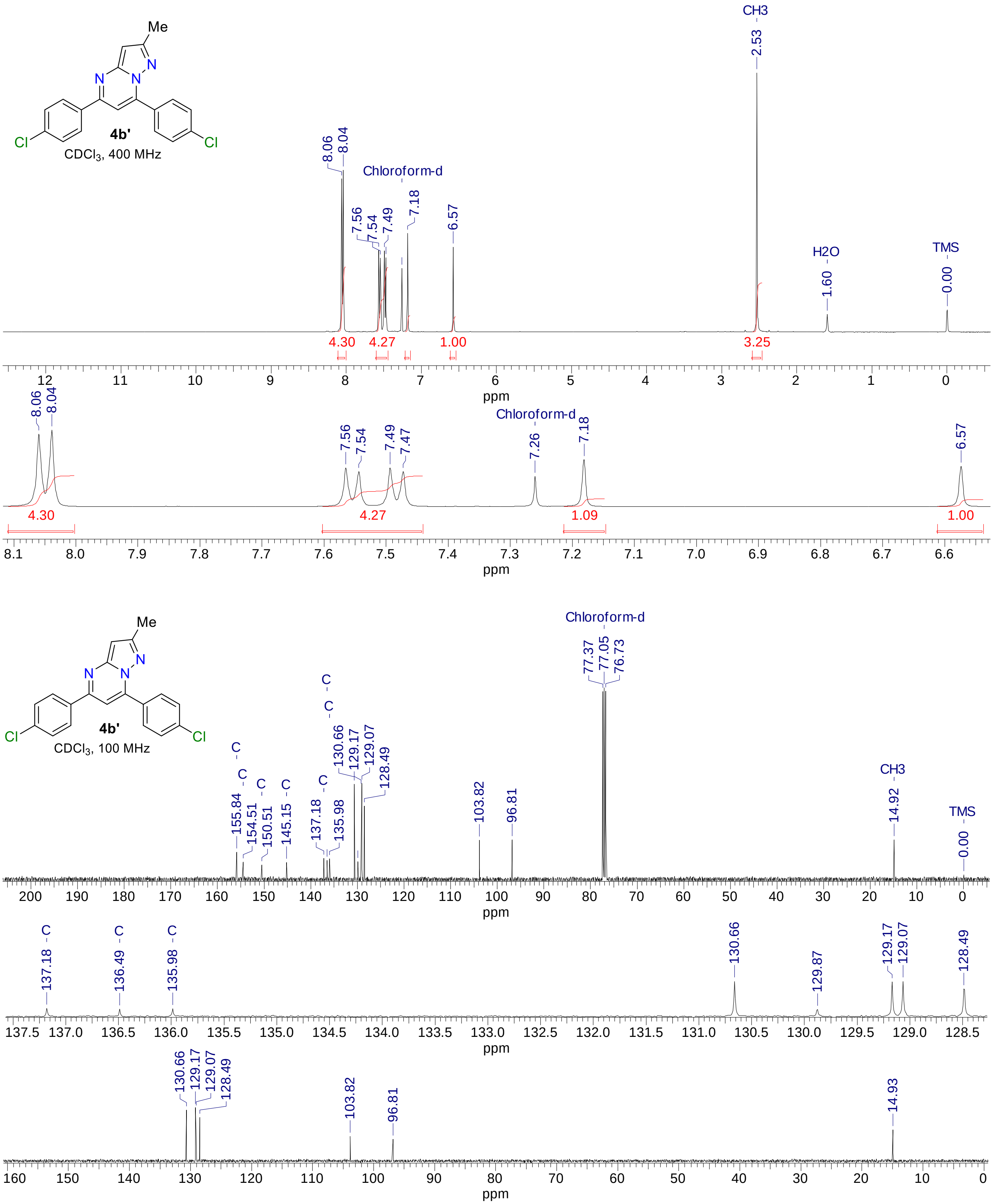

Fig. S14 ^{1}H, $^{13}C\{^{1}H\}$, and DEPT-135 NMR spectra of 5,7-bis(4-chlorophenyl)-2-methylpyrazolo[1,5-*a*]pyrimidine (**4b'**)

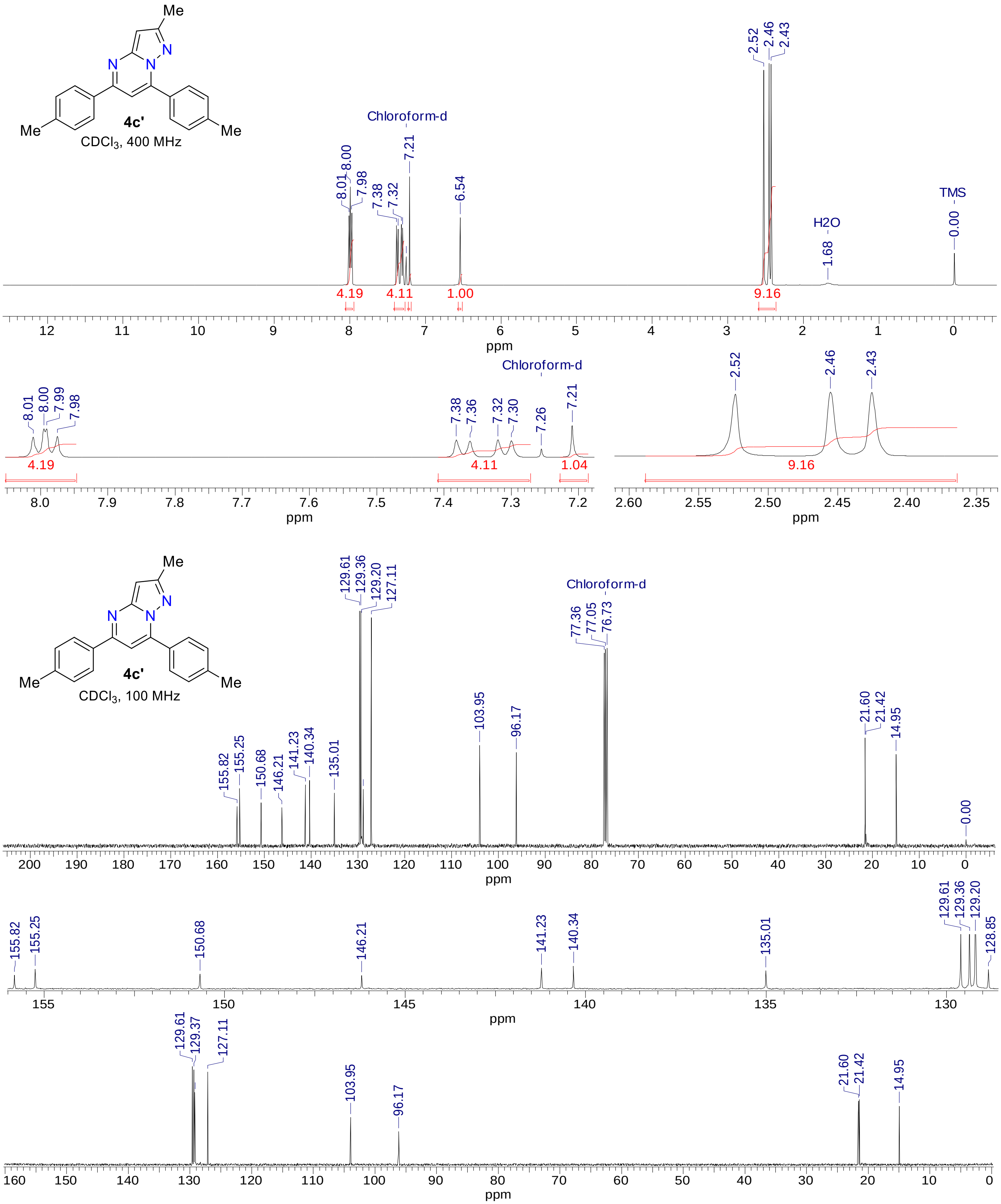

Fig. S15 ^{1}H, $^{13}C\{^{1}H\}$, and DEPT-135 NMR spectra of 2-methyl-5,7-di-p-tolylpyrazolo[1,5-*a*]pyrimidine (**4c'**)

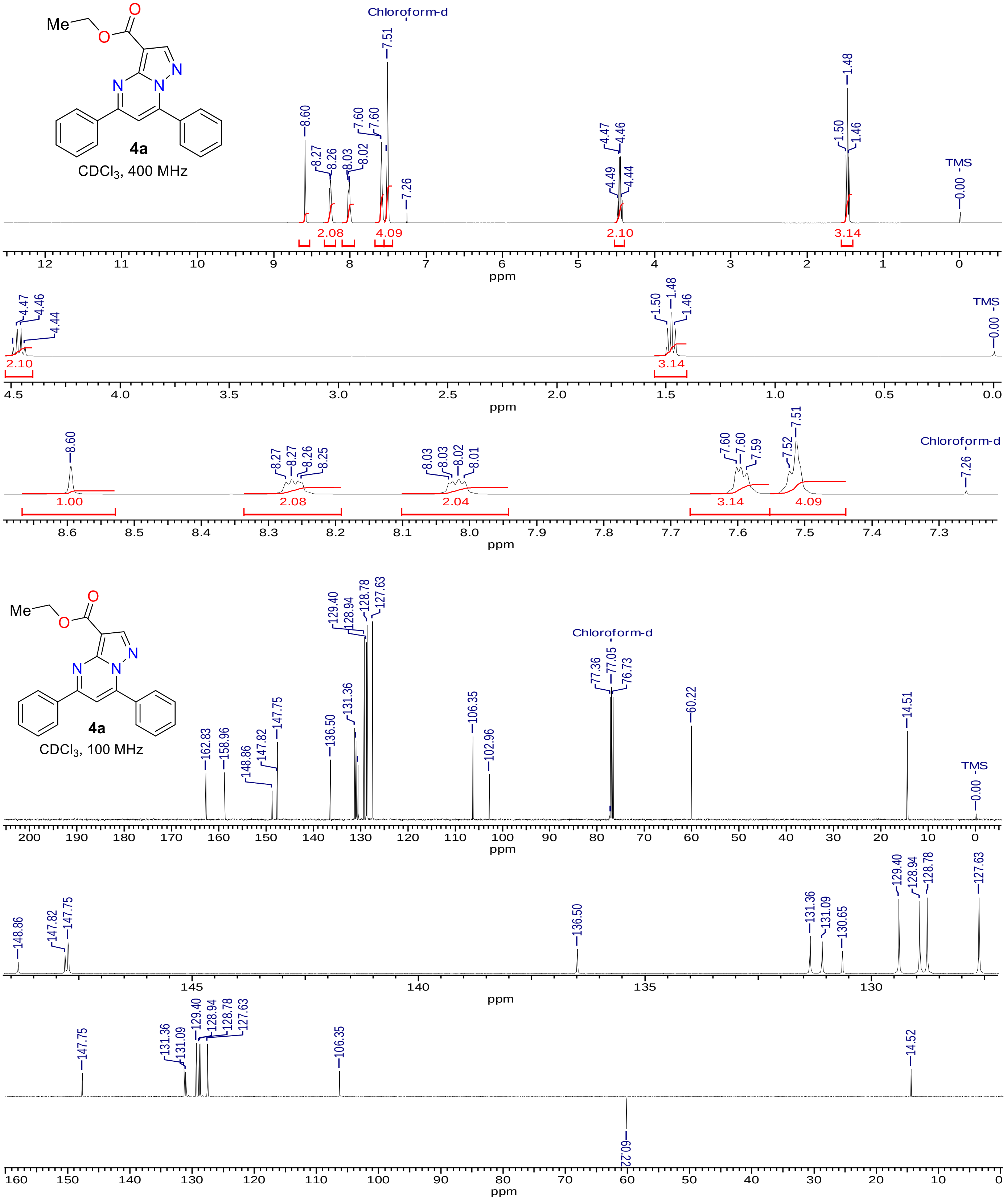

Fig. S16 ^{1}H, $^{13}C\{^{1}H\}$, and DEPT-135 NMR spectra of 3-carboethoxy-5,7-diphenylpyrazolo[1,5-*a*]pyrimidine (**4a**)

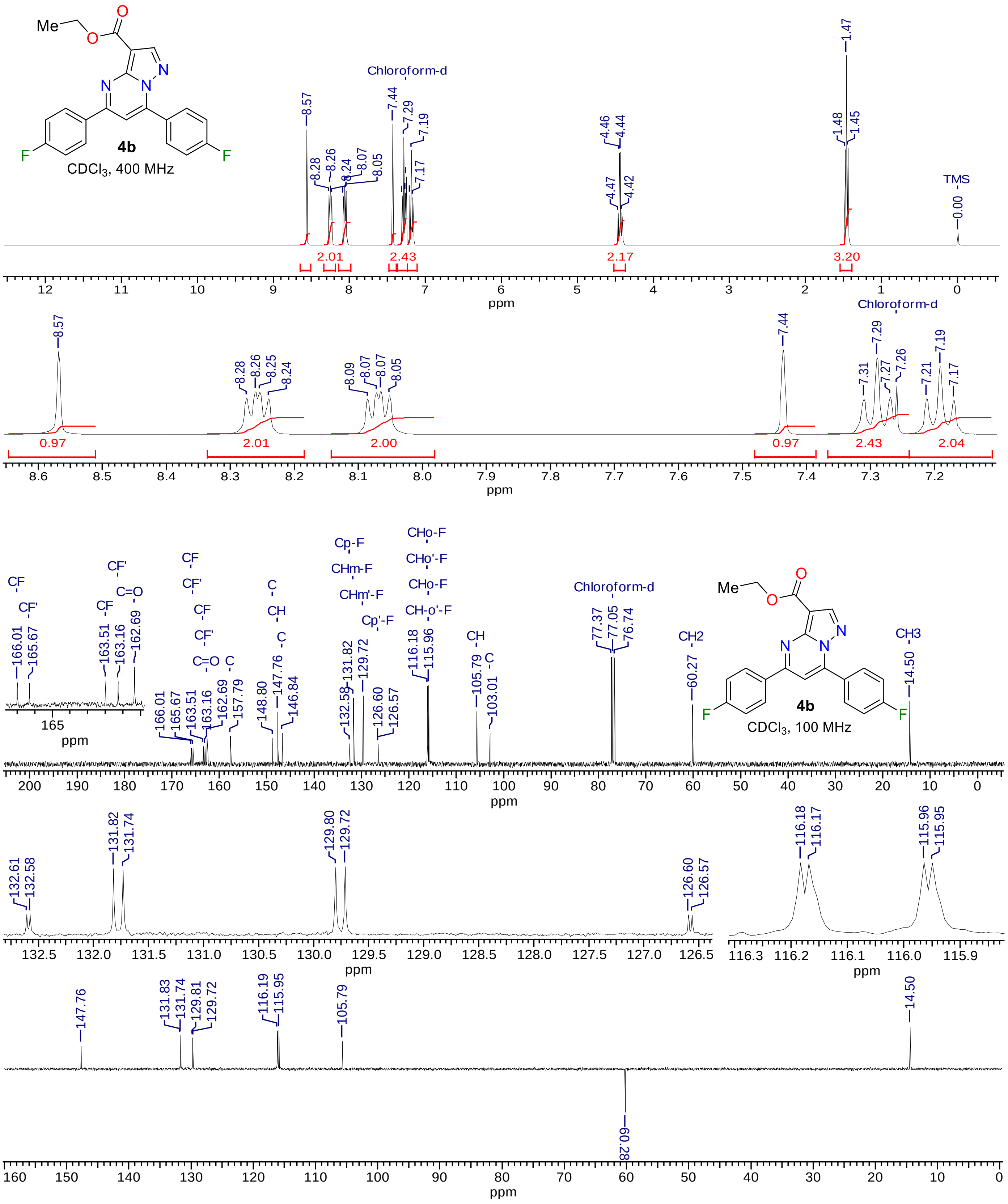

Fig. S17 ^{1}H, ^{13}C{^{1}H}, and DEPT-135 NMR spectra of 3-carboethoxy-5,7-bis(4-fluorophenyl)pyrazolo[1,5-*a*]pyrimidine (**4b**)

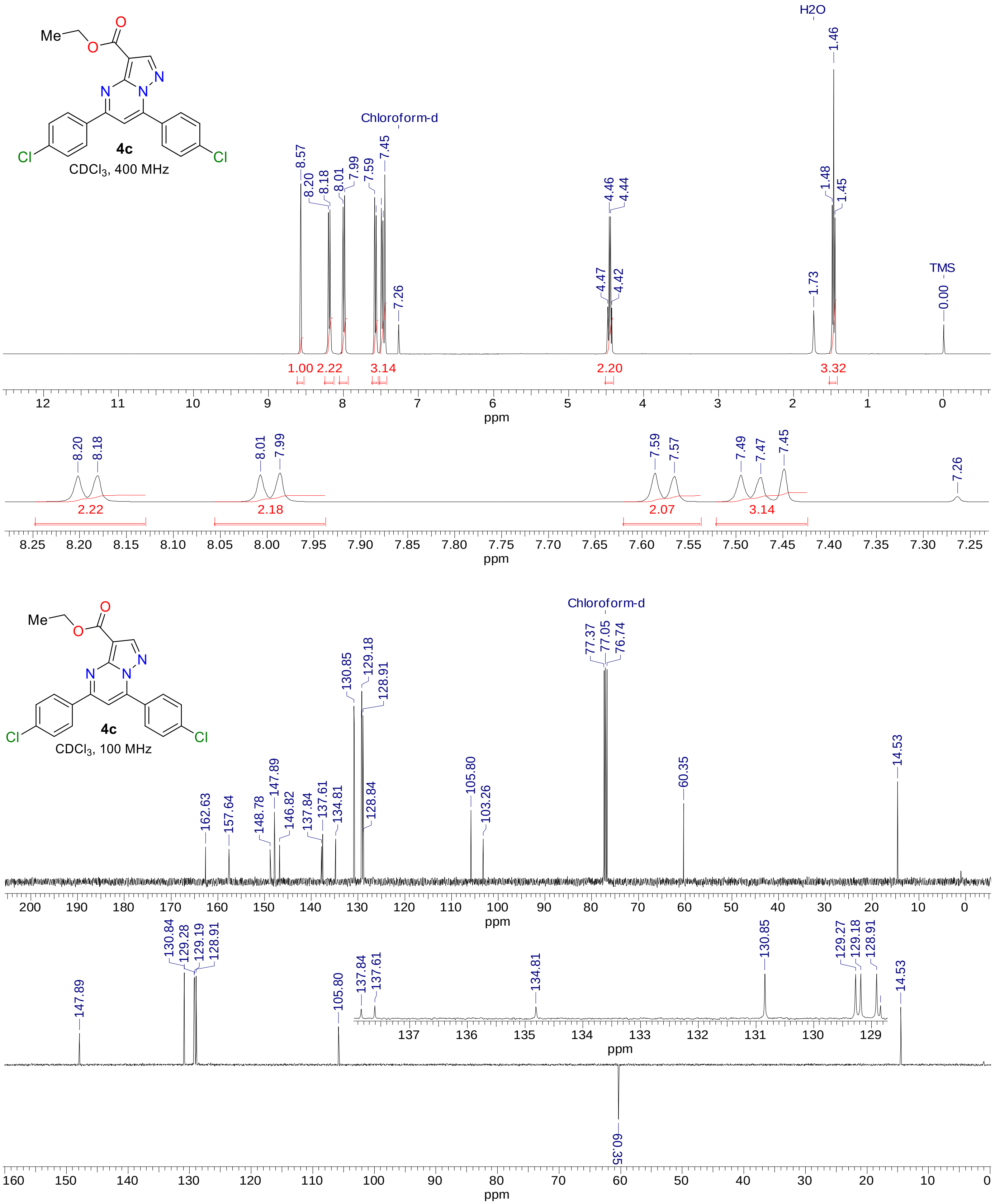

Fig. S18 ^{1}H, $^{13}C\{^{1}H\}$, and DEPT-135 NMR spectra of 3-carboethoxy-5,7-bis(4-chlorophenyl)pyrazolo[1,5-*a*]pyrimidine (**4c**)

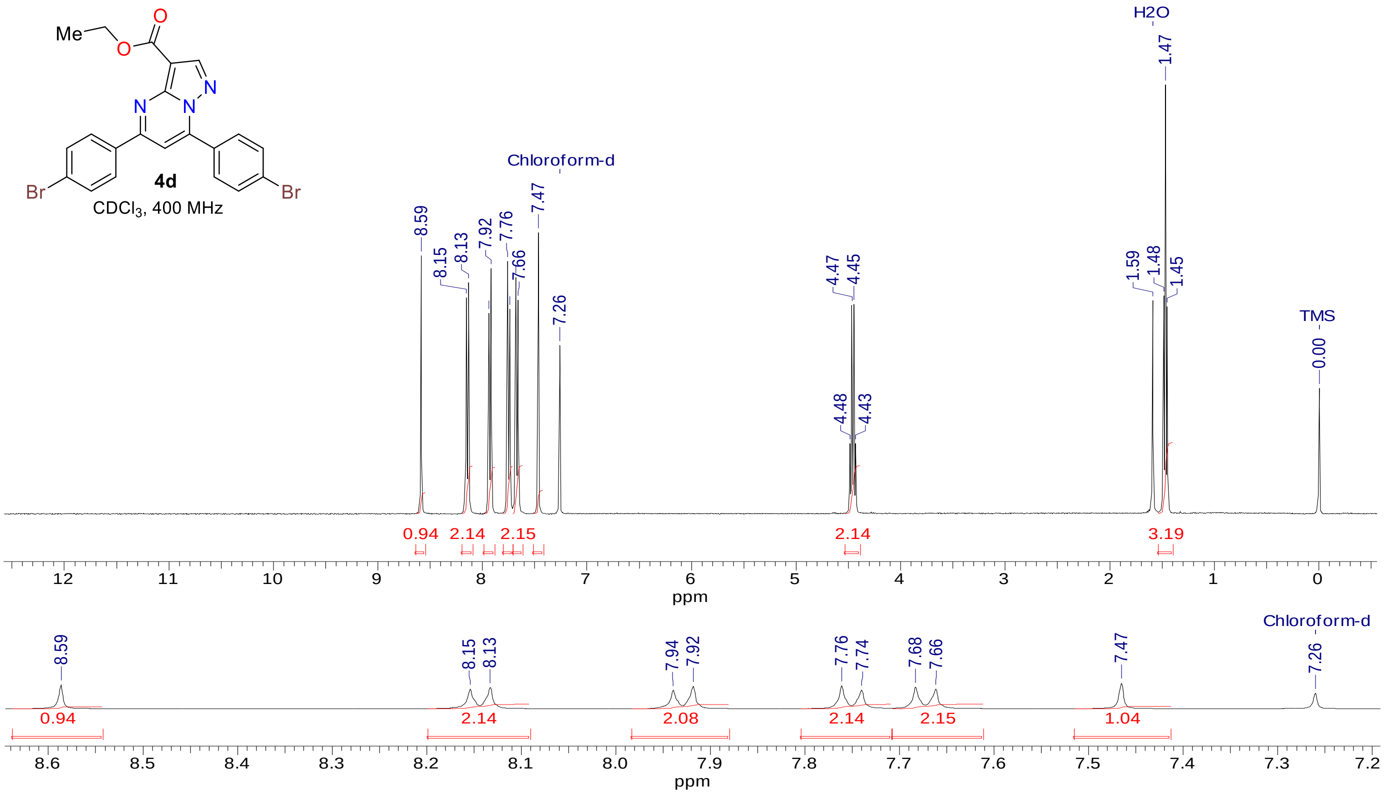

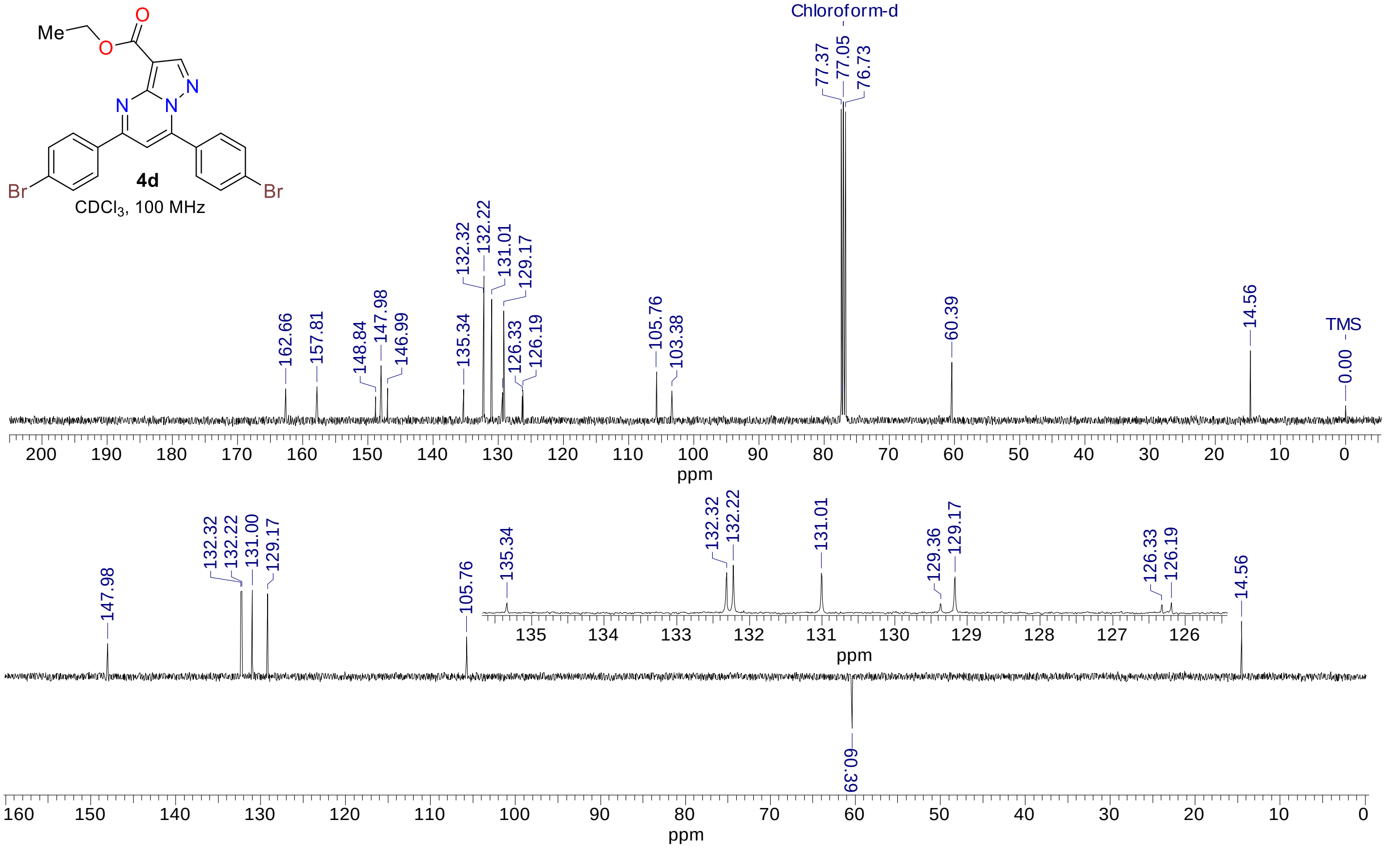

Fig. S19 ^{1}H, ^{13}C{^{1}H}, and DEPT-135 NMR spectra of 3-carboethoxy-5,7-bis(4-bromophenyl)pyrazolo[1,5-*a*]pyrimidine (**4d**)

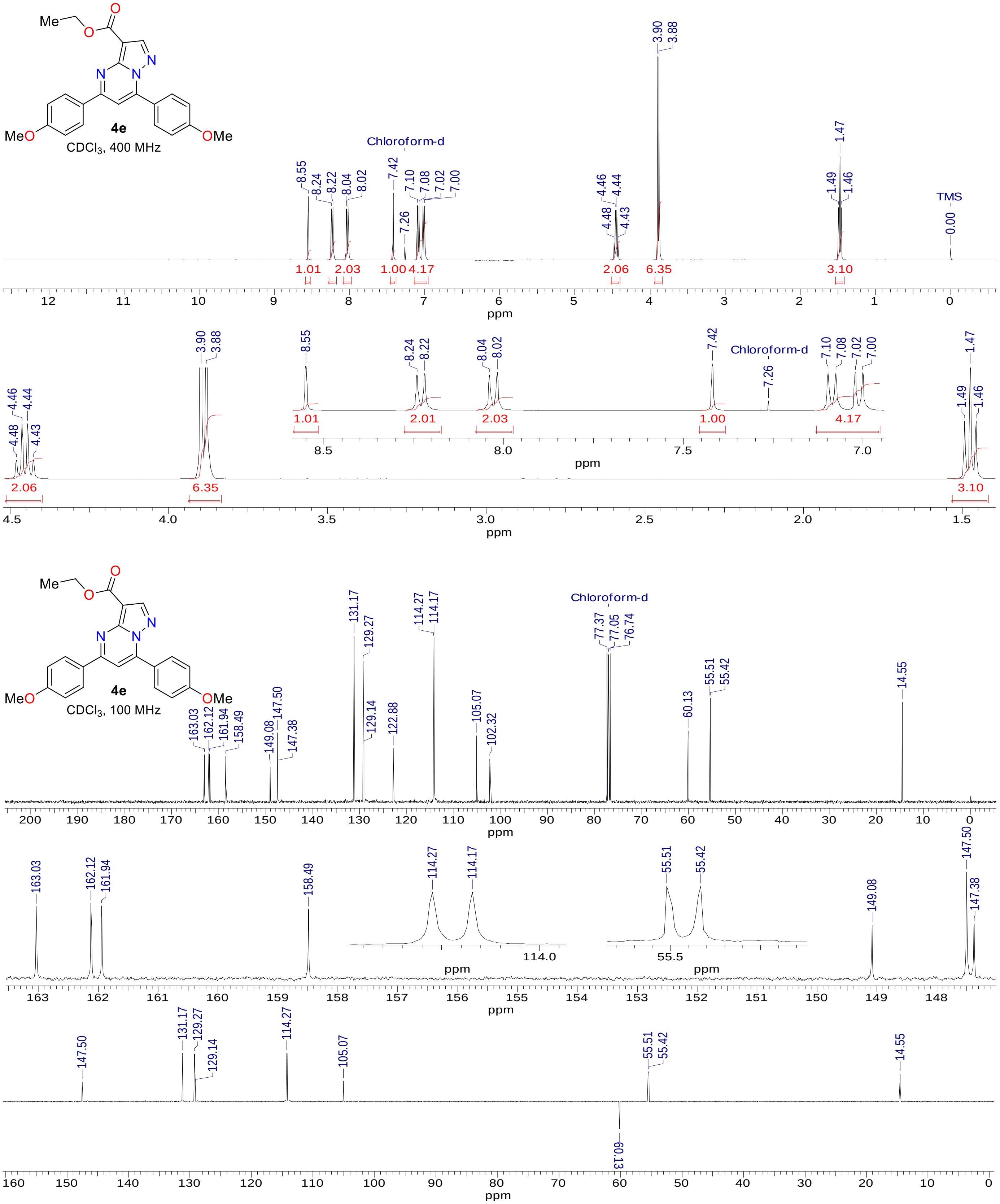

Fig. S20 ^{1}H, $^{13}C\{^{1}H\}$, and DEPT-135 NMR spectra of 3-carboethoxy-5,7-bis(4-methoxyphenyl)pyrazolo[1,5-*a*]pyrimidine (**4e**)

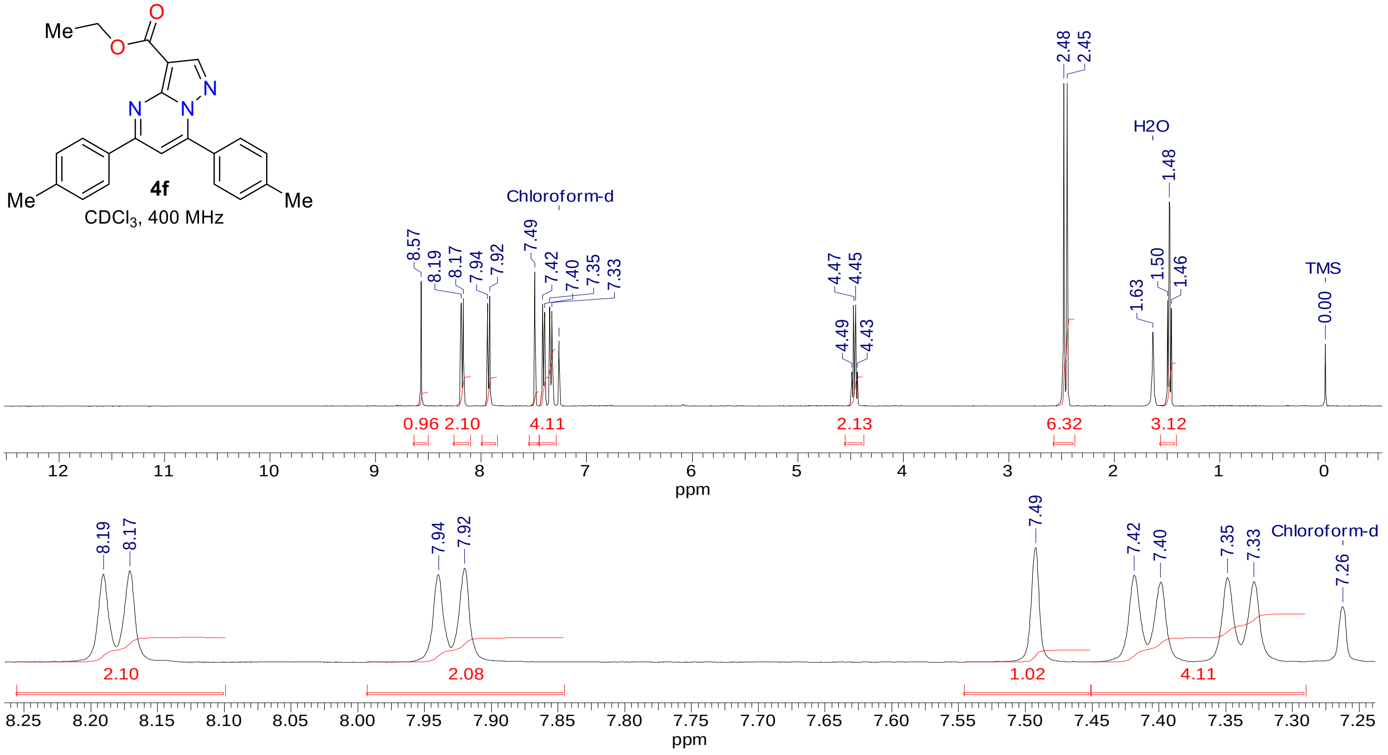

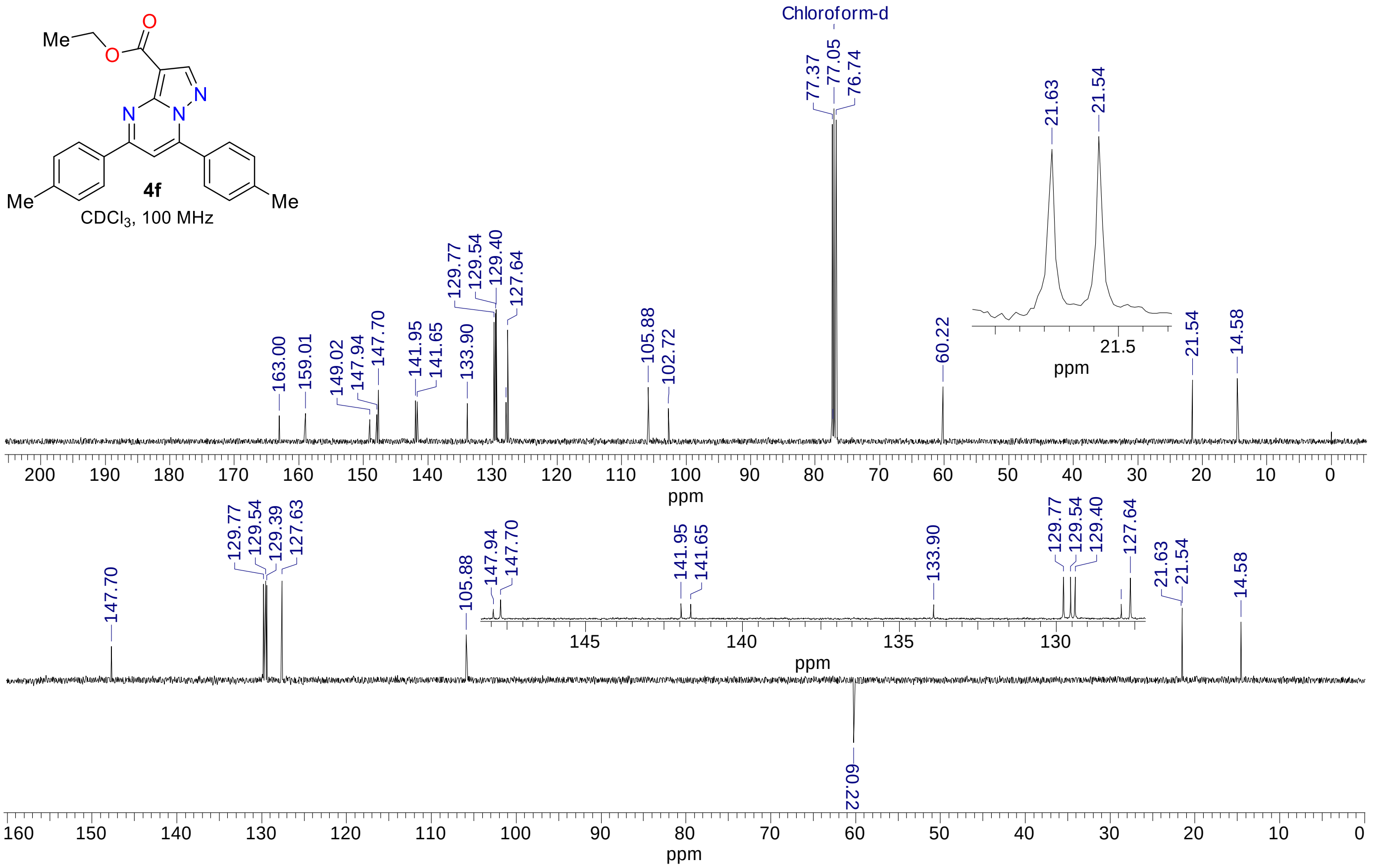

Fig. S21 ^{1}H, $^{13}C\{^{1}H\}$, and DEPT-135 NMR spectra of 3-carboethoxy-5,7-di-*p*-tolylpyrazolo[1,5-*a*]pyrimidine (**4f**)

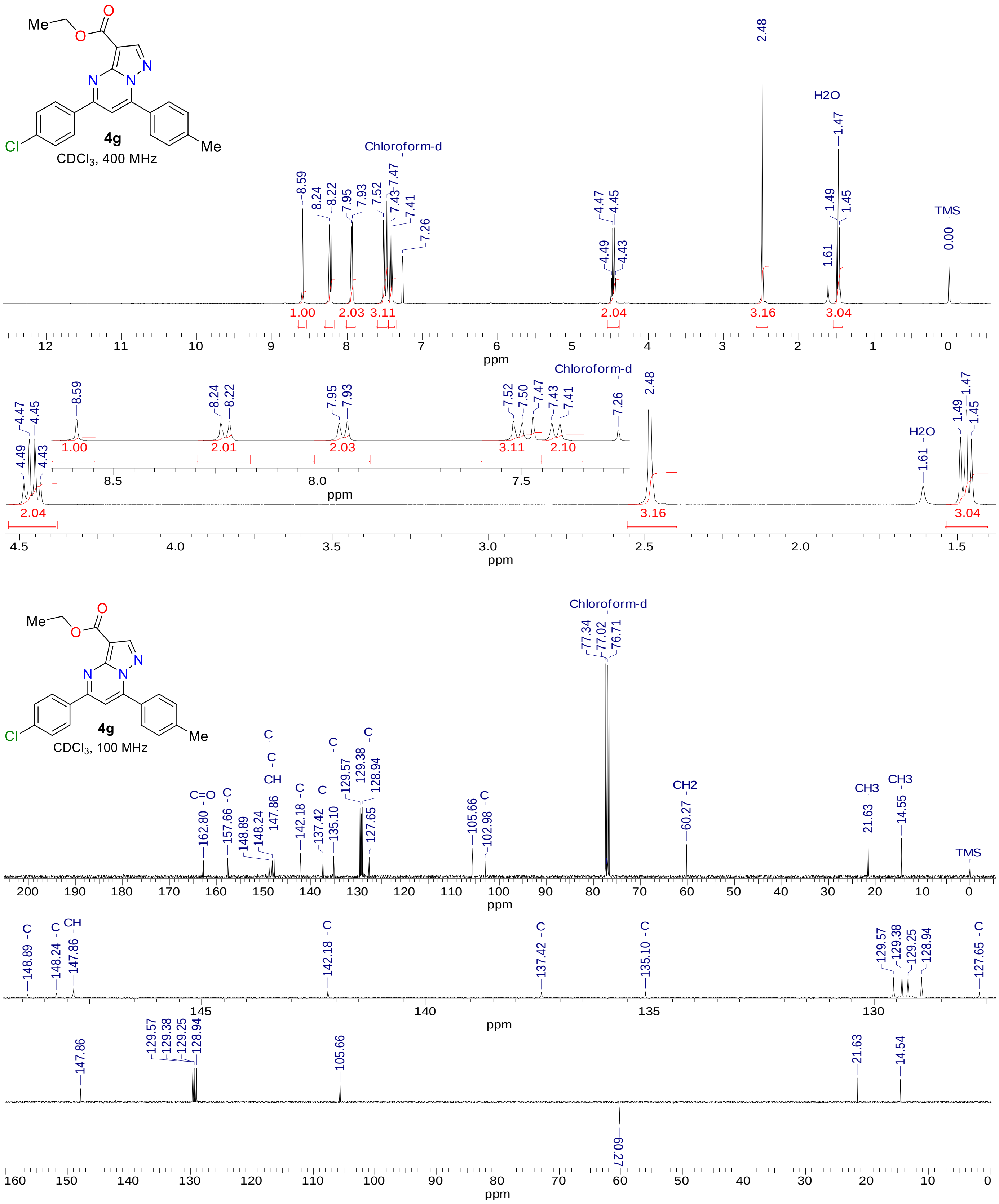

Fig. S22 ^{1}H, $^{13}C\{^{1}H\}$, and DEPT-135 NMR spectra of 3-carboethoxy-5-(4-chlorophenyl)-7-(*p*-tolyl)pyrazolo[1,5-*a*]pyrimidine (**4g**)

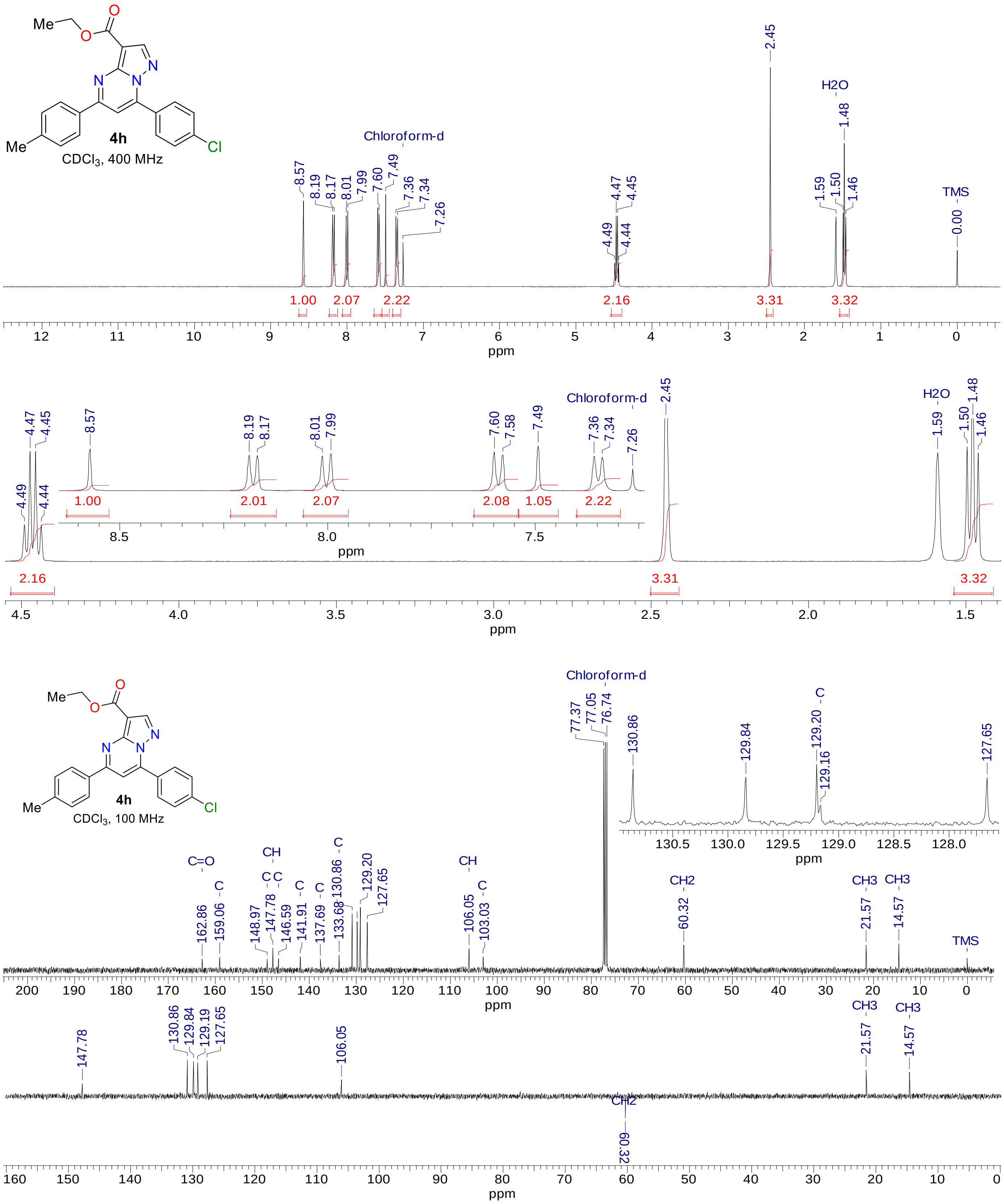

Fig. S23 ^{1}H, ^{13}C{^{1}H}, and DEPT-135 NMR spectra of 3-carboethoxy-7-(4-chlorophenyl)-5-(*p*-tolyl)pyrazolo[1,5-*a*]pyrimidine (**4h**)

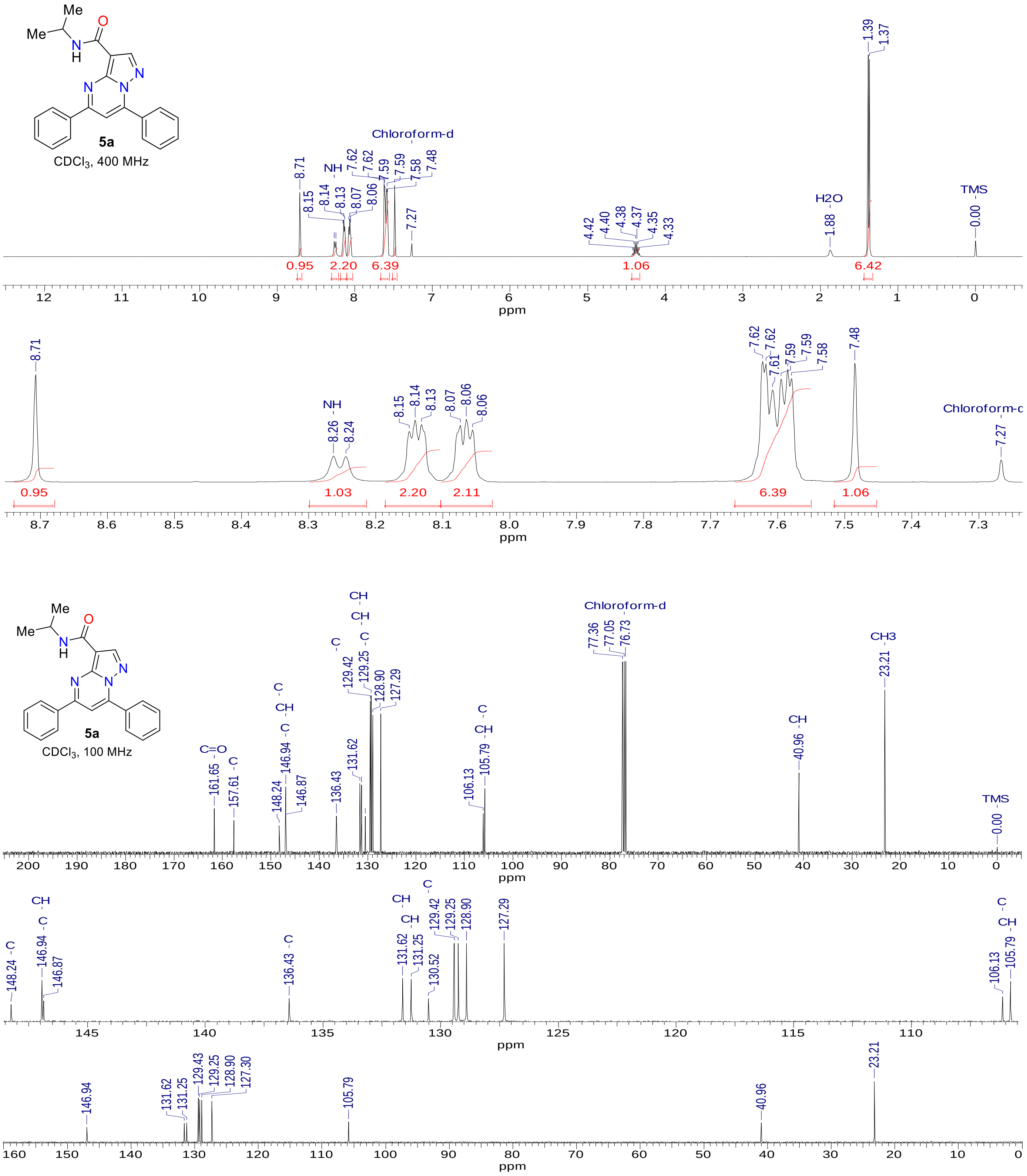

Fig. S24 ^{1}H, $^{13}C\{^{1}H\}$, and DEPT-135 NMR spectra of *N*-isopropyl-5,7-diphenylpyrazolo[1,5-*a*]pyrimidine-3-carboxamide (**5a**).

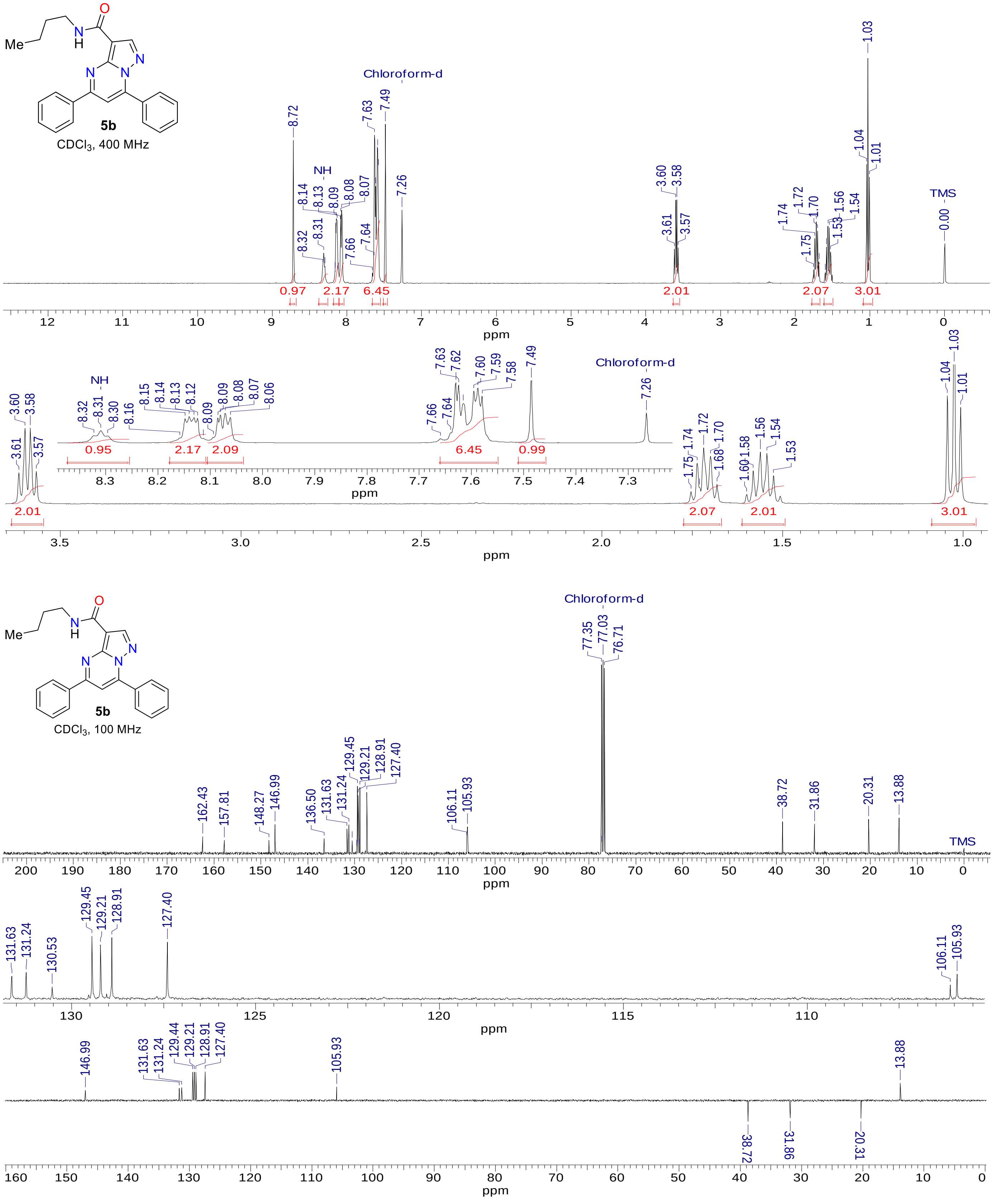

Fig. S25 ^{1}H, ^{13}C{^{1}H}, and DEPT-135 NMR spectra of *N*-(*n*-butyl)-5,7-diphenylpyrazolo[1,5-*a*]pyrimidine-3-carboxamide (**5b**).

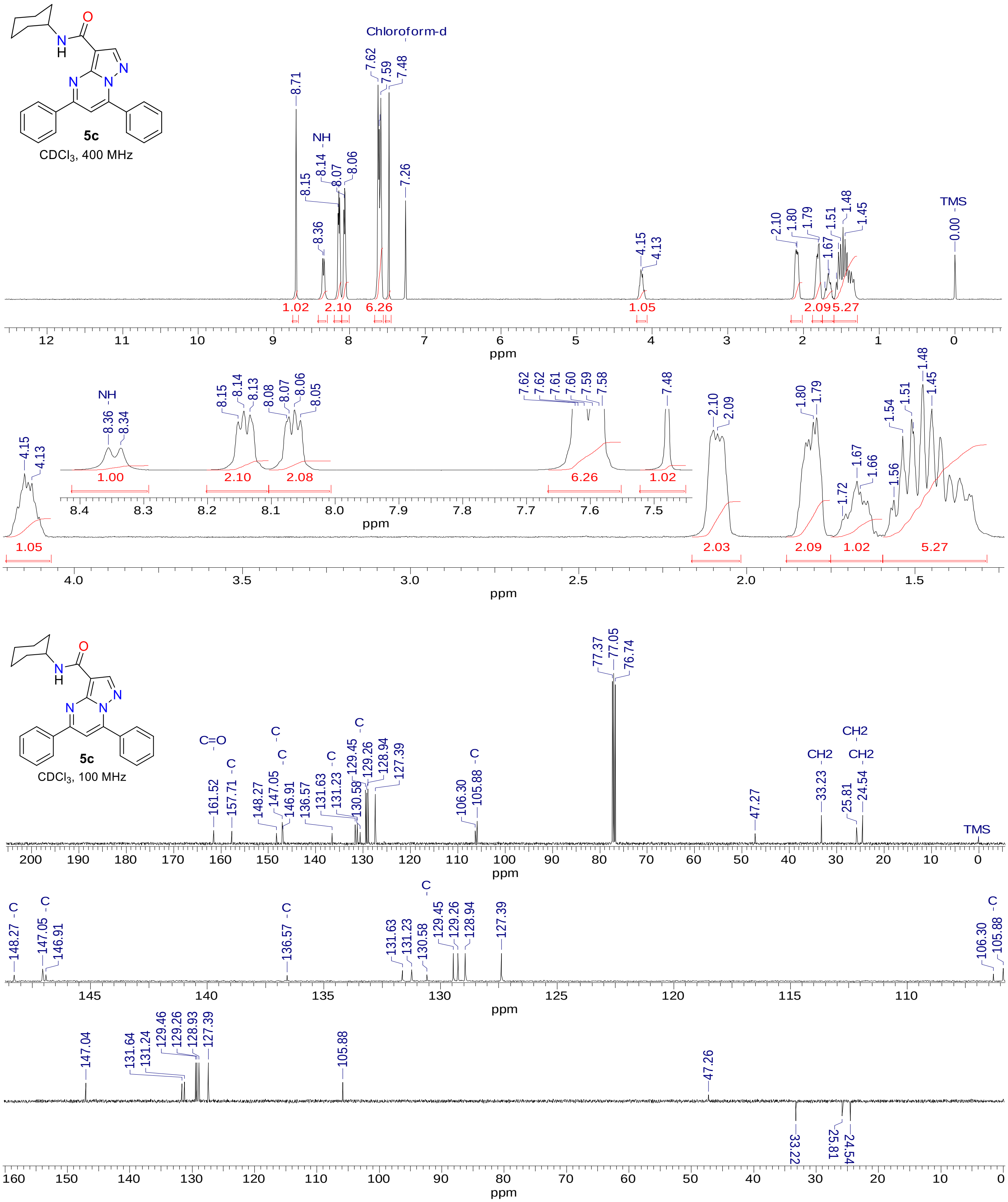

Fig. S26 ^{1}H, $^{13}C\{^{1}H\}$, and DEPT-135 NMR spectra of *N*-cyclohexyl-5,7-diphenylpyrazolo[1,5-*a*]pyrimidine-3-carboxamide (**5c**).

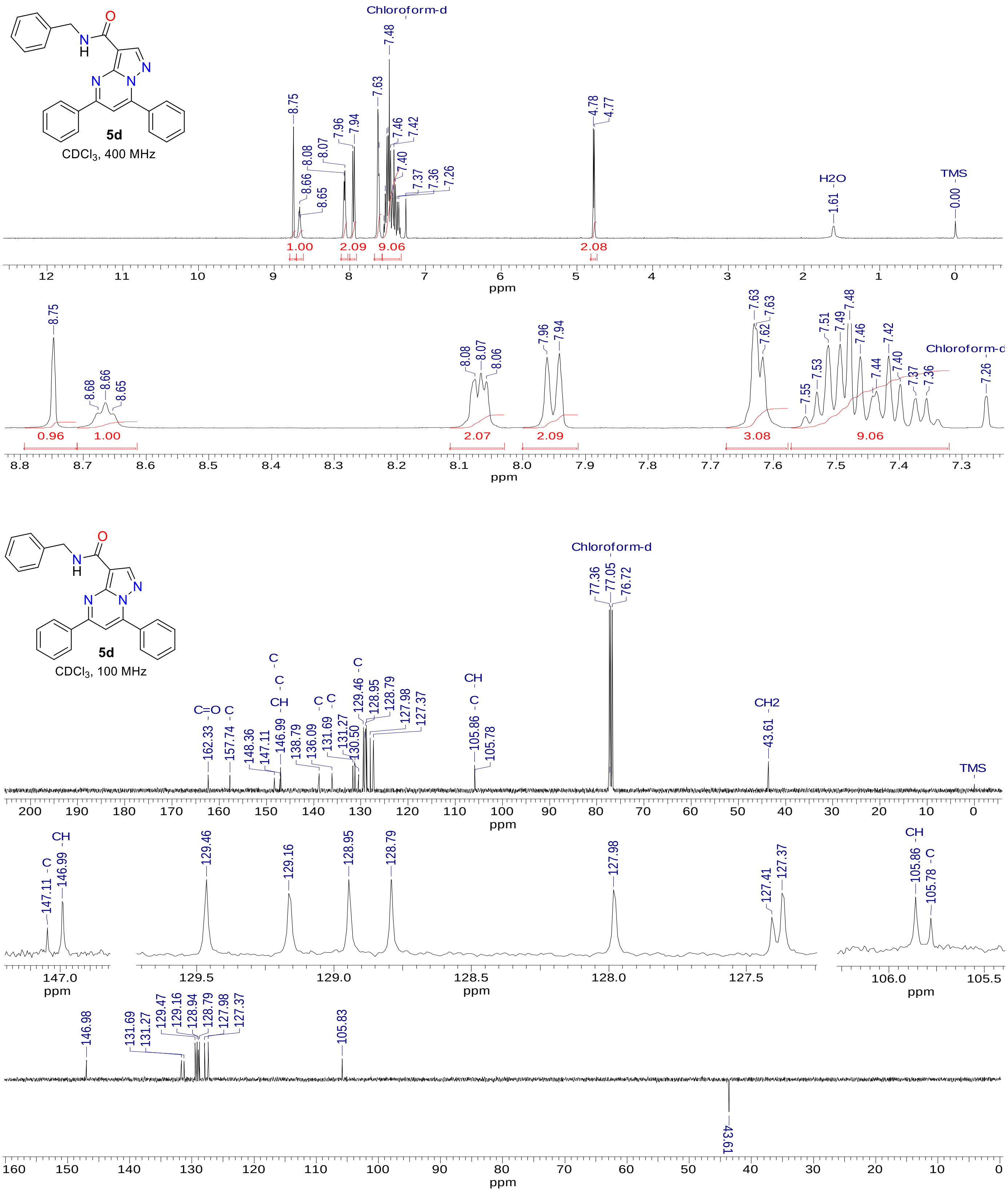

Fig. S27 ^{1}H, ^{13}C{^{1}H}, and DEPT-135 NMR spectra of *N*-benzyl-5,7-diphenylpyrazolo[1,5-*a*]pyrimidine-3-carboxamide (**5d**).

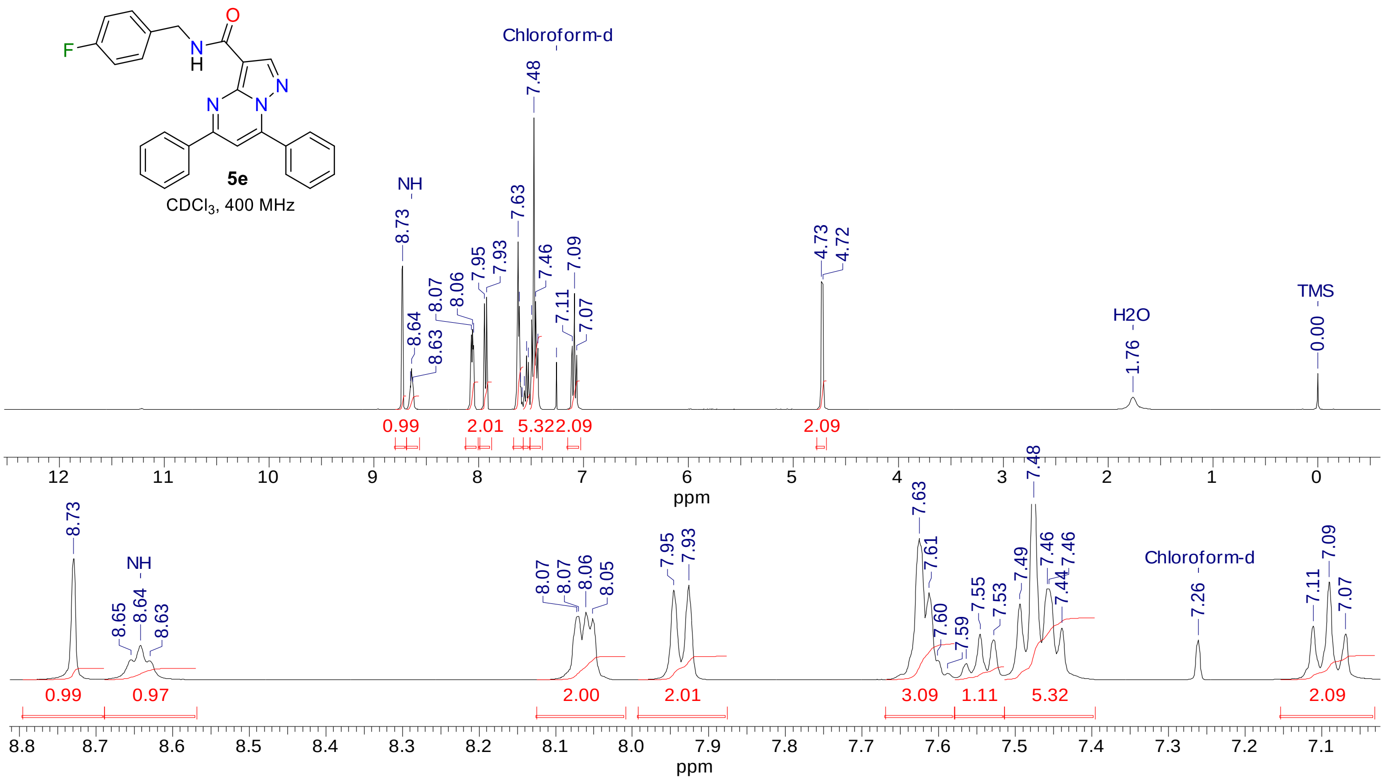

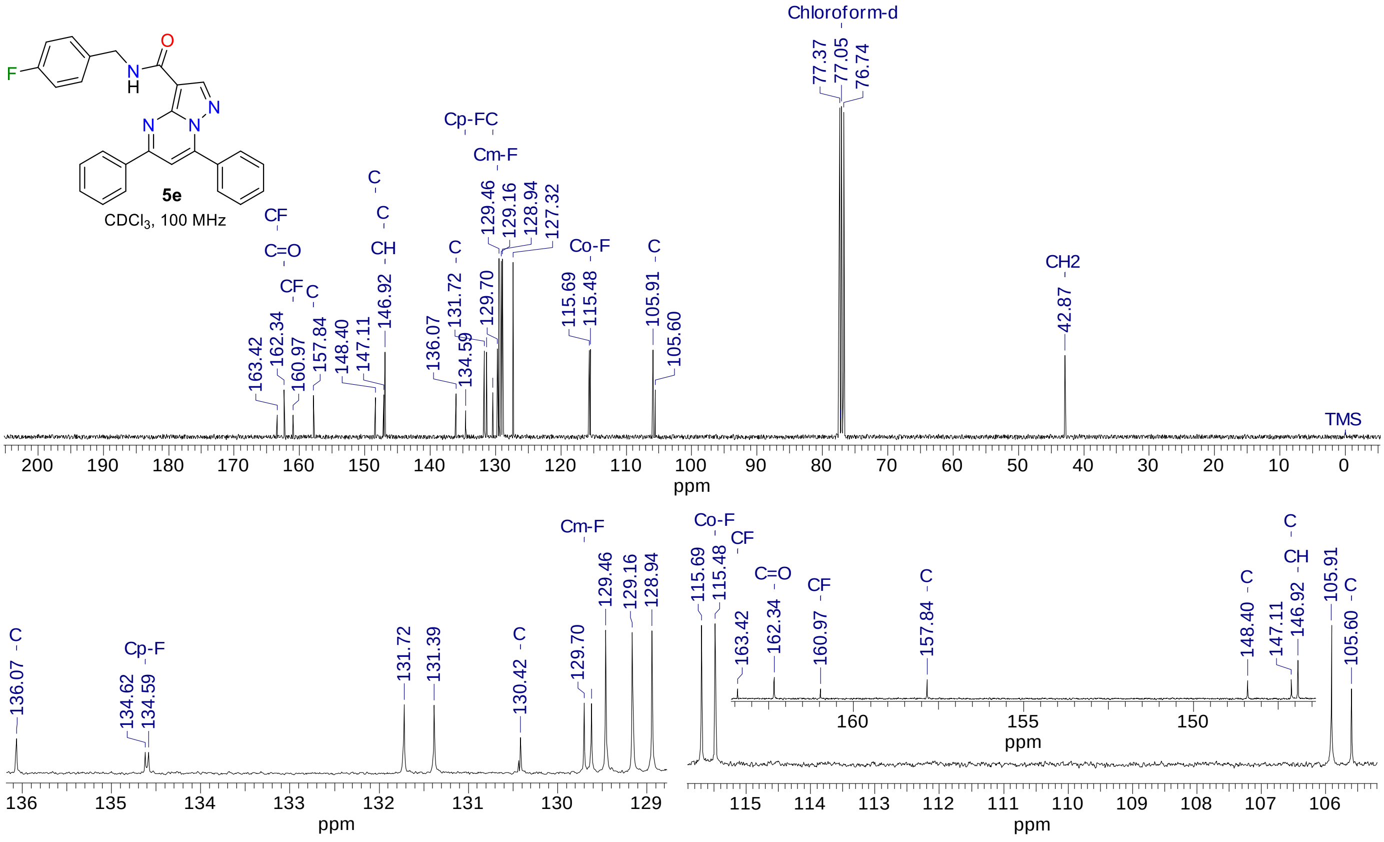

Fig. S28 ^{1}H, $^{13}C\{^{1}H\}$, and DEPT-135 NMR spectra of *N*-(4-fluorobenzyl)-5,7-diphenylpyrazolo[1,5-*a*]pyrimidine-3-carboxamide (**5e**).

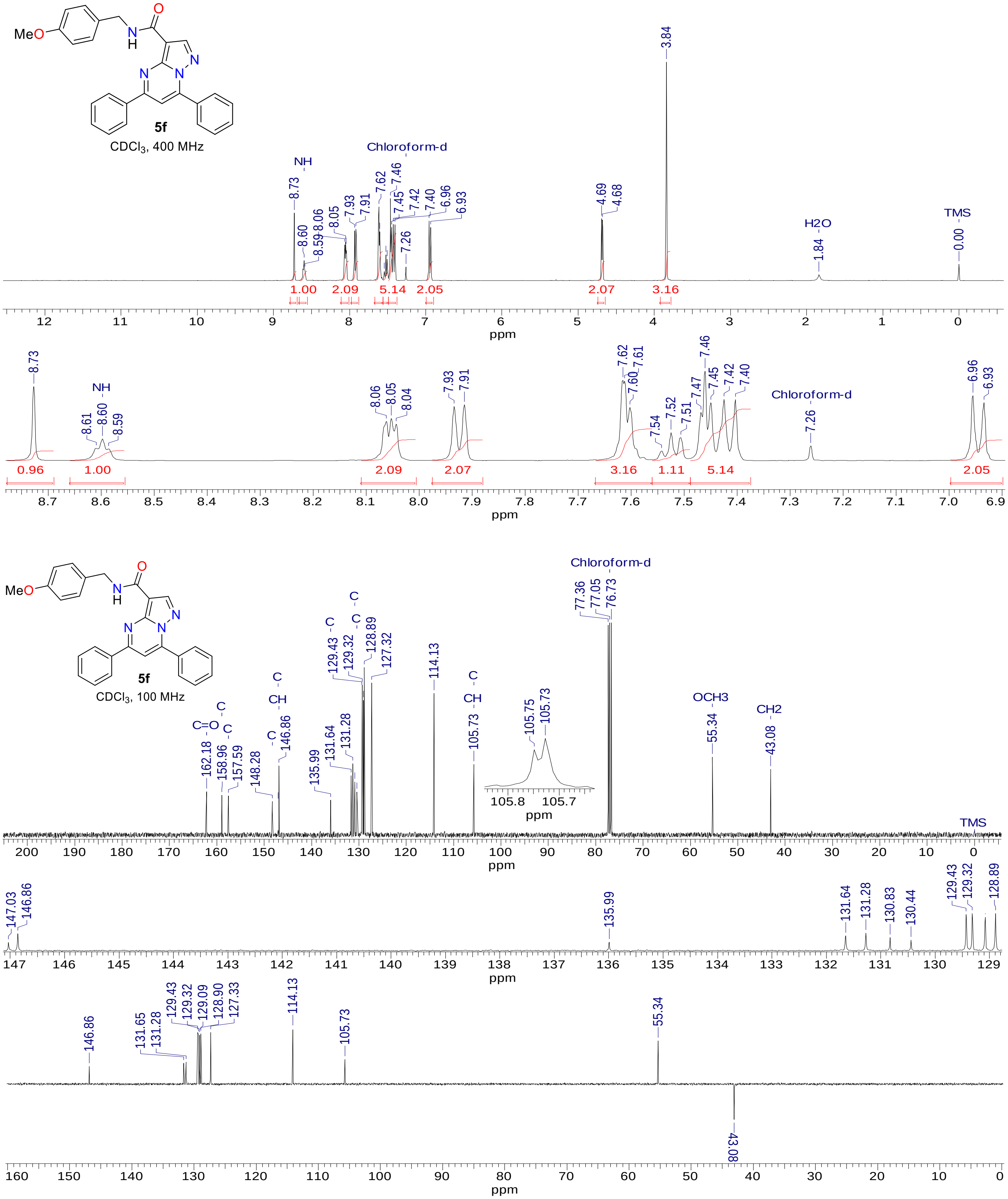

Fig. S29 ^{1}H, $^{13}C\{^{1}H\}$, and DEPT-135 NMR spectra of *N*-(4-methoxybenzyl)-5,7-diphenylpyrazolo[1,5-*a*]pyrimidine-3-carboxamide (**5f**).

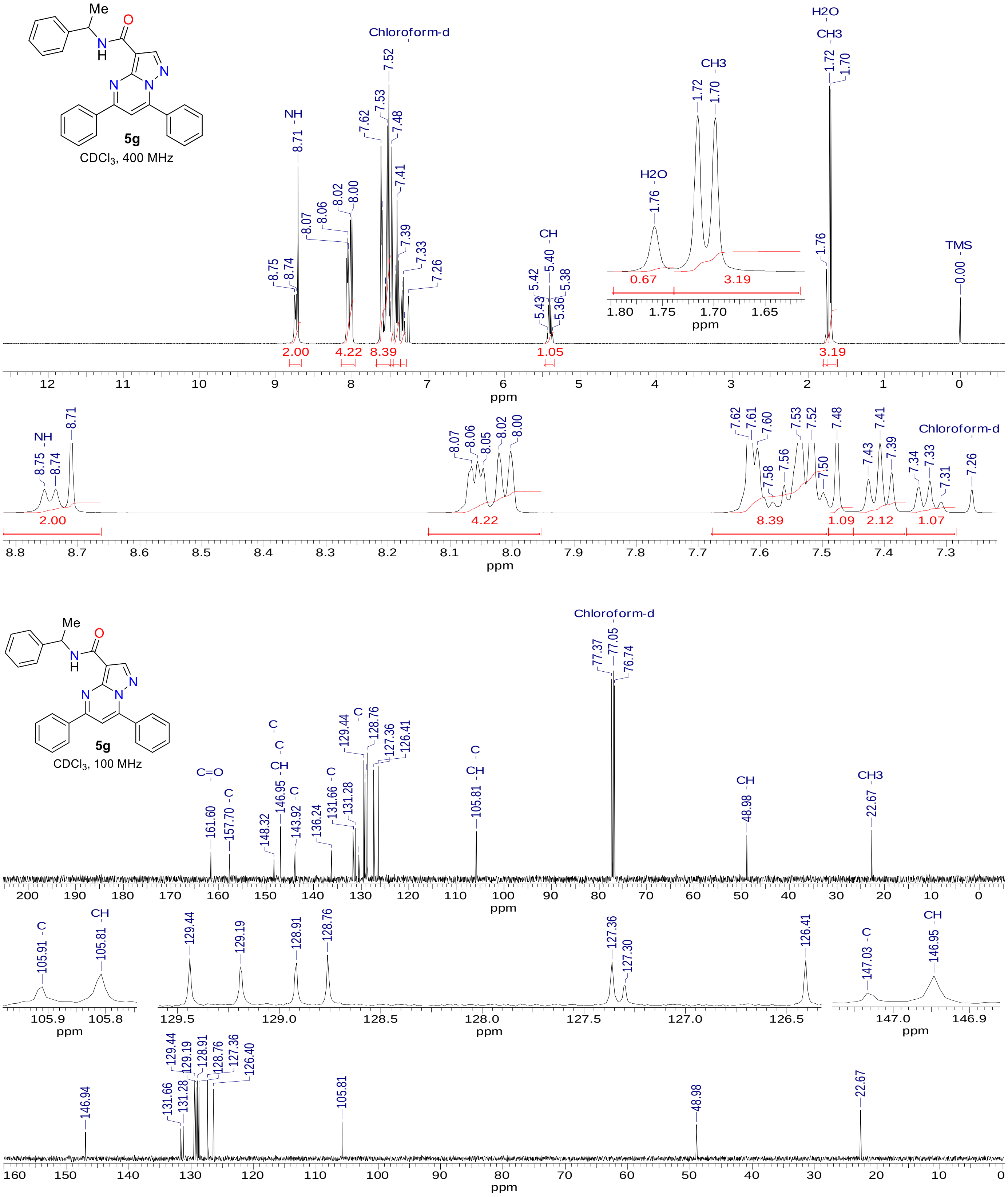

Fig. S30 ^{1}H, $^{13}C\{^{1}H\}$, and DEPT-135 NMR spectra of 5,7-diphenyl-*N*-(1-phenylethyl)pyrazolo[1,5-*a*]pyrimidine-3-carboxamide (**5g**).

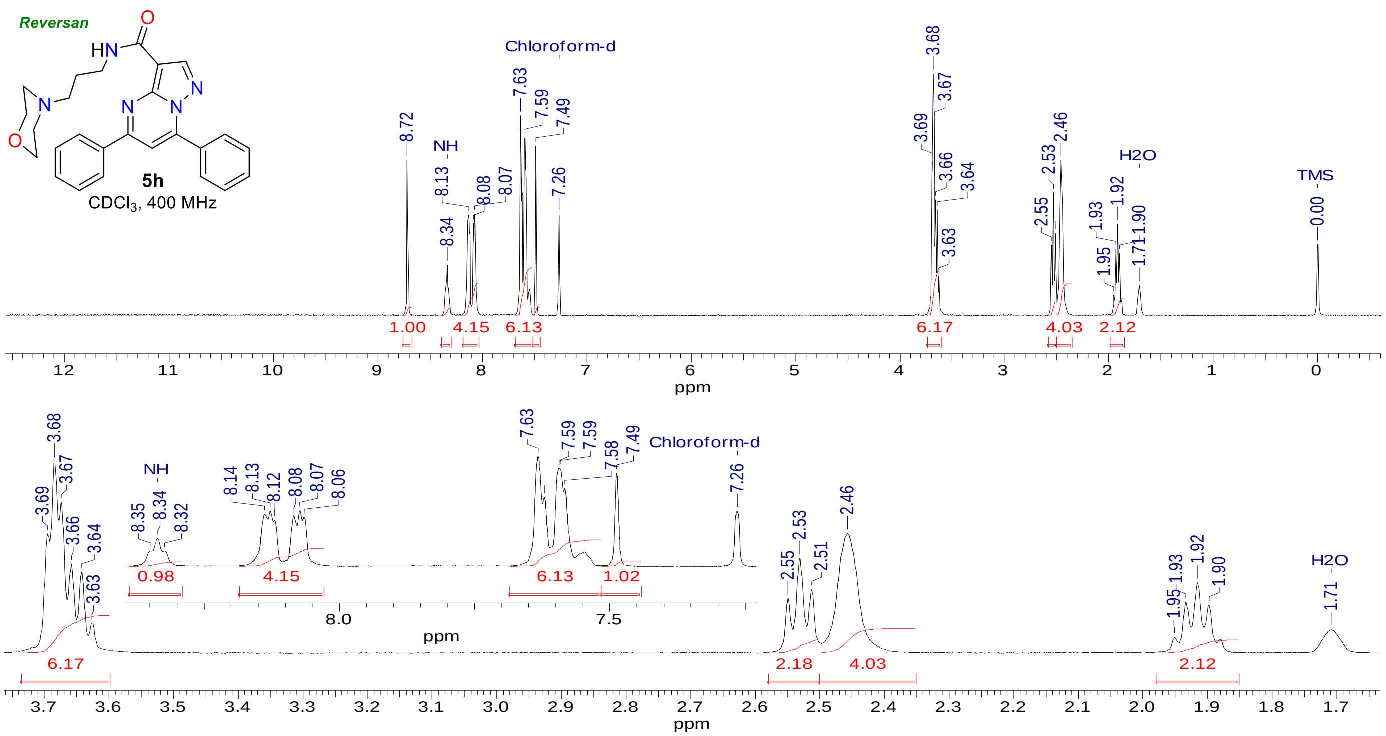

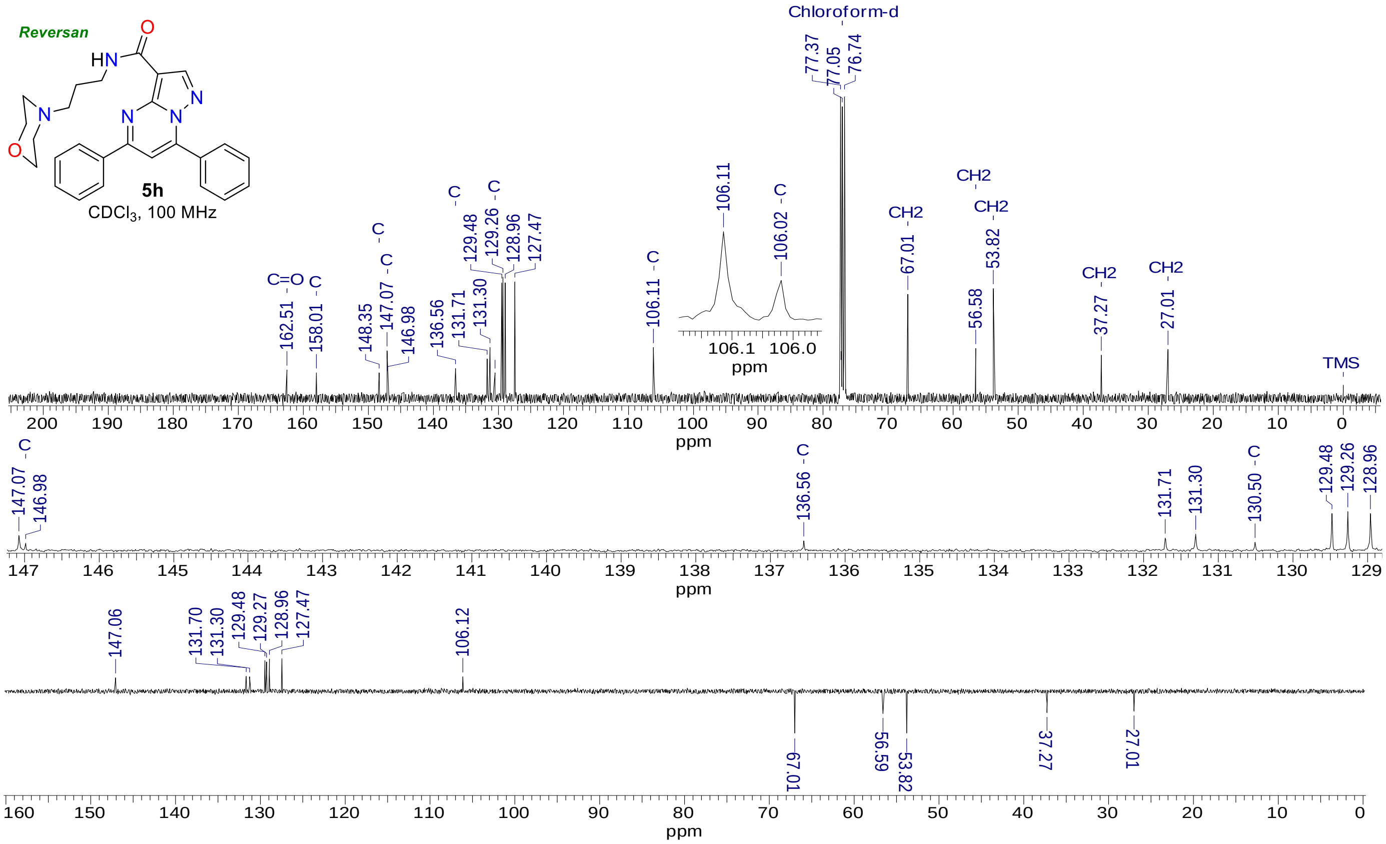

Fig. S31 ^{1}H and $^{13}C\{^{1}H\}$/DEPT-135 NMR spectra of *N*-(3-Morpholinopropyl)-5,7-diphenylpyrazolo[1,5-*a*]pyrimidine-3-carboxamide (**5h**).

4. HRMS analysis

User Chromatograms

Fragmentor Voltage 175 **Collision Energy** 0 **Ionization Mode** ESI

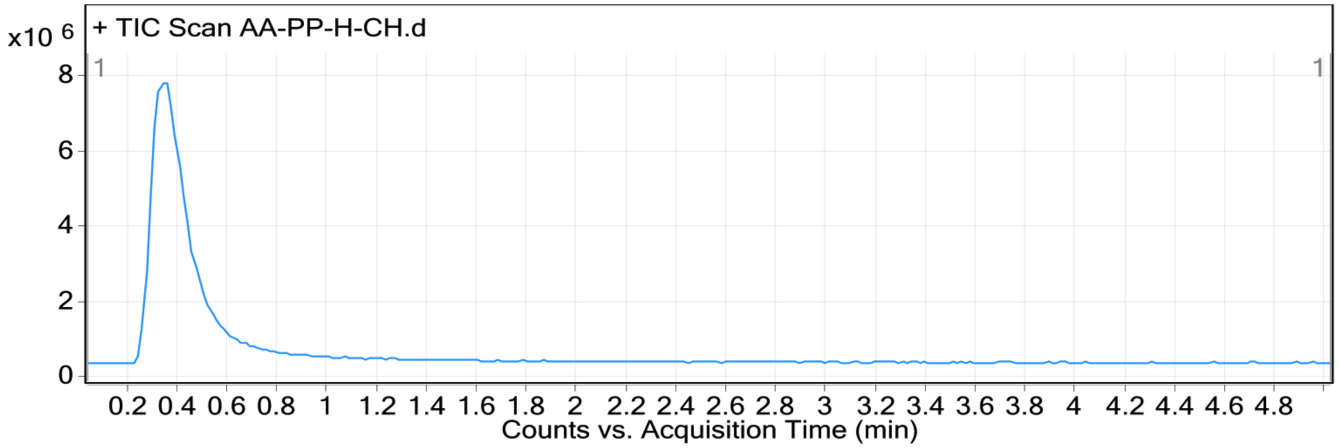

User Spectra

Fragmentor Voltage	Collision Energy	Ionization Mode
175	0	ESI

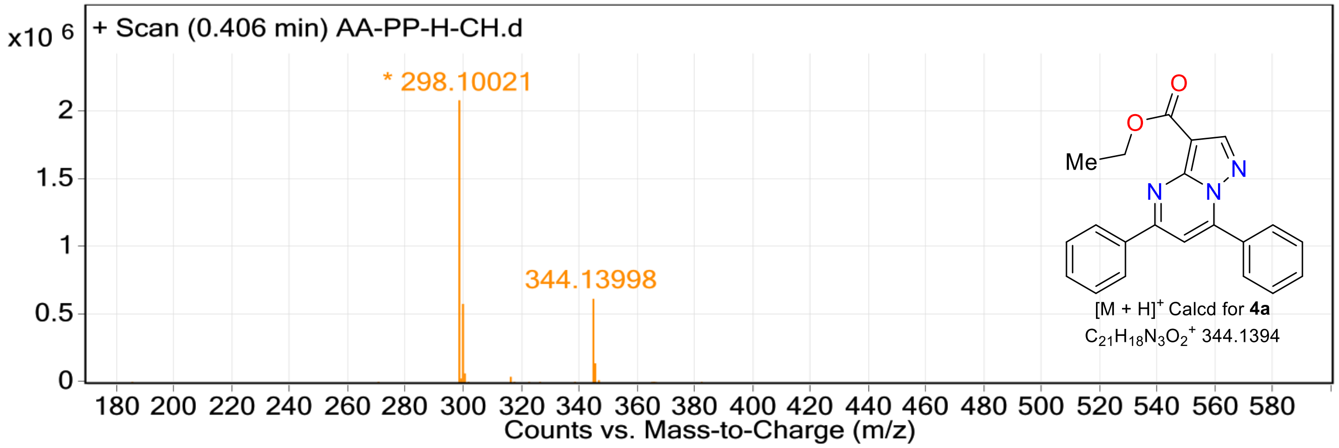

Peak List

m/z	z	Abund
298.10021	1	2084137.1
299.10115	1	591991.1
344.13998	1	625751.8
345.14256	1	151617.6

Fig. S32 HRMS analysis of compound **4a**.

User Chromatograms

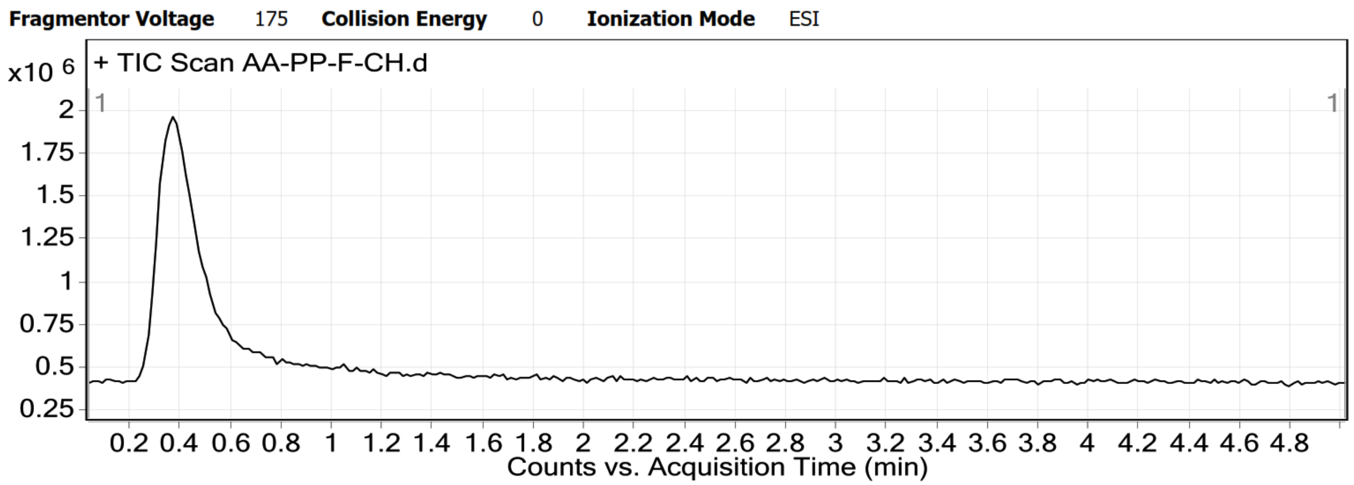

User Spectra

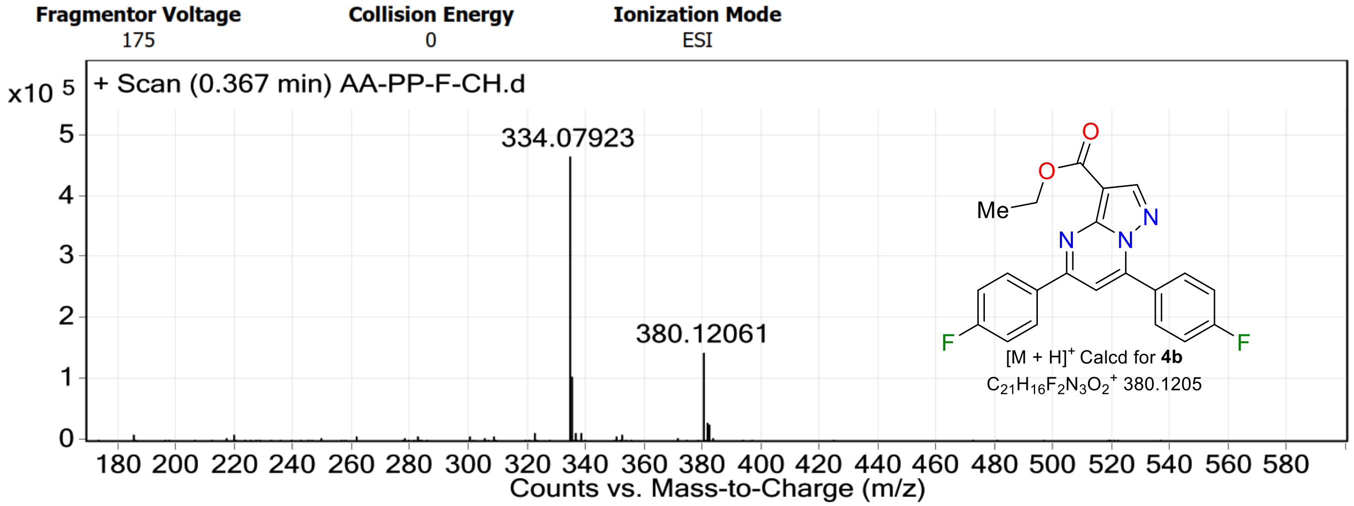

Peak List

m/z	z	Abund
167.05221		29302.2
334.07923	1	466106.1
335.08175	1	106613
380.12061	1	145056.8
381.12365	1	31243.3
382.13437	1	28293.8

Fig. S33 HRMS analysis of compound **4b**.

User Chromatograms

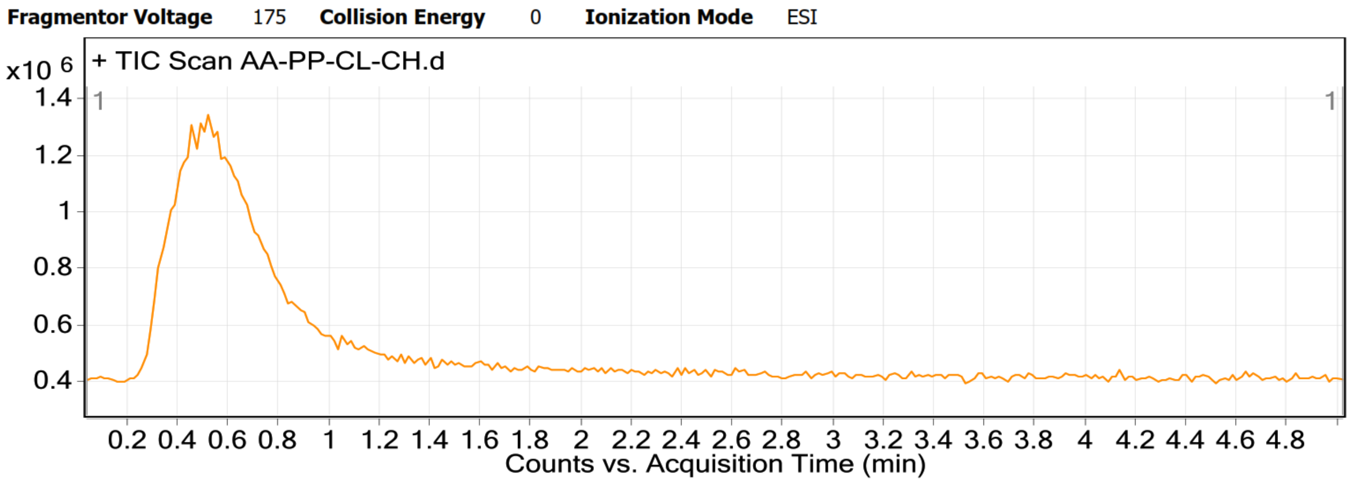

User Spectra

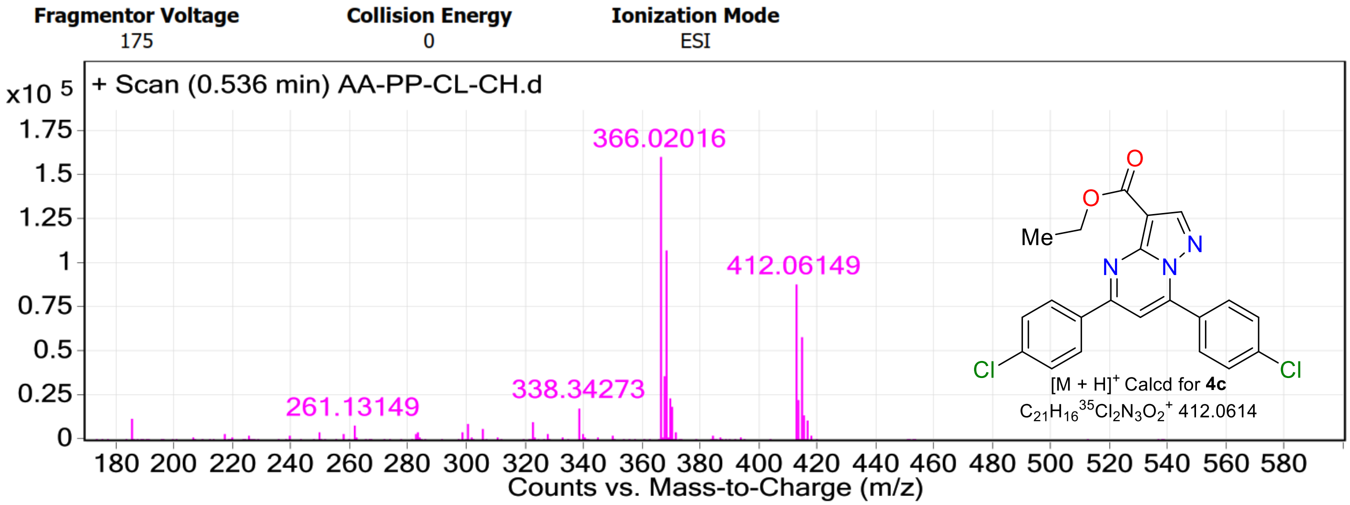

Peak List

m/z	z	Abund
167.05238		28124.7
366.02016	1	161004.4
367.0222	1	36542.1
368.01758	1	107822.6
369.01998	1	24057.1
412.06149	1	88727.6
413.06433	1	22871
414.05953	1	58413.3

Fig. S34 HRMS analysis of compound **4c**.

User Chromatograms

Fragmentor Voltage 175 **Collision Energy** 0 **Ionization Mode** ESI

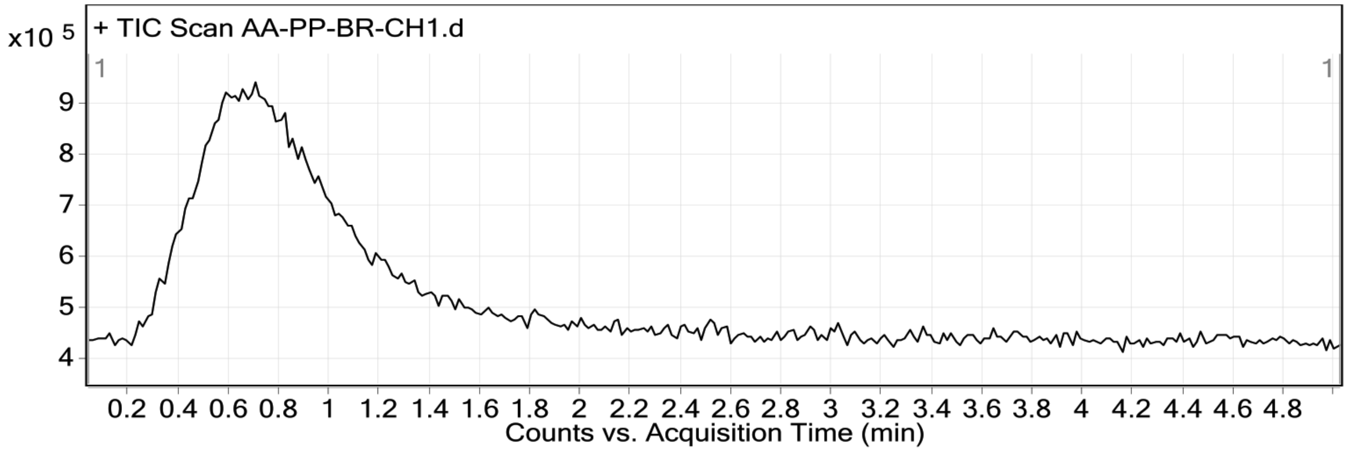

User Spectra

**Fragmentor Voltage**	**Collision Energy**	**Ionization Mode**
175	0	ESI

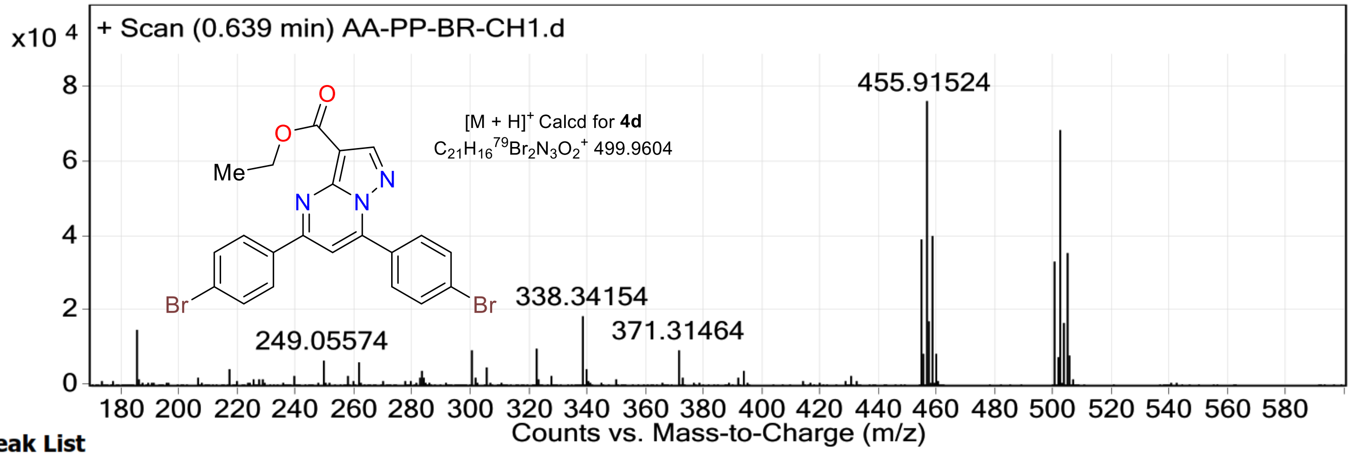

Peak List

m/z	z	Abund
158.15407		18082.9
167.05218		37059.3
338.34154	1	18906.1
453.91765	1	39391.2
455.91524	1	76488.7
456.91826	1	17399.2
457.9133	1	40228.8
499.96062	1	33264.5
501.95825	1	68745.8
503.95639	1	35633.3

Fig. S35 HRMS analysis of compound **4d**.

User Chromatograms

Fragmentor Voltage 175 **Collision Energy** 0 **Ionization Mode** ESI

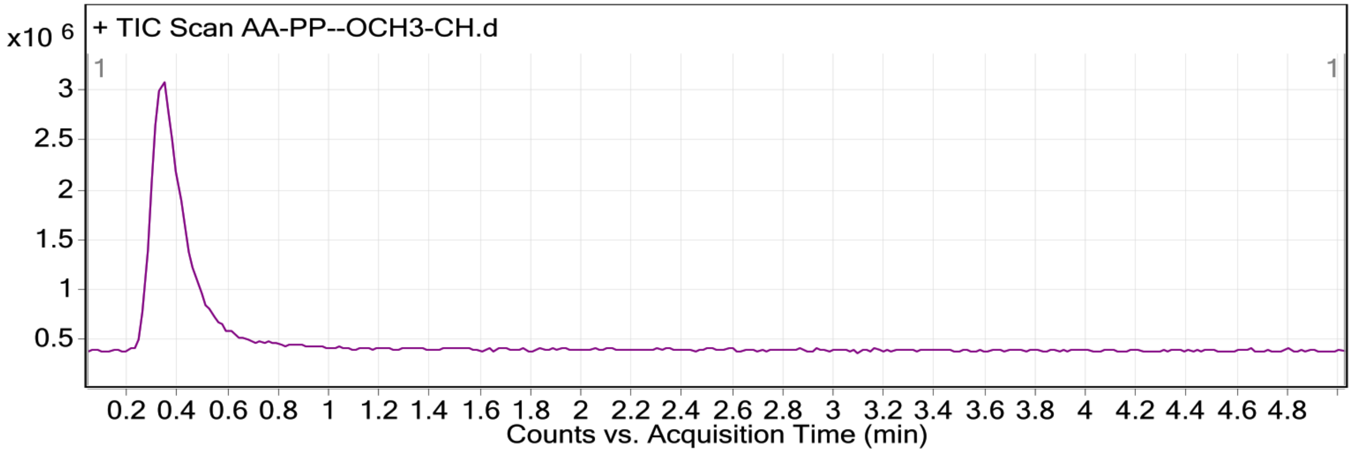

User Spectra

**Fragmentor Voltage**	**Collision Energy**	**Ionization Mode**
175	0	ESI

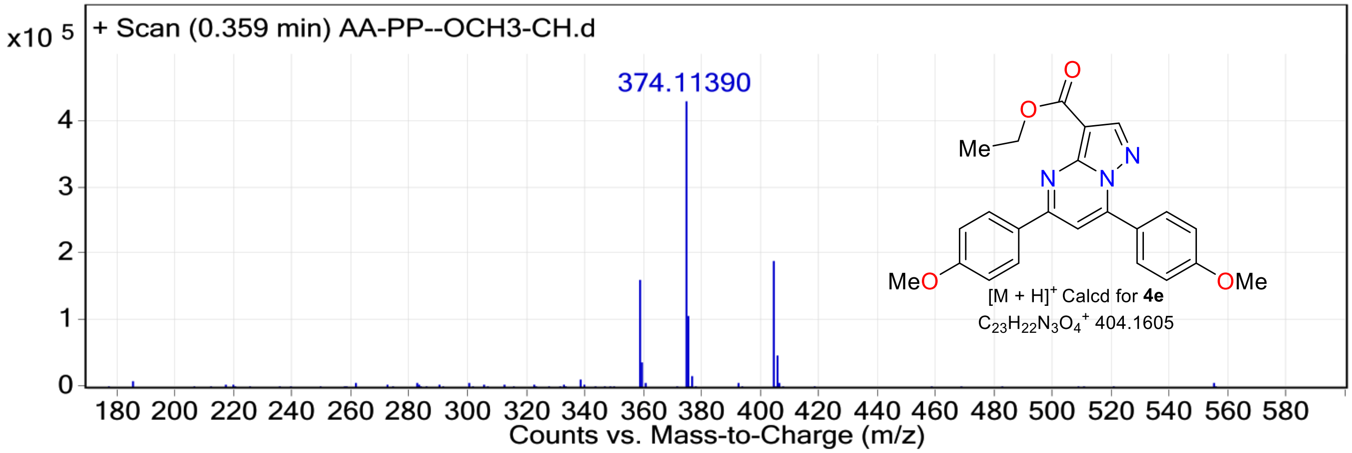

Peak List

m/z	z	Abund
358.11921	1	161141.4
374.1139	1	430593
375.11673	1	109332.6
404.16109	1	190897.2

Fig. S36 HRMS analysis of compound **4e**.

User Chromatograms

Fragmentor Voltage 175 **Collision Energy** 0 **Ionization Mode** ESI

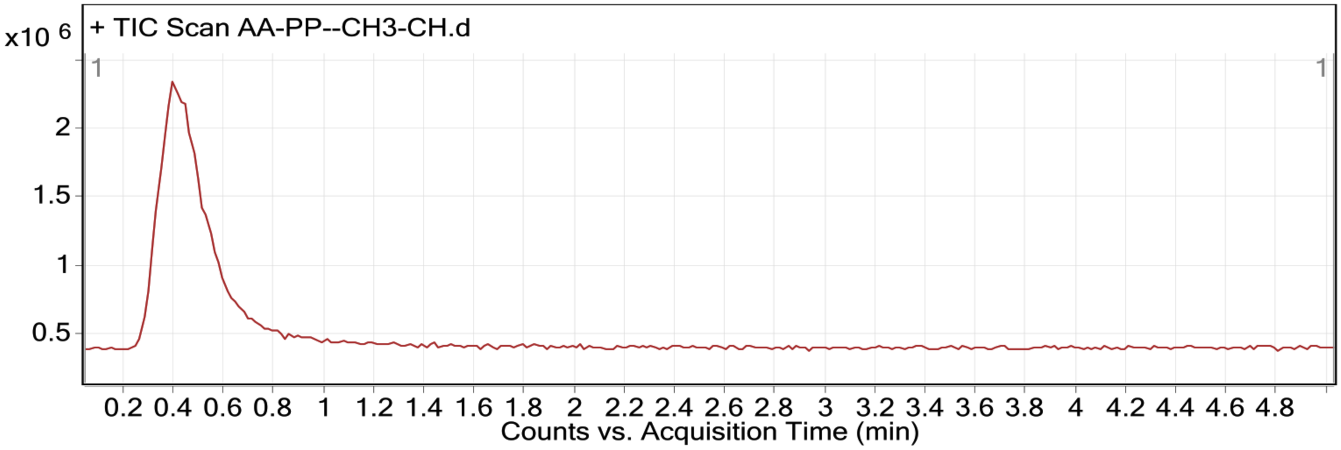

User Spectra

**Fragmentor Voltage**	**Collision Energy**	**Ionization Mode**
175	0	ESI

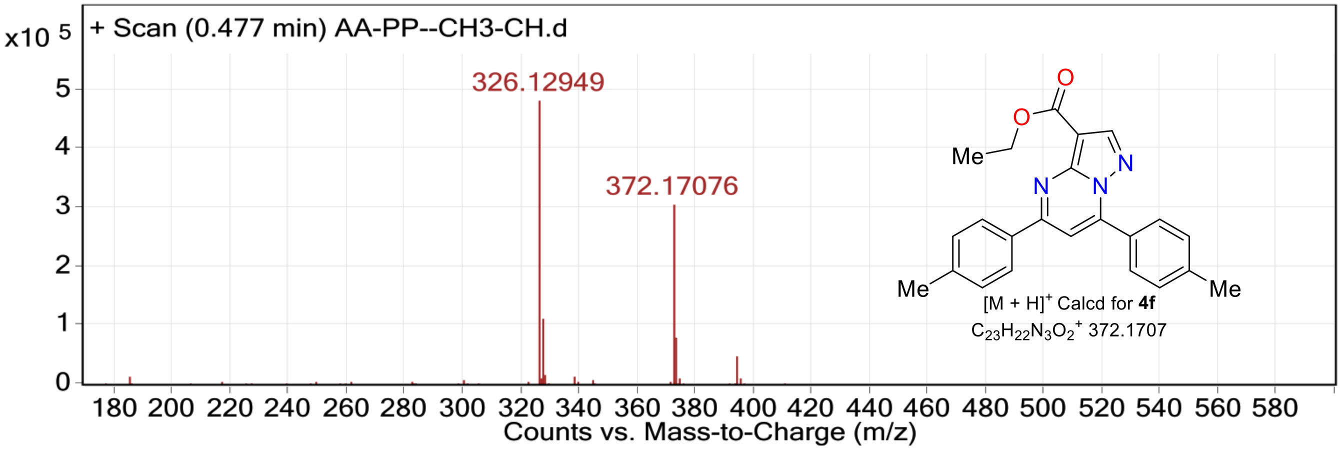

Peak List

m/z	z	Abund
167.05181		32665.5
326.12949	1	481847.4
327.13242	1	113710.6
372.17076	1	305531.1
373.17348	1	79500.5
394.15137		48119.5
765.31611	1	98810.5
766.31857	1	52650.8

Fig. S37 HRMS analysis of compound **4f**.

User Chromatograms

Fragmentor Voltage 175 **Collision Energy** 0 **Ionization Mode** ESI

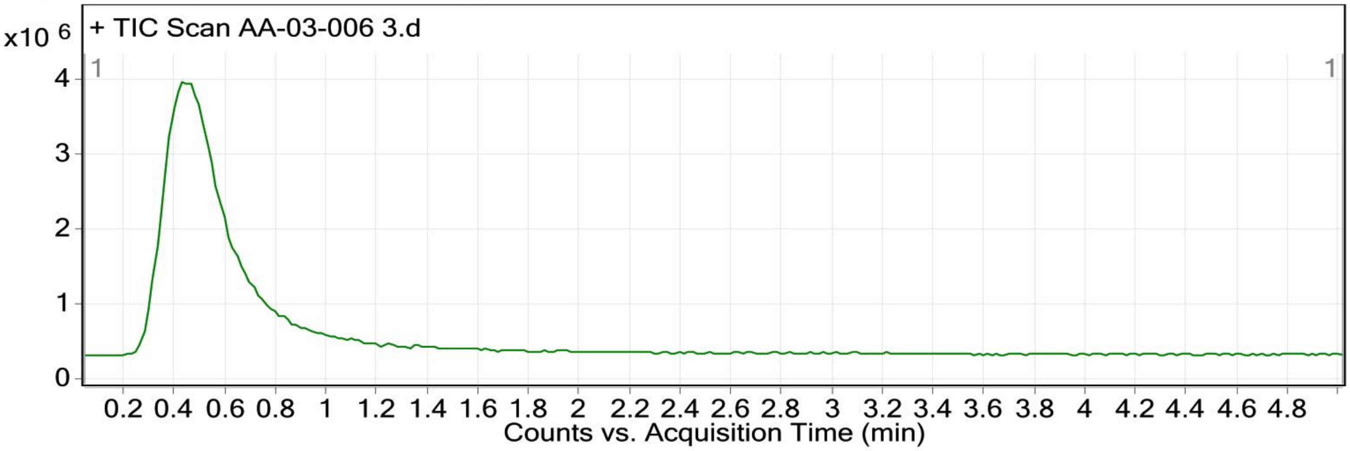

User Spectra

Fragmentor Voltage 175 **Collision Energy** 0 **Ionization Mode** ESI

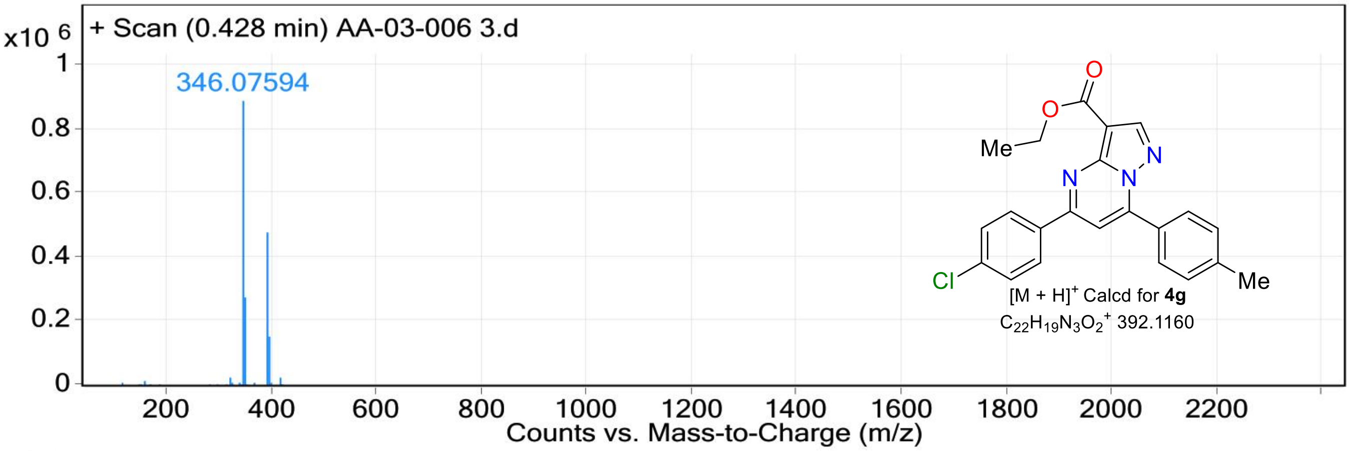

Peak List

m/z	z	Abund
346.07594	1	889233.1
346.16618		48630.3
347.07839	1	181116.3
348.07285	1	274834.8
349.07568	1	55924.4
392.11795	1	476117
393.12037	1	115735.4
394.11534	1	156757.5

Fig. S38 HRMS analysis of compound **4g**.

User Chromatograms

Fragmentor Voltage 175 **Collision Energy** 0 **Ionization Mode** ESI

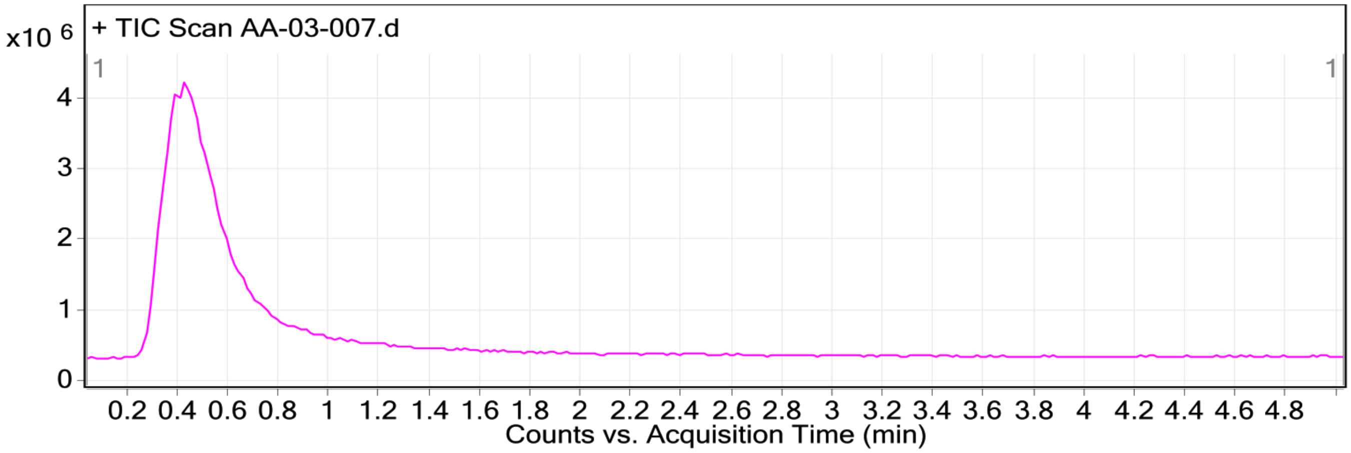

User Spectra

Fragmentor Voltage	Collision Energy	Ionization Mode
175	0	ESI

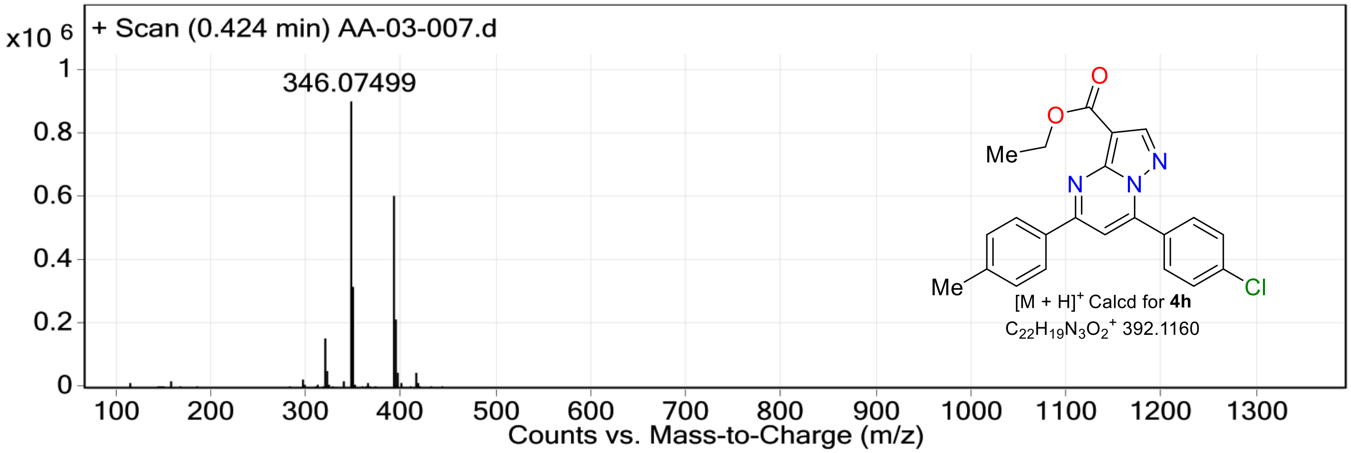

Peak List

m/z	z	Abund
320.09458		158215.9
346.07499	1	904297.8
347.07776	1	208589
348.07207	1	321472.7
349.07469	1	68172.6
392.11628	1	606379.9
393.11898	1	161480.1
394.11455	1	215059

Fig. S39 HRMS analysis of compound **4h**.

User Chromatograms

Fragmentor Voltage 175 **Collision Energy** 0 **Ionization Mode** ESI

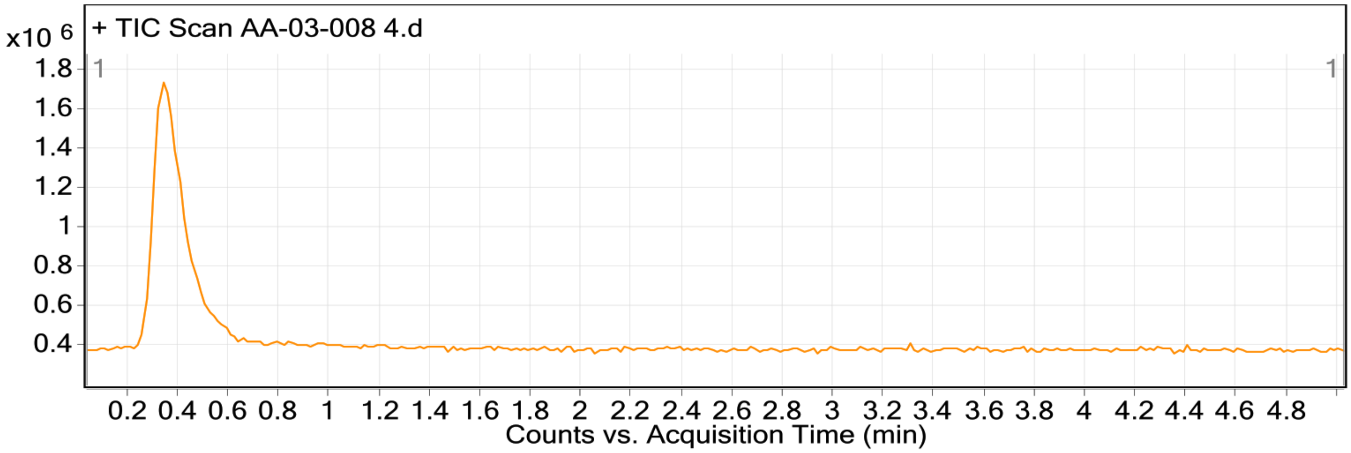

User Spectra

Fragmentor Voltage	Collision Energy	Ionization Mode
175	0	ESI

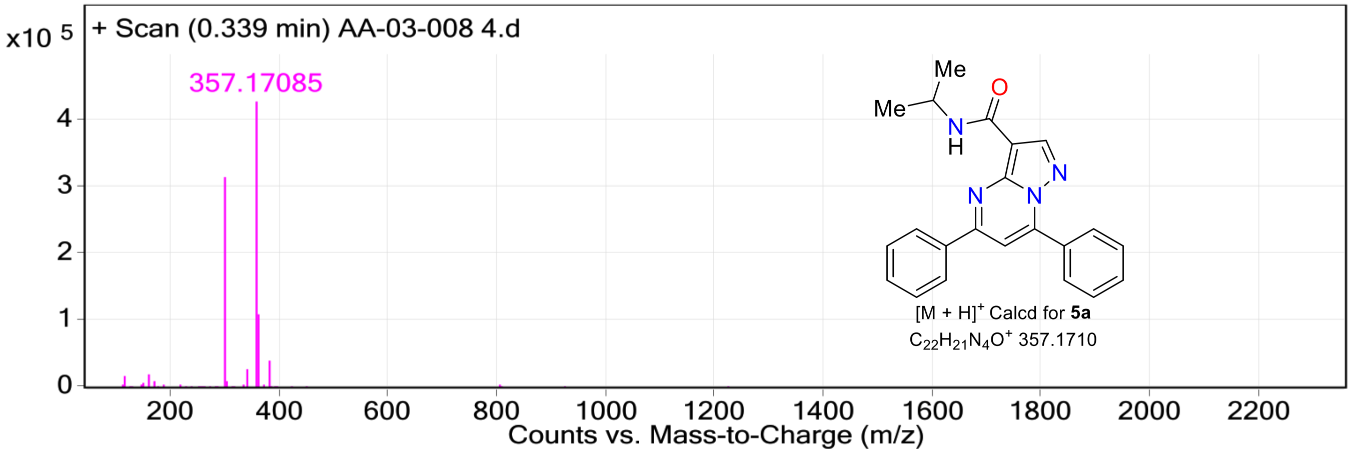

Peak List

m/z	z	Abund
298.09737	1	313778.5
299.10055	1	68831.1
338.34089		28991.5
357.17085	1	428035.1
358.17355	1	109908.2
379.15221		41482.2

Fig. S40 HRMS analysis of compound **5a**.

User Chromatograms

Fragmentor Voltage 175 **Collision Energy** 0 **Ionization Mode** ESI

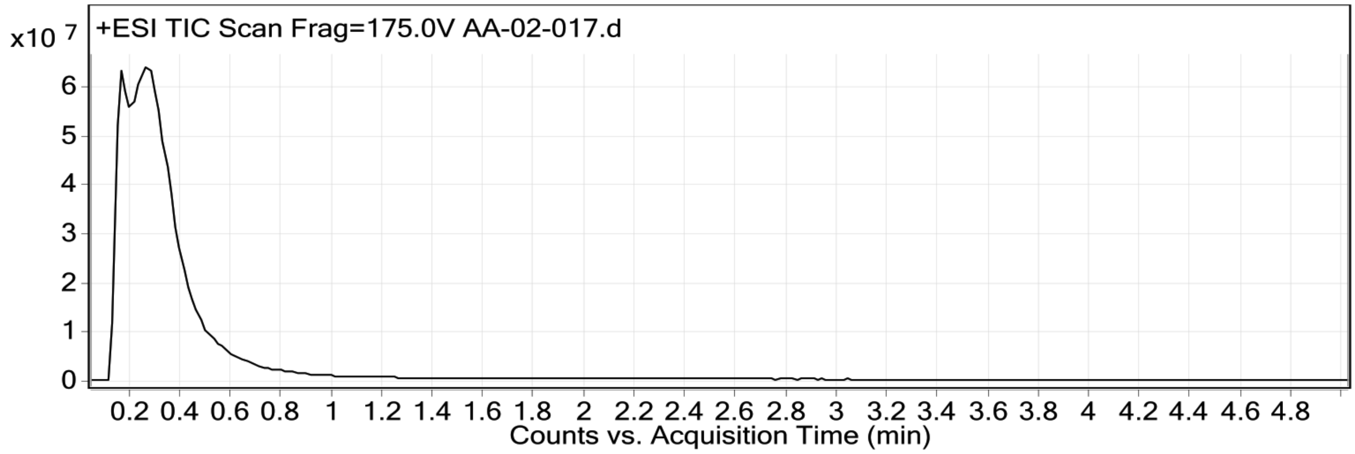

User Spectra

Fragmentor Voltage 175 **Collision Energy** 0 **Ionization Mode** ESI

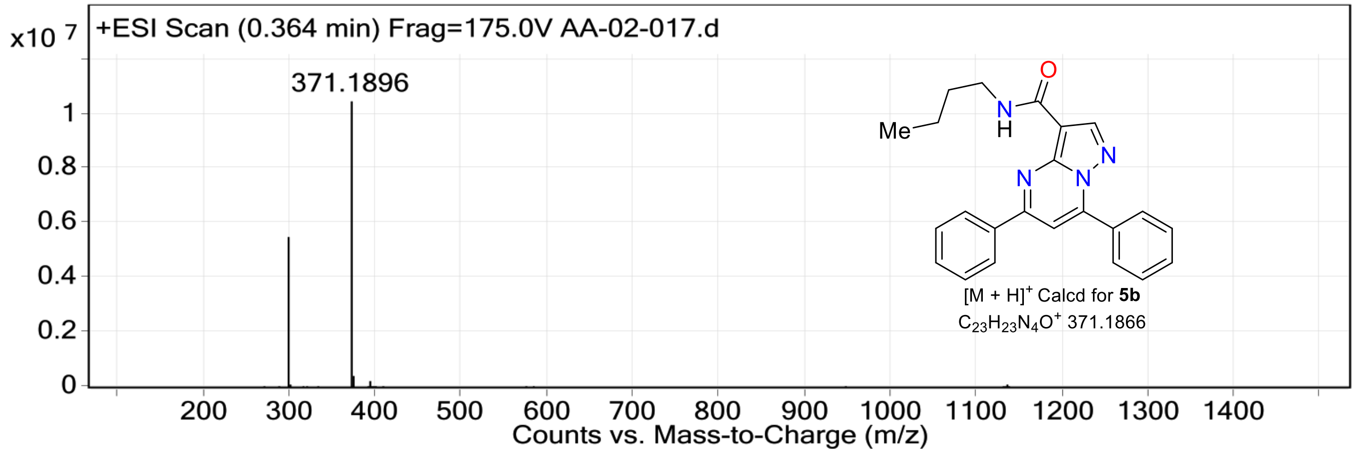

Peak List

m/z	z	Abund
298.0994	1	5520525.5
299.1014	1	1335533.1
371.1896	1	10484356
372.1918	1	3166750.5

Fig. S41 HRMS analysis of compound **5b**.

User Chromatograms

Fragmentor Voltage 175 **Collision Energy** 0 **Ionization Mode** ESI

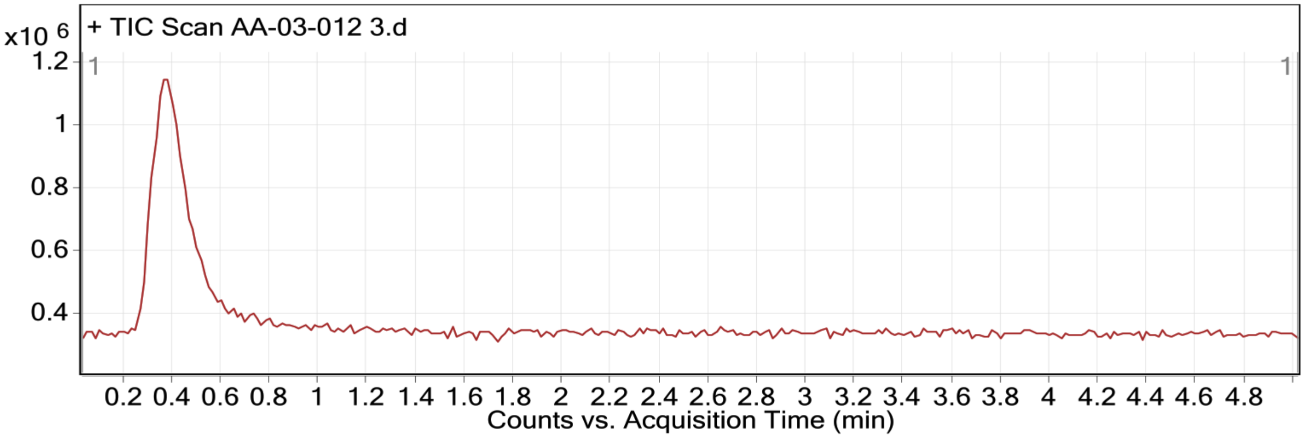

User Spectra

Fragmentor Voltage	Collision Energy	Ionization Mode
175	0	ESI

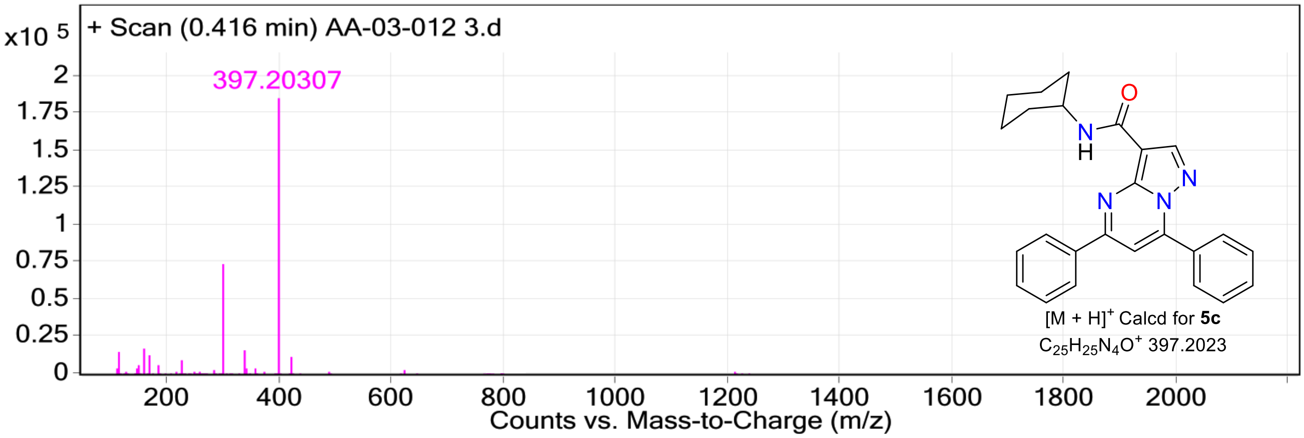

Peak List

m/z	z	Abund
114.09181		14986.6
158.15352		17940.3
167.0516		13571.5
298.0971	1	74181.4
299.10135	1	14521.2
338.3412		16964
397.20307	1	185271.7
398.20582	1	52363.1

Fig. S42 HRMS analysis of compound **5c**.

User Chromatograms

Fragmentor Voltage 175 **Collision Energy** 0 **Ionization Mode** ESI

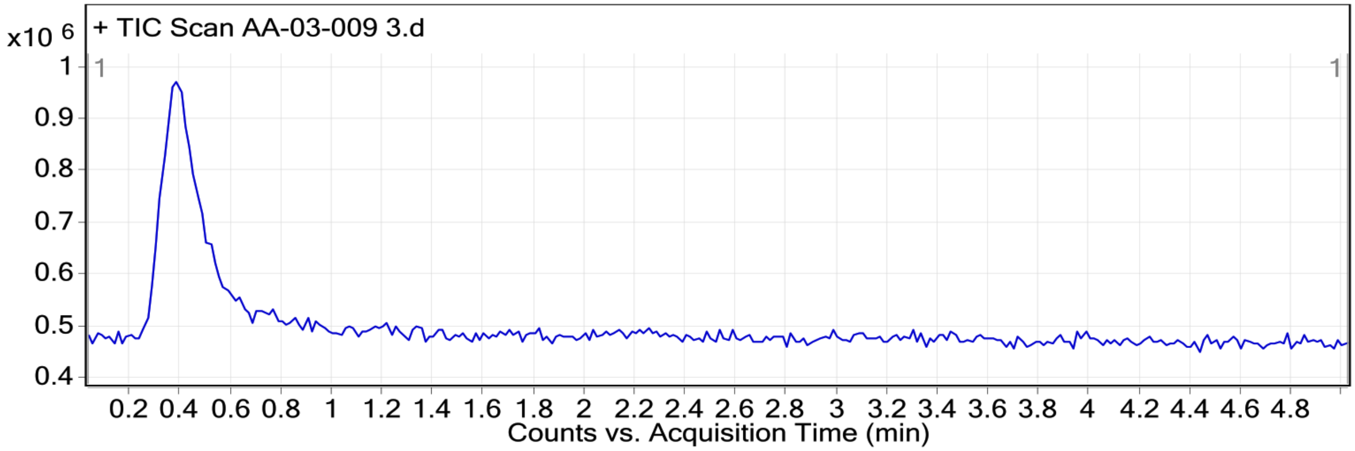

User Spectra

Fragmentor Voltage 175 **Collision Energy** 0 **Ionization Mode** ESI

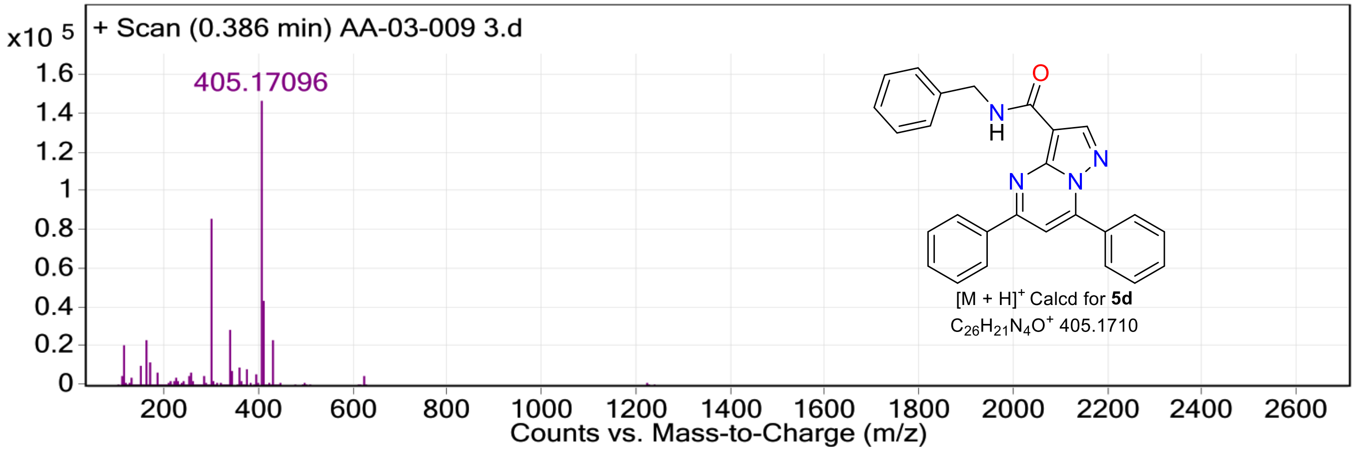

Peak List

m/z	z	Abund
114.09062		21253.7
158.15339		23422.6
167.0518		11901.4
298.0974	1	85991.8
299.09874	1	17985.3
338.34079	1	29034.7
405.17096	1	147035.4
406.17352	1	44041.2
427.15117	1	23563.8

Fig. 43 HRMS analysis of compound **5d**.

User Chromatograms

Fragmentor Voltage 175 **Collision Energy** 0 **Ionization Mode** ESI

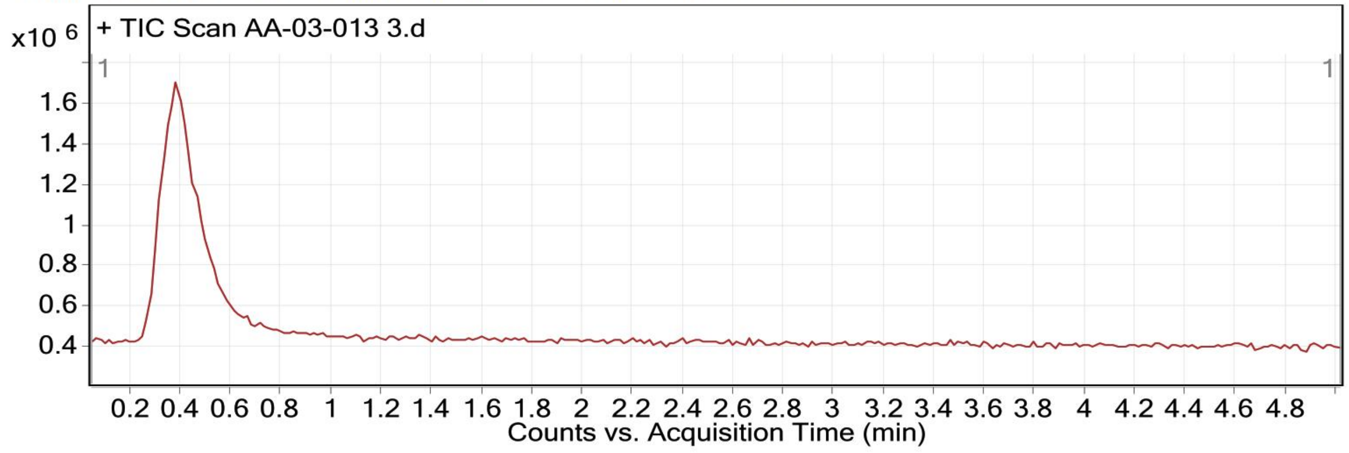

User Spectra

**Fragmentor Voltage**	**Collision Energy**	**Ionization Mode**
175	0	ESI

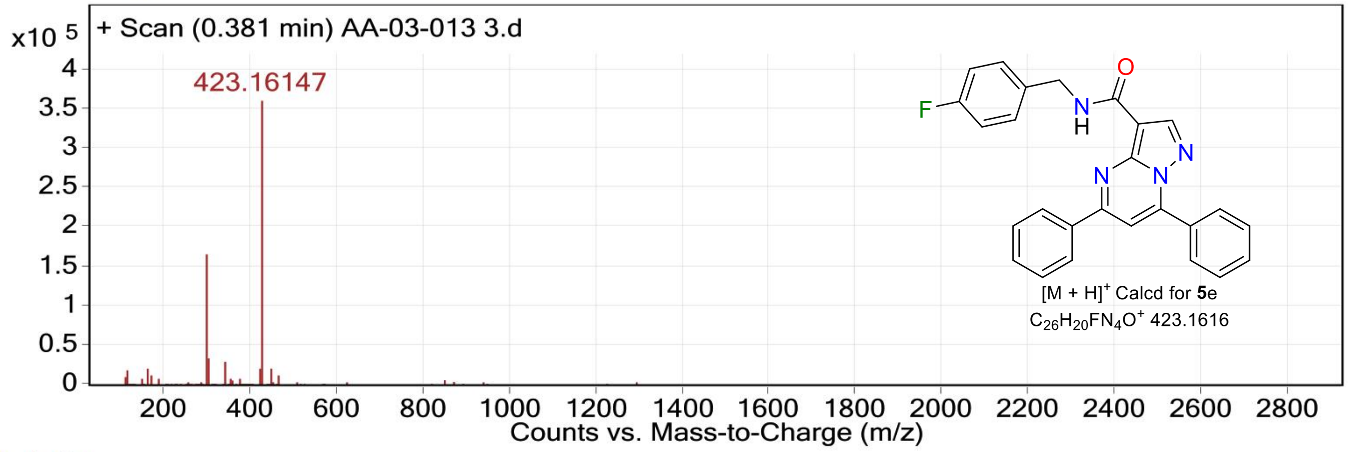

Peak List

m/z	z	Abund
158.15388		21586.7
298.097	1	165114.9
299.10046	1	33654.8
338.34277		29797.4
422.17645		21903.6
423.16147	1	360827.8
424.16439	1	103833
445.14332		22584.7

Fig. 44 HRMS analysis of compound **5e**.

User Chromatograms

Fragmentor Voltage 175 **Collision Energy** 0 **Ionization Mode** ESI

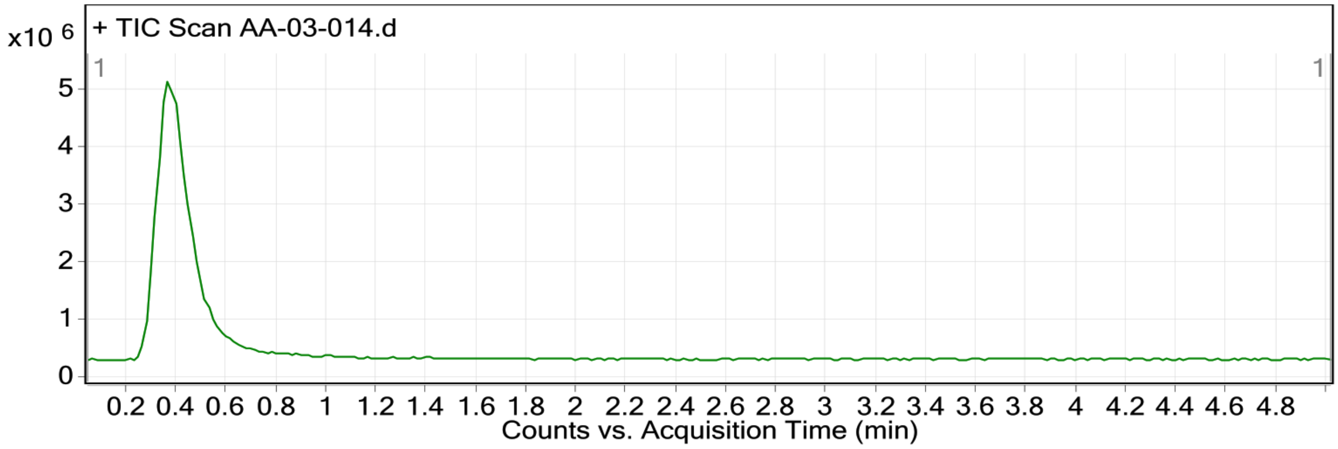

User Spectra

**Fragmentor Voltage**	**Collision Energy**	**Ionization Mode**
175	0	ESI

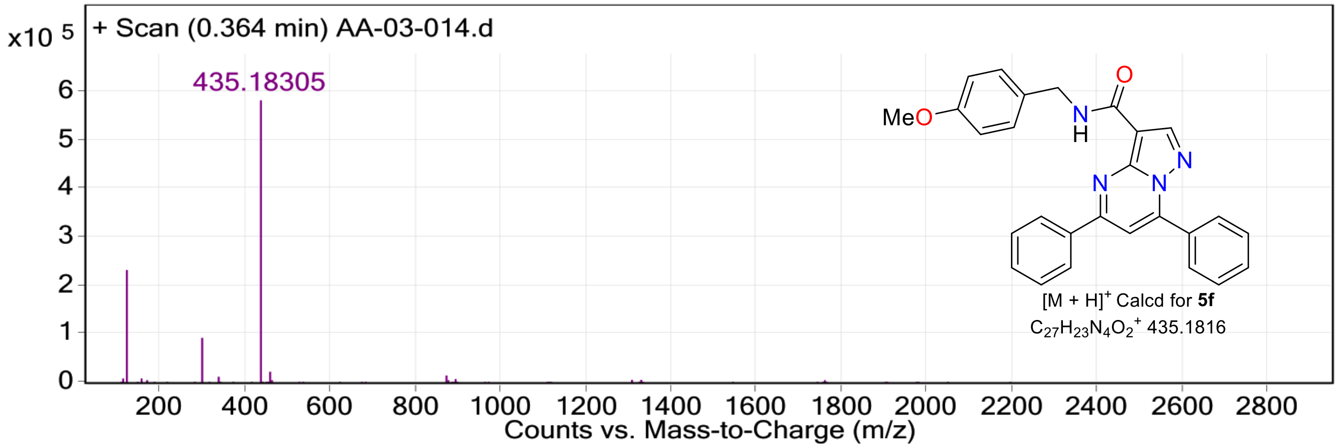

Peak List

m/z	z	Abund
121.06519		235106.6
298.09823		95862
435.18305	1	583501.3
435.28235		36982.6
435.35359		29707.9
436.18589	1	170486.3

Fig. 45 HRMS analysis of compound **5f**.

User Chromatograms

Fragmentor Voltage 175 **Collision Energy** 0 **Ionization Mode** ESI

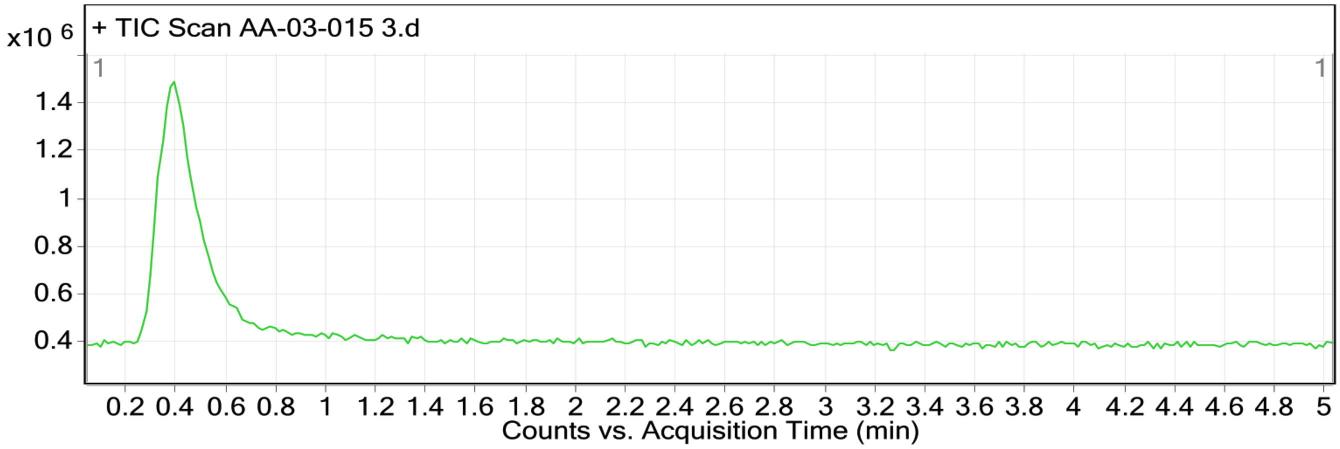

User Spectra

**Fragmentor Voltage**	**Collision Energy**	**Ionization Mode**
175	0	ESI

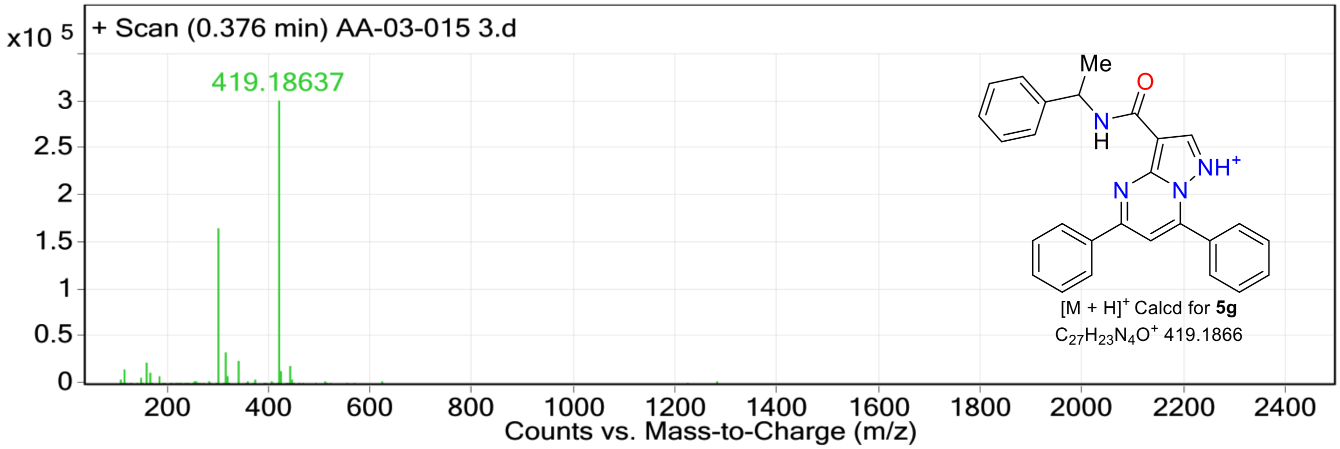

Peak List

m/z	z	Abund
158.15354		23581.6
298.09712	1	166188.3
299.09925	1	34080.2
315.12266		33471.7
338.34141		25467.6
419.18637	1	301629.8
420.18875	1	95401.3
441.16661		20538.3

Fig. 46 HRMS analysis of compound **5g**.

User Chromatograms

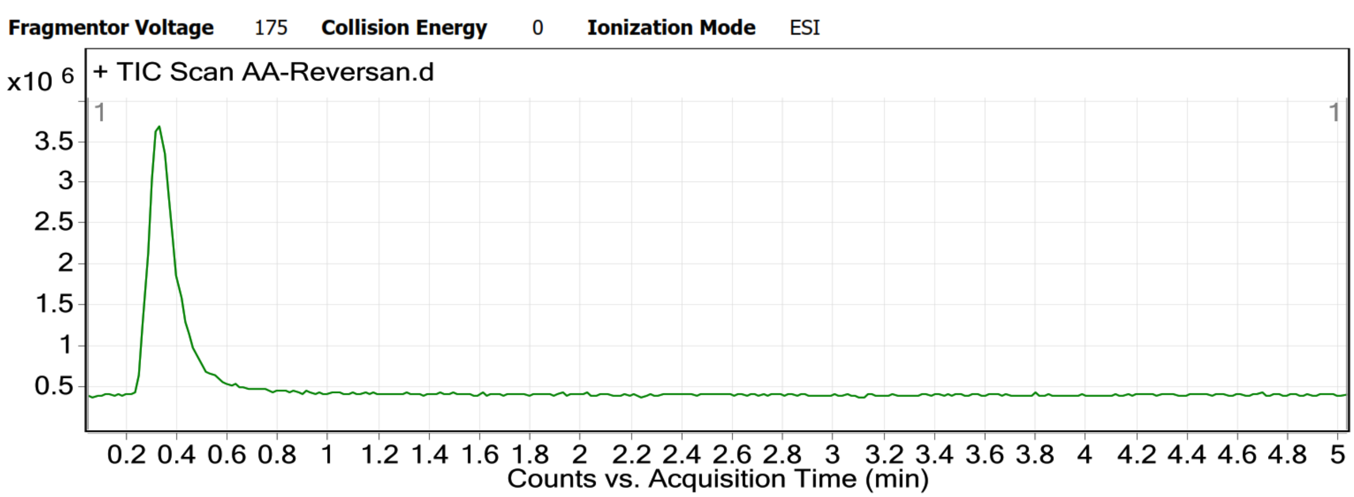

User Spectra

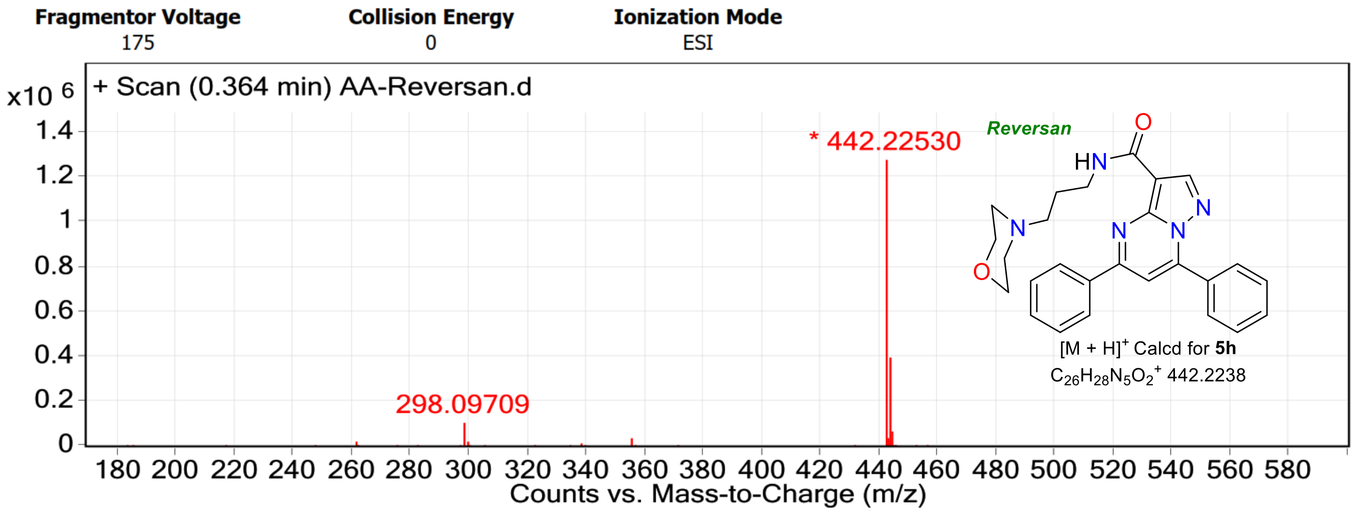

Peak List

m/z	z	Abund
298.09709		103541.2
442.2253	1	1280683.9
443.22761	1	401013.8
444.22924	1	67448.1

Fig. 47 HRMS analysis of compound **5h**.

5. Crystallographic details

Experimental. The X-ray intensity data were measured at 25(2) °C using CuKα radiation (λ = 1.54184 Å), by ω scans in an Agilent SuperNova, Dual, Cu at Zero, Atlas four-circle diffractometer equipped with a CCD plate detector. The collected frames were integrated with the CrysAlis PRO software package (CrysAlisPro 1.171.39.46e, Rigaku Oxford Diffraction, 2018). Data were corrected for the absorption effect using the CrysAlis PRO software package by the empirical absorption correction using spherical harmonics, implemented in the SCALE3 ABSPACK scaling algorithm. Crystal data, data collection, and structure refinement details are summarized in Table S1. H atoms were placed in calculated positions (C—H = 0.93 - 0.98 Å) and included as riding contributions, with isotropic displacement parameters set at 1.2–1.5 times the U_{eq} value of the parent atom. H atoms belonging to NH groups were located in difference density maps and were refined freely. The crystal structures were solved using an iterative algorithm[8] and subsequently completed by a difference Fourier map and refined using the program SHELXL2014.[9] Molecular and supramolecular graphics were carried out using Mercury software.[10]

Table S1 Crystallographic data of compounds 4a, 4g, 5a, 5d, and 5h recorded in a diffractometer at 25(2) °C.

**Data/compound (CF)**[a]	**4a ($C_{21}H_{17}N_3O_2$)**	**4g ($C_{22}H_{18}ClN_3O_2$)**	**5a ($C_{22}H_{20}N_4O$)**	**5d ($C_{26}H_{20}N_4O$)**	**5h ($C_{26}H_{27}N_5O_2$)**
M_r	343.38	391.84	356.42	404.46	441.52
Crystal system, space group	Monoclinic, $P2_1/c$	Monoclinic, $P2_1/c$	Tetragonal, $I4_1/a$	Monoclinic, $P2_1/c$	Orthorhombic, *Pbca*
a, *b*, *c* (Å)	12.2765 (7), 14.1241 (8), 10.5227 (5)	14.4736 (12), 7.8609 (6), 17.8444 (16)	22.9964 (10), 22.9964 (10) 14.1407 (17)	10.4688 (5), 10.5163 (5), 18.8519 (10)	10.4658 (8), 9.9343 (8), 43.524 (3)
α, β, γ (°)	90.0, 105.977 (6), 90.0	90.0, 106.052 (9), 90.0	90.0, 90.0, 90.0	90.0, 100.789 (5), 90.0	90.0, 90.0, 90.0
V (Å^3)	1754.12 (17)	1951.1 (3)	7478.1 (11)	2038.78 (18)	4525.3 (6)
Z	4	4	16	4	8
Radiation type	Cu *K*α	Cu *K*α	Cu *K*α	Cu *K*α	Cu *K*α
μ (mm^{-1})	0.69	1.92	0.64	0.66	0.68
ρ, kg m^{-3}	1.300	1.334	1.266	1.318	1.296
**Data collection** [b]					
No. of measured, independent/observed [$I > 2\sigma(I)$] reflections	14221, 3642, 3045	11940, 4047, 3384	12683, 3831,3350	19639, 4244, 3615	19705, 4693, 4230
R_{int}	0.041	0.066	0.031	0.062	0.041
(sin θ/λ)$_{max}$ (Å^{-1})	0.631	0.630	0.630	0.630	0.630
Theta range for data collection	-15<=h<=14, -17<=k<=17, -8<=l<=13	-18<=h<=17, -9<=k<=6, -22<=l<=22	-27<=h<=28, -28<=k<=19, -14<=l<=17	-9<=h<=13, -13<=k<=13, -23<=l<=23	-12<=h<=13, -11<=k<=12, -53<=l<=54
Theta range for data collection	3.745 to 76.574	5.158 to 76.372	3.669 to 76.205	4.299 to 76.360	4.063 to 76.343
**Refinement**					
$R[F^2 > 2\sigma(F^2)]$, $wR(F^2)$, S	0.050, 0.140, 1.03	0.068, 0.220, 1.06	0.051, 0.145, 1.04	0.055, 0.162, 1.06	0.054, 0.155, 1.04
No. of reflections	3642	4047	3831	4244	4693
No. of parameters	237	256	249	283	301
No. of restraints	0	0	15	0	0
H-atom treatment	H-atom parameters constrained	H-atom parameters constrained	By a mixture of independent and constrained refinement	By a mixture of independent and constrained refinement	By a mixture of independent and constrained refinement
$\Delta\rho_{max}$, $\Delta\rho_{min}$ (e Å^{-3})	0.19, −0.16	0.31, −0.28	0.26, −0.47	0.17, −0.23	0.25, −0.22

[a] CF = Chemical formula. [b] A diffractometer SuperNova was used. Dual, Cu at zero, Atlas. Multi-scan (CrysAlis PRO; Agilent, 2014) was used as an absorption correction.

Structural commentary. Molecular structures of **4a**, **4g**, **5a**, **5d**, and **5h** were obtained from the structural determination using X-ray diffraction data (Fig. 48). Structures are shown with anisotropic thermal vibration

ellipsoids drawn at the 50% probability level. The hydrogen atoms are shown as spheres of arbitrary radius. The structural determinations and the corresponding refinements were performed without ambiguity. The final R-values are shown in Table S1. In amides **5a**, **5d**, and **5h**, the molecular conformations are influenced by an intramolecular N-H···N hydrogen bond involving the amine group and the pyrazolo[1,5-*a*]pyrimidine ring with H···N distances between 2.1 – 2.3 Å (Fig. S48). The non-bonding electron pairs over the oxygen atom in the carbonyl group play an essential role in the supramolecular structures. In esters **4a** and **4g**, C-H···O hydrogen interactions connect molecules in the crystal. However, the chlorine atom in **4g** changes the acid character of the hydrogen atoms, inducing that in **4a**, the hydrogen interaction is mediated by the phenyl group. In comparison, in **4g**, this interaction involves the methylene moiety. This behaviour is additionally observed in the H···O distances, 2.52 and 2.80 Å for **4a** and **4g**, respectively (Fig. S49).

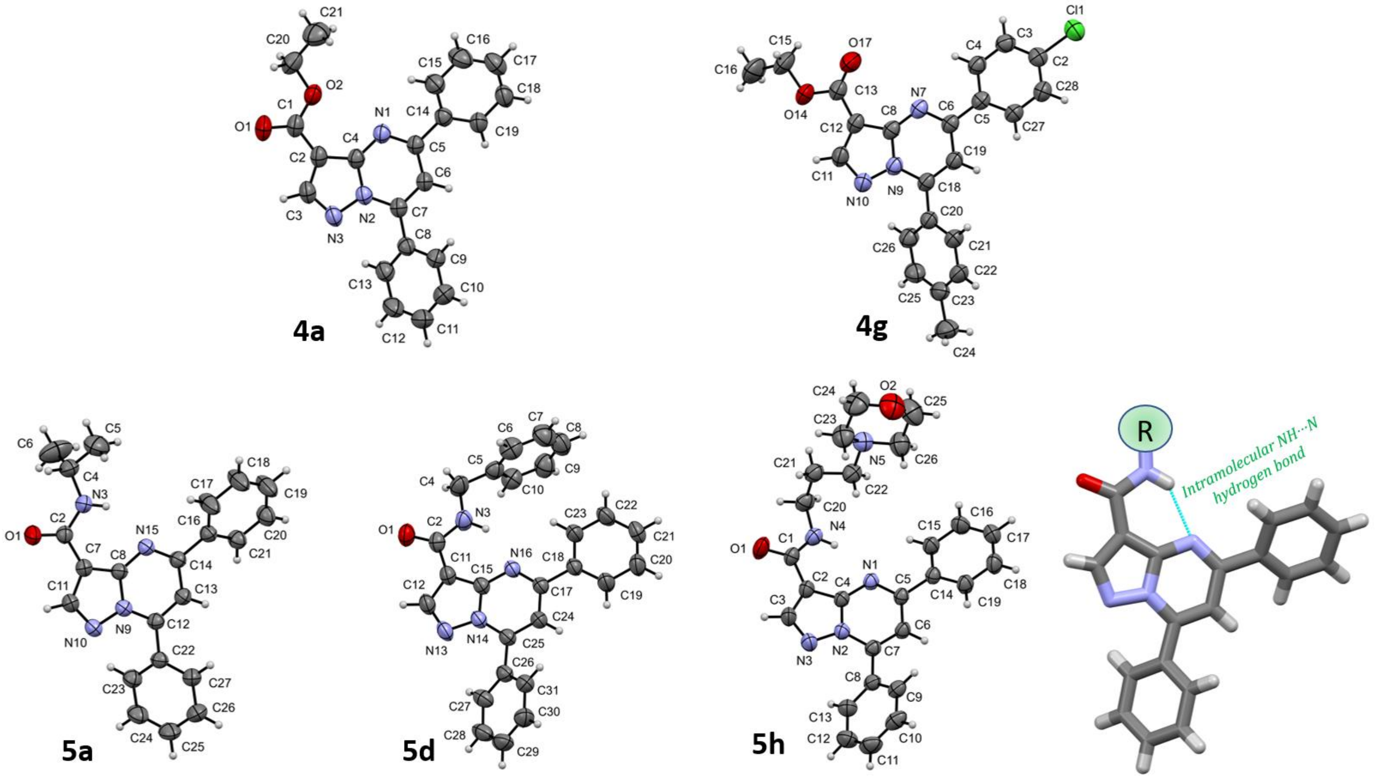

Fig. S48 Molecular structures of **4a**, **4g**, **5a**, **5d**, and **5h** obtained by X-ray diffraction data

On the other hand, in compounds **5a**, **5d**, and **5h** (Reversan), the participation of the carbonyl group (C=O) is crucial in the supramolecular structure. However, the amide group instead of the ester group produces changes in connectivity. In **5a**, the shortest C-H···O hydrogen bond involves the pyrazolo[1,5-*a*]pyrimidine ring (H···O distance of 2.53 Å), while in **5d**, this interaction consists of the phenyl ring (H···O distance of 2.63 Å). In **5h**, the shortest hydrogen interaction occurs between two neighbouring amide groups by N-H···O hydrogen bonds (H···O distance of 2.53 Å) (Fig. S50).

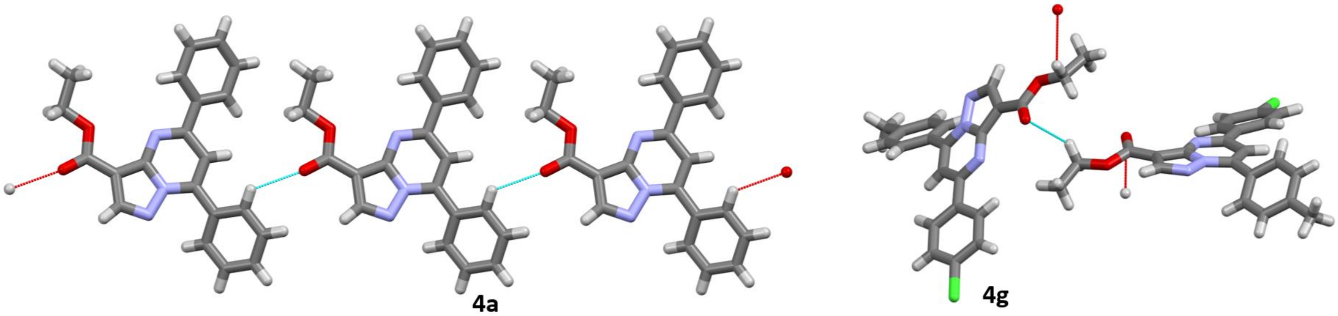

4a 4g

Fig. S49 Hydrogen interactions (C-H···O) connect molecules in the crystal for esters **4a** and **4g**.

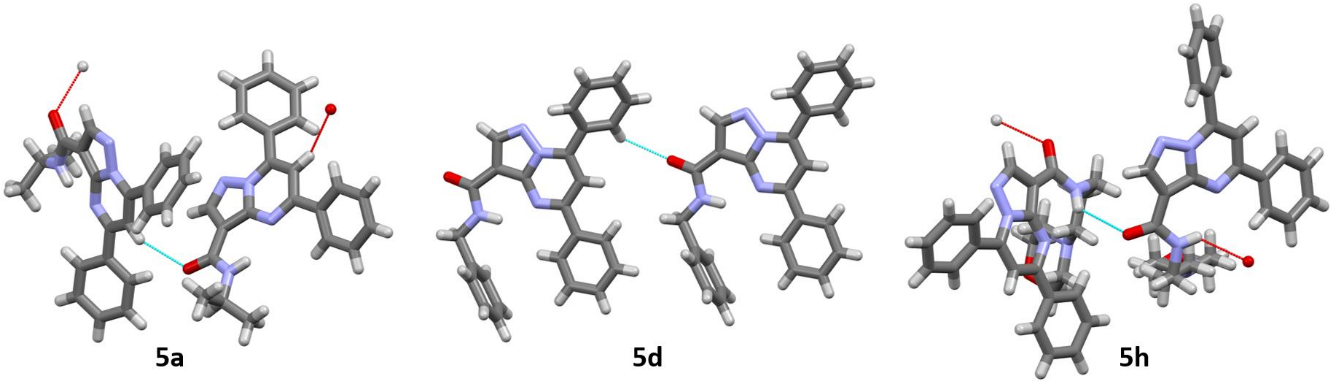

5a 5d 5h

Fig. S50 Hydrogen interactions (C-H···O) connect molecules in the crystal for amides **5a**, **5d**, and **5h** (Reversan).

6. References

1 A. K. A.K., Y. D. Bodke, A. N. Gowda, G. Sambasivam and K. G. Bhat, *J Heterocycl. Chem,* 2017, **54**, 1904–1924.

2 A. K. Ajeesh Kumar, K. B. Nair, Y. D. Bodke, G. Sambasivam and K. G. Bhat, *Monatsh. Chem.*, 2016, **147**, 2221–2234.

3 N. L. Nam, I. I. Grandberg and V. I. Sorokin, *Chem. Heterocycl. Compd.*, 2002, **38**, 1371–1374.

4 S. Louiz, H. Labiadh and R. Abderrahim, *Spectrochim. Acta A Mol. Biomol.*, 2015, **134**, 276–282.

5 N. Upadhyay, K. Tilekar, F. Loiodice, N. Yu. Anisimova, T. S. Spirina, D. V. Sokolova, G. B. Smirnova, J. Choe, F.-J. Meyer-Almes, V. S. Pokrovsky, A. Lavecchia and C. Ramaa, *Bioorg. Chem.*, 2021, **107**, 104527.

6 Z. Gonda and Z. Novák, *Chem. Eur. J.*, 2015, **21**, 16801–16806.

7 M. Zhang, J. Xi, R. Ruzi, N. Li, Z. Wu, W. Li and C. Zhu, *J. Org. Chem.*, 2017, **82**, 9305–9311.

8 L. Palatinus and G. Chapuis, *J. Appl. Crystallogr.*, 2007, **40**, 786–790.

9 G. M. Sheldrick, *Acta Crystallogr., Sect. C: Struct. Chem.*, 2015, **C71**, 3–8.

10 C. F. Macrae, I. J. Bruno, J. A. Chisholm, P. R. Edgington, P. McCabe, E. Pidcock, L. Rodriguez-Monge, R. Taylor, J. van de Streek and P. A. Wood, *J. Appl. Crystallogr.*, 2008, **41**, 466–470.
